# Effectiveness of Lumbar Cerebrospinal Fluid Drain Among Patients With Aneurysmal Subarachnoid Hemorrhage: A Randomized Clinical Trial

**DOI:** 10.1001/jamaneurol.2023.1792

**Published:** 2023-06-18

**Authors:** Stefan Wolf, Dorothee Mielke, Christoph Barner, Vesna Malinova, Thomas Kerz, Maria Wostrack, Patrick Czorlich, Farid Salih, Doortje C. Engel, Angelika Ehlert, Dimitre Staykov, Abdulrahman Y. Alturki, Ulrich Sure, Jürgen Bardutzky, Henry W. S. Schroeder, Ludwig Schürer, Jürgen Beck, Tareq A. Juratli, Michael Fritsch, Johannes Lemcke, Anne Pohrt, Bernhard Meyer, Stefan Schwab, Veit Rohde, Peter Vajkoczy

**Affiliations:** 1 Department of Neurosurgery, Charité–Universitätsmedizin Berlin, corporate member of Freie Universität Berlin, Humboldt-Universität zu Berlin, and Berlin Institute of Health, Berlin, Germany; 2 Department of Neurosurgery, University Medical Center Göttingen, Göttingen, Germany; 3 Department of Anesthesiology, Charité–Universitätsmedizin Berlin, corporate member of Freie Universität Berlin, Humboldt-Universität zu Berlin, and Berlin Institute of Health, Berlin, Germany; 4 Department of Neurosurgery, University Medical Center, Johannes Gutenberg University, Mainz, Germany; 5 Department of Neurosurgery, Technical University Munich, Munich, Germany; 6 Department of Neurosurgery, Hamburg University Medical Center, Hamburg, Germany; 7 Department of Neurology, Charité–Universitätsmedizin Berlin, corporate member of Freie Universität Berlin, Humboldt-Universität zu Berlin, and Berlin Institute of Health, Berlin, Germany; 8 Department of Neurosurgery, Cantonal Hospital St Gallen, St Gallen, Switzerland; 9 Department of Neurosurgery, Asklepios Hospital St Georg, Hamburg, Germany; 10 Department of Neurology, University Medical Center Erlangen-Nuremberg, Erlangen, Germany; 11 Department of Neurology, Hospital of the Brothers of St John, Eisenstadt, Austria; 12 Department of Neurology and Neurosurgery, Montreal Neurological Institute and Hospital, McGill University, Montreal, Quebec, Canada; 13 Neurovascular Surgery Section, Adult Neurosurgery Department, National Neuroscience Institute, King Fahad Medical City, Riyadh, Saudi Arabia; 14 Department of Neurosurgery and Spine Surgery, University Hospital Essen, Essen, Germany; 15 Department of Neurology, University of Freiburg, Freiburg, Germany; 16 Department of Neurosurgery, University Medicine Greifswald, Greifswald, Germany; 17 Department of Neurosurgery, Klinikum Bogenhausen, Technical University Munich, Munich, Germany; 18 Department of Neurosurgery, University of Freiburg, Freiburg, Germany; 19 Department of Neurosurgery, Inselspital, University of Bern, Switzerland; 20 Department of Neurosurgery, University Hospital Carl Gustav Carus, Technische Universität Dresden, Dresden, Germany; 21 Department of Neurosurgery, Dietrich Bonhoeffer Klinikum, Neubrandenburg, Germany; 22 Department of Neurosurgery, Unfallkrankenhaus Berlin, Berlin, Germany; 23 Department of Medical Biometrics, Charité–Universitätsmedizin Berlin, corporate member of Freie Universität Berlin, Humboldt-Universität zu Berlin, and Berlin Institute of Health, Berlin, Germany

## Abstract

**Importance:**

After aneurysmal subarachnoid hemorrhage, the use of lumbar drains has been suggested to decrease the incidence of delayed cerebral ischemia and improve long-term outcome.

**Objective:**

To determine the effectiveness of early lumbar cerebrospinal fluid drainage added to standard of care in patients after aneurysmal subarachnoid hemorrhage.

**Design, Setting, and Participants:**

The EARLYDRAIN trial was a pragmatic, multicenter, parallel-group, open-label randomized clinical trial with blinded end point evaluation conducted at 19 centers in Germany, Switzerland, and Canada. The first patient entered January 31, 2011, and the last on January 24, 2016, after 307 randomizations. Follow-up was completed July 2016. Query and retrieval of data on missing items in the case report forms was completed in September 2020. A total of 20 randomizations were invalid, the main reason being lack of informed consent. No participants meeting all inclusion and exclusion criteria were excluded from the intention-to-treat analysis. Exclusion of patients was only performed in per-protocol sensitivity analysis. A total of 287 adult patients with acute aneurysmal subarachnoid hemorrhage of all clinical grades were analyzable. Aneurysm treatment with clipping or coiling was performed within 48 hours.

**Intervention:**

A total of 144 patients were randomized to receive an additional lumbar drain after aneurysm treatment and 143 patients to standard of care only. Early lumbar drainage with 5 mL per hour was started within 72 hours of the subarachnoid hemorrhage.

**Main Outcomes and Measures:**

Primary outcome was the rate of unfavorable outcome, defined as modified Rankin Scale score of 3 to 6 (range, 0 to 6), obtained by masked assessors 6 months after hemorrhage.

**Results:**

Of 287 included patients, 197 (68.6%) were female, and the median (IQR) age was 55 (48-63) years. Lumbar drainage started at a median (IQR) of day 2 (1-2) after aneurysmal subarachnoid hemorrhage. At 6 months, 47 patients (32.6%) in the lumbar drain group and 64 patients (44.8%) in the standard of care group had an unfavorable neurological outcome (risk ratio, 0.73; 95% CI, 0.52 to 0.98; absolute risk difference, −0.12; 95% CI, −0.23 to −0.01; *P* = .04). Patients treated with a lumbar drain had fewer secondary infarctions at discharge (41 patients [28.5%] vs 57 patients [39.9%]; risk ratio, 0.71; 95% CI, 0.49 to 0.99; absolute risk difference, −0.11; 95% CI, −0.22 to 0; *P* = .04).

**Conclusion and Relevance:**

In this trial, prophylactic lumbar drainage after aneurysmal subarachnoid hemorrhage lessened the burden of secondary infarction and decreased the rate of unfavorable outcome at 6 months. These findings support the use of lumbar drains after aneurysmal subarachnoid hemorrhage.

**Trial Registration:**

ClinicalTrials.gov Identifier: NCT01258257

## Key Points

**Question:**

Does prophylactic lumbar cerebrospinal fluid drainage improve clinical outcomes measured by the modified Rankin Scale score among patients with aneurysmal subarachnoid hemorrhage?

**Findings:**

In this pragmatic randomized clinical trial including 287 patients at 19 sites in 3 countries, the rate unfavorable neurologic outcome was 32.6% in the lumbar drainage group (47 of 144) and 44.8% in the standard of care group (64 of 143), a significant difference.

**Meaning:**

In this trial, among patients with aneurysmal subarachnoid hemorrhage, lumbar drainage improved clinical neurological outcomes at 6 months.

## Teaser

This pragmatic randomized clinical trial evaluates the effectiveness of early lumbar cerebrospinal fluid drainage added to standard of care in patients after aneurysmal subarachnoid hemorrhage.

## Introduction

Subarachnoid hemorrhage from the rupture of an intracranial aneurysm is a type of stroke leading to death or permanent disability in most affected patients.^1,2^ For decades, cerebral vasospasm triggered by the amount of blood in the basal cisterns was regarded as causal for delayed cerebral ischemia.^3^ Approximately 70% of patients with subarachnoid hemorrhage develop vasospasm; up to 40% experience secondary infarction, part of these without vasospasm. Treatment of vasospasm in the large cerebral arteries did not improve mortality or functional outcome.^4,5^ Prophylaxis with the calcium channel blocker nimodipine does not affect the cerebral vasculature but lessens poor outcome by one-third.^6^

It is common standard to occlude the culprit aneurysm by surgical clipping or endovascular coiling within 24 to 48 hours after hemorrhage, with coiling being preferred if both methods are equally feasible.^7^ Efforts to remove the blood in the basal cisterns as the causative agent for vasospasm by surgery, cisternal, or external ventricular drainage showed mixed results.^8,9,10^ In retrospective studies, prophylactic lumbar drainage of cerebrospinal fluid was associated with favorable outcome.^11,12^ A plausible mechanism of action is increased removal of blood and its degradation products using gravity. However, the prospective Lumbar Drainage in Subarachnoid Haemorrhage (LUMAS) trial randomizing 210 patients was unable to confirm a benefit of lumbar drains.^13^ In hindsight, it recruited less severely affected patients with lower risk of adverse outcomes and may thus have been underpowered to detect a significant effect.

We designed the EARLYDRAIN trial to investigate the effect of a lumbar cerebrospinal fluid drainage among patients with a ruptured cerebral aneurysm. Our hypothesis was that early application of a lumbar drain leads to an improved outcome after subarachnoid hemorrhage, measured by the modified Rankin Scale (mRS) score at 6 months.^14^

## Methods

### Trial Design and Oversight

The EARLYDRAIN trial was a pragmatic, multicenter, parallel-group, open-label randomized clinical trial with blinded end point evaluation performed in 19 hospitals in Germany, Switzerland, and Canada. Trial sites were referral centers that provided acute neurosurgical and neurocritical care for patients with subarachnoid hemorrhage on a 24-hour basis with at least 30 aneurysm procedures per year. The protocol included a statistical analysis proposal and was published at the start of the trial,^14^ with no changes or amendments performed later. An independent steering committee consisting of 4 senior investigators (S. W., J. B., S. S., and P. V.) and an independent data monitoring and safety committee of 2 experienced researchers reviewed the trial on conduct and safety. The trial protocol can be found in Supplement 1. This study followed the Consolidated Standards of Reporting Trials (CONSORT) reporting guideline.

Lead ethics approval was obtained from the research Ethics Committee of the University of Erlangen, Germany. The trial was approved locally from the corresponding ethics board at each participating center. If possible, written informed consent was obtained from the patient before the aneurysm treatment procedure. In patients unable to consent, a legally authorized representative was asked for permission. If this person required court approval not available in due time, a physician not involved in the EARLYDRAIN trial was allowed to consent as a surrogate on the presumed will of a capable person, according to German law. Later stepwise approval by the designated person and finally the patient themselves was obtained. In Switzerland and Canada, approval for study inclusion was granted by the next of kin, as required by local laws.

### Participants

To be eligible, patients had to be 18 years or older and present with acute subarachnoid hemorrhage diagnosed by computed tomography (CT) and confirmation of an intracranial aneurysm by CT angiography or digital subtraction angiography. Aneurysm treatment was required to be performed within 48 hours after subarachnoid hemorrhage. Exclusion criteria included contraindications for placement of a lumbar drain, notably absent or compressed basal cisterns on admission CT or the presence of therapeutic anticoagulation, pregnancy, participation in another interventional trial, reduced life expectancy, and hemorrhage of other than aneurysmal origin (eTable 1 in Supplement 2).

### Randomization

Eligible patients were randomized in a 1:1 ratio to receive either standard of care or the additional use of a lumbar drain. Randomization was performed via an internet randomizer.^15^ The method used was permutated blocks with an undisclosed block size of 6 and compensation for eventual rejections. Stratified randomization was not used.

### Data Collection

Patients included in the EARLYDRAIN trial were followed up to their death or 6 months after randomization. Primary documentation was paper based and entered centrally in a database after finishing the recruitment phase. We collected baseline demographic and processes-of-care data from the first 8 days, descriptive radiologic imaging, and 6 months’ outcome.

### Trial Procedures

Emergency treatment on admission using intubation and/or placement of an external ventricular drain was at the discretion of the local team. We performed aneurysm treatment with coiling or clipping, as applicable and per local standard and in concordance with international guidelines and recommendations.^16,17^

In case of randomization to the lumbar drain group, a lumbar drain was placed in sterile technique after aneurysm treatment. Lumbar cerebrospinal fluid diversion was started after a postprocedural CT scan indicated safety. A rate of 5 mL per hour was recommended for the first 8 days. Protocol-compliant treatment required at least 4 days, resulting in an equivalent of 480 mL of lumbar cerebrospinal fluid drainage for per-protocol analysis. Additional diversion via external ventricular drainage was at the discretion of the local team. Intracranial pressure (ICP) monitoring was performed as per local standard. Zeroing of both ventricular and lumbar drains was similar on the level of the external acoustic channel. This facilitated a valid ICP reading of all drains and allowed to recognize a developing craniocaudal gradient as indication for excess lumbar drainage.^18^ In case of a difference of more than 5 mm Hg between both drains or an ICP level greater than 20 mm Hg, we recommended to postpone lumbar cerebrospinal fluid diversion for safety reasons.

Daily transcranial Doppler monitoring was performed as per local standard. In case of suspected vasospasm or routinely on day 7 to 10 after the initial subarachnoid hemorrhage, vascular imaging either via CT angiography, magnetic resonance angiography, or conventional digital subtraction angiography was scheduled. Treatment of confirmed vasospasm could include balloon angioplasty or local intra-arterial vasodilators, with application being at the discretion of the local investigators and not specified in the EARLYDRAIN trial protocol. Due to the interventional character of the trial, blinding of acute caregivers, except local radiologists, was not possible.

### Primary and Secondary End Points

Primary end point was the rate of unfavorable neurological outcome at 6 months after subarachnoid hemorrhage measured with the mRS.^19^ The mRS is a 7-point score ranging from 0 (healthy without compromise) to 6 (death). The score was obtained by an investigator of the local study team not involved in the acute care and blinded to the clinical course of the patient either via telephone interview or by personal visit. For the primary end point, the mRS was dichotomized to either 0 to 2 (good outcome) or 3 to 6 (unfavorable outcome).

The main secondary end point was the rate of secondary infarctions not being present in the postprocedural CT scan performed after aneurysm occlusion. Secondary infarctions were diagnosed with the last cerebral imaging (either CT or magnetic resonance imaging) before discharge from acute care. Radiologists evaluating the scans received no information on treatment groups. Further secondary end points included the rate of mortality in both groups; the Barthel score^20^ and the Glasgow Outcome Scale–Extended score^21^ after 6 months; the mRS after 6 months depicted as a continuous variable; the rates of vasospasm assumed clinically by transcranial Doppler (threshold, 160 cm/s; mean flow in middle cerebral artery at 50-60 mm depth) and by angiography; the requirement of a permanent ventriculoperitoneal shunt; and the rate of infections, including device-associated meningitis. No changes in end points and their evaluation were performed after the start of the trial.

### Statistical Analysis

For sample size calculation in the study planning phase, available retrospective studies were of questionable external validity due to their exceptionally low mortality. Data from the prospective LUMAS trial were not yet available.^14^ We calculated 300 patients being required to detect a decrease in the rate of unfavorable outcome from 50% to 33% with a power of 85% and 5% α error, allowing for imbalances between groups.

All statistics were performed with R version 4.1.0 (The R Foundation). Missing data were not imputed. Group differences of categorical variables were tested with the χ^2^ test. Continuous variables are reported as medians and IQRs; testing was performed with the Mann-Whitney *U* test. Daily data were analyzed with mixed models for repeated measurements, with Satterthwaite *df* method being used to derive *P* values for unevenly balanced data.^22^

Intention-to-treat analysis included all patients meeting all inclusion criteria and without exclusion criteria, regardless of their actual treatment. We performed additional sensitivity analysis in patients treated per protocol and as treated. Per-protocol analysis included all patients in the lumbar drain group with 480 mL or more drainage via lumbar route in the first 8 days and all patients in the standard of care group without a lumbar drain or, in case this was violated, receiving less than 480 mL of lumbar drainage in the first 8 days. The as-treated analysis was performed using all data according to the actual treatment, considering the patients being excluded in the per-protocol analysis in the corresponding other group.

We used logistic regression for analysis of primary and secondary end points to allow for easy expansion to multivariate assessment. Odds ratios were transformed to risk ratios for more appropriate interpretation.^23^ Analyses on the dichotomized mRS scores were performed both unadjusted and adjusted for age, Hunt-Hess grade on admission, and presence or absence of intraventricular and intraparenchymal hemorrhages as relevant confounders. Additionally, we tried other potential variables alone and in combination. Statistical models were compared using the minimized Akaike information criterion.^24^ Mortality between groups was compared with Cox proportional hazards.

Adjustment for multiple testing was not performed. Therefore, we regard all analysis beyond the primary end point as exploratory. Significance tests on secondary end points and other variables are provided to illuminate potential signals in the data and not for causal inference. A 2-sided *P* < .05 was used to indicate statistical significance.

## Results

### Patient Characteristics

**Figure 1.**
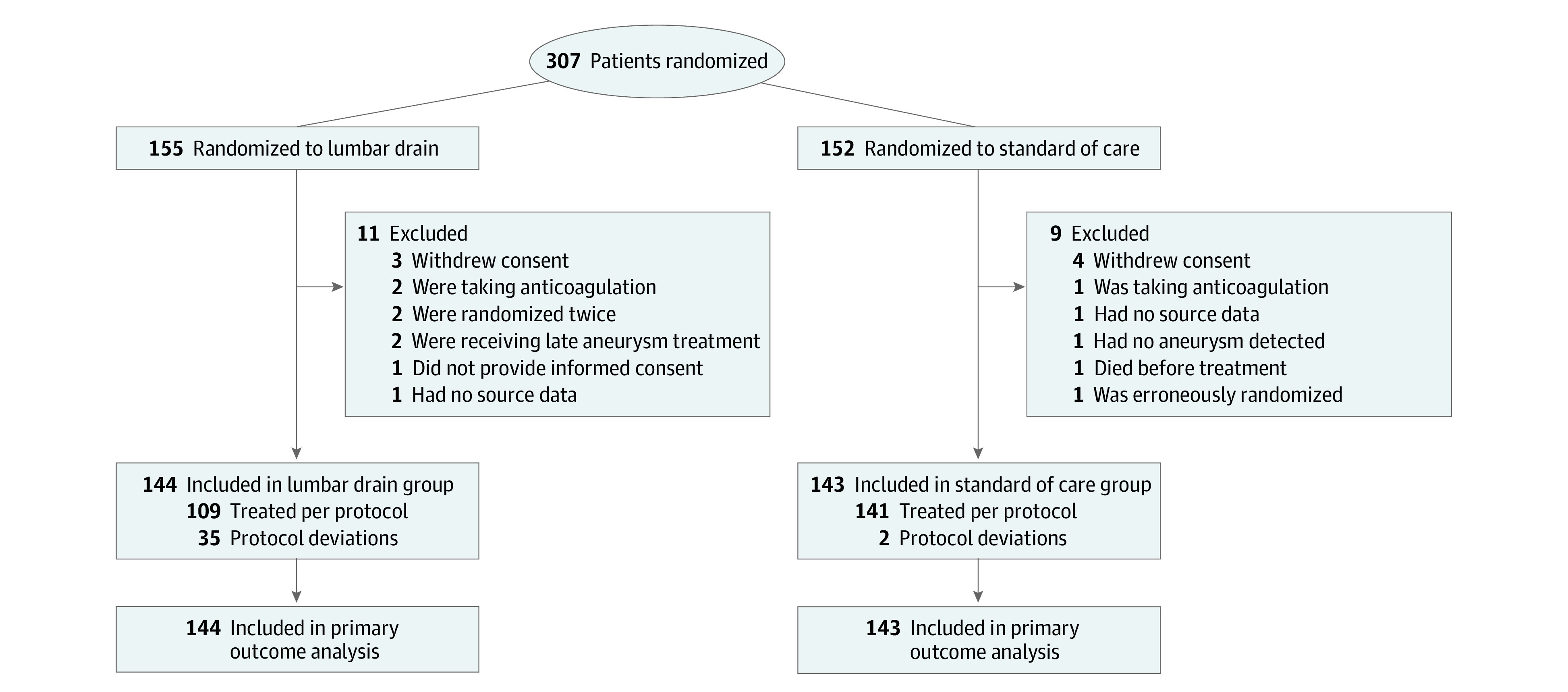
CONSORT Diagram Patients were screened by acute care clinicians from the affiliated centers, mainly the departments of neurology and neurosurgery. Numbers of screened patients were not recorded in all affiliated hospitals (eTable 2 in Supplement 2).

The first patient was enrolled January 31, 2011. After 300 randomizations, 7 allocation failures were known, and the data monitoring and safety board decided to replace these. On January 24, 2016, recruitment in the EARLYDRAIN trial finished after 307 randomizations. A total of 152 randomizations indicated standard of care treatment and 155 were referred to additional placement of a lumbar drain. Eleven randomizations in the lumbar drain group and 9 in the standard of care group lacked complete inclusion criteria or had an exclusion criterion emerging. Main reasons for invalid randomizations were lack of or withdrawn informed consent and a requirement of high-grade therapeutic anticoagulation unforeseeable at the time of randomization. (Figure 1; eFigure 2 in Supplement 2).

Of 287 included patients, 197 (68.6%) were female, and the median (IQR) age was 55 (48-63) years. A total of 287 randomized patients were analyzed according to the intention-to-treat principle, with 144 in the lumbar drain group and 143 in the standard of care group (Figure 1). Lumbar drainage started at a median (IQR) of day 2 (1-2) after aneurysmal subarachnoid hemorrhage. Baseline characteristics showed a higher number of World Federations of Neurosurgical Societies grades 1 and 2 among patients in the lumbar drain group, while patients in the standard of care group had less often intracranial and intraventricular hemorrhages (Table 1). Adjusted analysis takes care of these imbalances occurring at random.

**Table 1.** Baseline Characteristics of Patients Recruited for the EARLYDRAIN trial (Intention-To-Treat Data)

Characteristic	No. (%)	
	Lumbar drain (n = 144)	Standard of care (n = 143)
Age, median (IQR), y	54 (48-63)	56 (48-65)
Sex		
Female	98 (68.1)	99 (69.2)
Male	46 (31.9)	44 (30.8)
Modified Rankin Scale score on admission		
0	136 (94.4)	133 (93)
1	8 (5.6)	10 (7)
Hunt-Hess classification^a^		
1	29 (20.1)	25 (17.5)
2	41 (28.5)	28 (19.6)
3	25 (17.4)	34 (23.8)
4	20 (13.9)	24 (16.8)
5	29 (20.1)	32 (22.4)
WFNS classification^a^		
1	53 (36.8)	42 (29.4)
2	22 (15.3)	21 (14.7)
3	7 (4.9)	10 (7)
4	14 (9.7)	15 (10.5)
5	48 (33.3)	55 (38.5)
Modified Fisher classification^b^		
1	7 (4.9)	3 (2.1)
2	5 (3.5)	7 (4.9)
3	47 (32.6)	54 (37.8)
4	85 (59)	79 (55.2)
Intracerebral hemorrhage	56 (38.9)	50 (35)
Intraventricular hemorrhage	90 (62.5)	85 (59.4)
Aneurysm localization		
ACA	14 (9.7)	11 (7.7)
ACoA	49 (34)	42 (29.4)
ICA	9 (6.2)	15 (10.5)
MCA	25 (17.4)	35 (24.5)
PCoA	23 (16)	20 (14)
BA	10 (6.9)	10 (7)
VA/cerebellar	14 (9.7)	10 (7)
Aneurysms, median (IQR)	1 (1-2)	1 (1-1)
Size of aneurysm, median (IQR), mm^c^	6 (4-8)	6 (5-8)
Aneurysm circulation		
Anterior	120 (83.3)	123 (86)
Posterior	24 (16.7)	20 (14)

Abbreviations: ACA, anterior cerebral artery; ACoA, anterior communicating artery; BA, basilar artery; ICA, internal carotid artery; MCA, middle cerebral artery; PCoA, posterior communicating artery; VA, vertebral artery; WFNS, World Federations of Neurosurgical Societies.

^a^ Hunt-Hess and WFNS (World Federations of Neurosurgical Societies) scales are severity gradings scales, with 1 indicating the least severe and 5 indicating the worst neurological status on admission.

^b^ The modified Fisher classification is a radiological grading scale of subarachnoid hemorrhage severity ranging from 1 to 4, with higher scores indicating more severity.

^c^ Aneurysm size not available in 8 patients.

In the lumbar drain group, 109 patients (75.7%) received a lumbar drain after aneurysm treatment with drainage as specified. A total of 141 patients (98.6%) in the standard of care group were treated according to protocol; 2 (1.4%) received high-volume lumbar drainage as specified for the lumbar drain group. All crossover patients were kept in the intention-to-treat analysis in their randomized groups, independent of actual treatment. This results in a conservative estimation of effect size. Patients from both groups with drainage as specified were looked at in the per-protocol sensitivity analysis, while actual treatment was investigated in the as-treated analysis, with a threshold of 480-mL lumbar drainage in the first 8 days to separate between groups. Reasons for crossover from both groups, as far as known, can be found in eAppendix 1 in Supplement 2; no pattern was noted for crossover patients (eTables 12 and 13 in Supplement 2).

Patients in the lumbar drain group received a median (IQR) daily lumbar drainage of 108 (92-118) mL in the first 8 days. A total of 102 patients (70.8%) in the lumbar drain group and 110 patients (76.9%) in the standard of care group had an external ventricular drain installed. Median (IQR) daily fluid drainage via ventricular drain was lower in the lumbar drain group (98 [60-150] mL vs 171 [110-225] mL; *P* < .001; eTable 3 in Supplement 2).

Patients in the lumbar drain group showed a lower ICP. No differences were noted in mean daily arterial pressure, fever burden, transcranial Doppler, hemoglobin levels, fluid intake, and fluid balance (eFigures 3-13 in Supplement 2).

No differences between the lumbar drain group and standard of care group were observed in the frequency of vasospasm diagnosed clinically (41 [28.5%] vs 48 [33.6%]; *P* = .35), via transcranial Doppler (36 [26.9%] vs 31 [24.8%]; *P* = .70), or with angiography (52 [46.0%] vs 48 [44.0%]; *P* = .77) (Table 2). Ten patients (6.9%) in the lumbar drain group and 14 patients (9.8%) in the standard of care group were treated with either balloon angioplasty or intra-arterial vasodilators as rescue therapy for vasospasm (eTable 3 in Supplement 2).

**Table 2.** Analysis of Primary and Secondary Outcomes

Outcome	No./total No. (%)		Absolute difference (95% CI)	Relative risk (95% CI)	*P* value
	Lumbar drain	Standard of care			
Primary end point					
mRS score of 3-6 at 6 mo	47/144 (32.6)	64/143 (44.8)	−0.12 (−0.23 to −0.01)	0.73 (0.51 to 0.98)	.04
Severity-adjusted relative risk of mRS score of 3-6 at 6 mo^a^	NA	NA	NA	0.76 (0.54 to 1.00)	.047
Secondary outcomes					
Infarct at discharge	41/144 (28.5)	57/143 (39.9)	−0.11 (−0.22 to 0)	0.71 (0.49 to 0.99)	.04
Vasospasm assessment					
Clinically suspected vasospasm	41/144 (28.5)	48/143 (33.6)	−0.05 (−0.16 to 0.06)	0.85 (0.58 to 1.18)	.35
Transcranial Doppler vasospasm^b^	36/134 (26.9)	31/125 (24.8)	0.02 (−0.09 to 0.13)	1.08 (0.70 to 1.58)	.70
Angiographic vasospasm^c^	52/113 (46.0)	48/109 (44.0)	0.02 (−0.11 to 0.15)	1.04 (0.76 to 1.34)	.77
Suspected infection of any cause	56/144 (38.9)	52/143 (36.4)	0.03 (−0.09 to 0.14)	1.07 (0.78 to 1.39)	.66
VP shunt during acute care	34/144 (23.6)	34/143 (23.8)	0 (−0.10 to 0.10)	0.99 (0.64 to 1.46)	.97
Mortality at discharge	15/144 (10.4)	22/143 (15.4)	−0.05 (−0.13 to 0.03)	0.68 (0.35 to 1.23)	.21
mRS score 3-6 at discharge	88/144 (61.1)	101/143 (70.6)	−0.10 (−0.20 to 0.01)	0.87 (0.69 to 1.02)	.09
GOS-E grade of 1-4 at discharge	81/144 (56.2)	90/143 (62.9)	−0.07 (−0.18 to 0.05)	0.89 (0.71 to 1.07)	.25
Barthel Index ≤80 at discharge^d^	73/129 (56.6)	71/121 (58.7)	−0.02 (−0.14 to 0.10)	0.96 (0.75 to 1.16)	.74
VP shunt at 6 mo	41/144 (28.5)	42/143 (29.4)	−0.01 (−0.11 to 0.10)	0.97 (0.66 to 1.36)	.87
Mortality at 6 mo	19/144 (13.2)	25/143 (17.5)	−0.04 (−0.13 to 0.04)	0.75 (0.42 to 1.28)	.31
GOS-E grade of 1-4 at 6 mo	38/144 (26.4)	54/143 (37.8)	−0.11 (−0.22 to −0.01)	0.7 (0.47 to 0.98)	.04
Barthel Index ≤80 at 6 mo^d^	20/125 (16)	35/116 (30.2)	−0.14 (−0.25 to −0.04)	0.53 (0.30 to 0.86)	.01
Sensitivity analysis					
Per protocol					
Infarct at discharge	26/109 (23.9)	56/141 (39.7)	−0.16 (−0.27 to −0.04)	0.60 (0.38 to 0.88)	.009
mRS score of 3-6 at 6 mo	31/109 (28.4)	62/141 (44)	−0.16 (−0.27 to −0.04)	0.65 (0.43 to 0.92)	.01
Severity-adjusted relative risk of mRS score of 3-6 at 6 mo^a^	NA	NA	NA	0.71 (0.48 to 0.97)	.03
As treated					
Infarct at discharge	27/111 (24.3)	71/176 (40.3)	−0.16 (−0.27 to −0.05)	0.60 (0.39 to 0.87)	.006
mRS score of 3-6 at 6 mo	33/111 (29.7)	78/176 (44.3)	−0.15 (−0.26 to −0.03)	0.67 (0.46 to 0.93)	.01
Severity-adjusted relative risk of mRS score of 3-6 at 6 mo^a^	NA	NA	NA	0.73 (0.50 to 0.99)	.04

Abbreviations: GOS-E, Glasgow Outcome Scale–Extended; mRS, modified Rankin Scale score; NA, not applicable; VP, ventriculoperitoneal.

^a^ Adjustment for baseline imbalances performed with the parameters of age, Hunt-Hess grade greater than 3, and intracerebral or intraventricular hemorrhage.

^b^ No transcranial Doppler was performed in 28 patients.

^c^ No angiography was performed after aneurysm occlusion in 65 patients due to early death or local standard operating procedure.

^d^ Analysis performed for surviving patients. Barthel Index at 6 months value missing in 2 patients.

### Primary End Point

**Figure 2.**
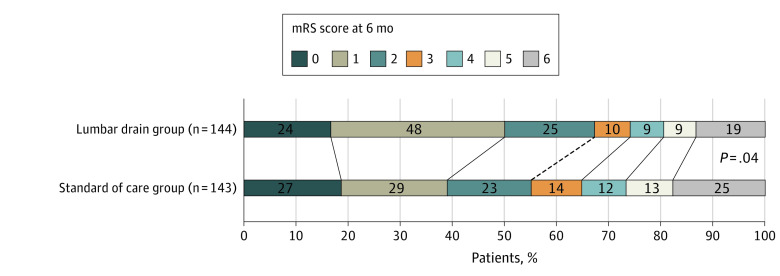
Scores on the Modified Rankin Scale (mRS) at 6 Months, Intention-to-Treat Data Patients in the lumbar drain group received standard of care and additional lumbar drain at a planned rate of 5-mL lumbar cerebrospinal fluid diversion in the first 8 days. Patients in the standard of care group received standard of care subarachnoid hemorrhage treatment alone. Scores on the mRS range from 0 to 6, with 0 indicating no symptoms; 1, no clinically significant disability; 2, minor functional impairment; 3, moderate disability with preserved ability to walk; 4, moderate severe functional impairment without ability to walk without assistance; 5, severe functional impairment requiring constant care; and 6, death.

No patient was lost for evaluation of the primary end point. In the intention-to-treat analysis, 47 of 144 patients (32.6%) in the lumbar drain group and 64 of 143 patients (44.8%) in the standard of care group had an unfavorable outcome at 6 months (unadjusted relative risk, 0.73; 95% CI, 0.51-0.98; absolute risk difference, −0.12; 95% CI, 0.23 to 0.01; *P* = .04) (Figure 2). This equals a number needed to treat of 8.3 for the use of lumbar drains in patients with aneurysmal subarachnoid hemorrhage to prevent a single unfavorable outcome. After adjustment for age, Hunt-Hess grade, and presence of intracerebral and intraventricular hemorrhage, the relative risk was 0.76 (95% CI, 0.54-1; *P* = .047) (eTable 10 in Supplement 2).

### Secondary End Points

A total of 41 patients (28.5%) in the lumbar drain group and 57 patients (39.9%) in the standard of care group experienced a secondary infarction seen on the last cerebral imaging scan before discharge (unadjusted relative risk, 0.71; 95% CI, 0.49-0.99; absolute risk difference, −0.11; 95% CI, −0.22 to 0; *P* = .04). Nineteen patients (13.2%) in the lumbar drain group and 25 patients (17.5%) in the standard of care group died within 6 months (unadjusted relative risk, 0.75; 95% CI, 0.42-1.28; absolute risk difference, −0.05; 95% CI, −0.13 to 0.04; *P* = .31) (eFigure 14 in Supplement 2). There were no differences in causes of death between groups (eTable 11 in Supplement 2). No patient died due to complications related to the lumbar drain. Rates and places of discharge were similar in both groups (eTable 3 in Supplement 2). No difference was seen for the rate of permanent ventriculoperitoneal shunt procedures (Table 2).

### Subgroup Analysis

**Figure 3.**
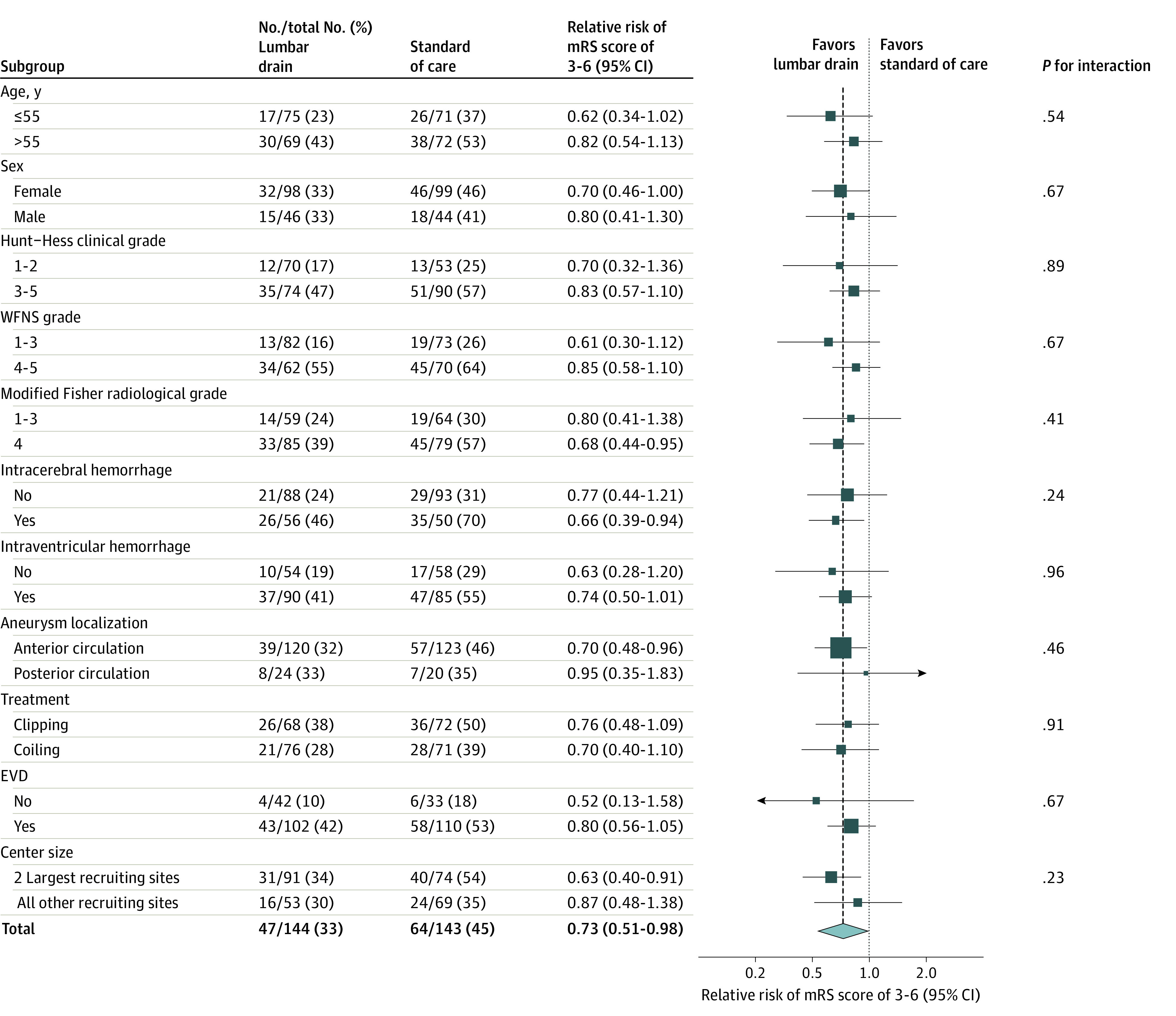
Subgroup Analysis The forest plot shows that the relative risk of moderate-grade to high-grade functional disability or death favors treatment with a lumbar drain additional to standard of care across all prespecified subgroups. Hunt-Hess and World Federations of Neurosurgical Societies (WFNS) scales are severity gradings scales, with 1 indicating the least severe and 5 indicating the worst neurological status on admission. The modified Fisher classification is a radiological grading scale of subarachnoid hemorrhage severity ranging from 1 to 4, with higher scores indicating more severity. EVD indicates external ventricular drain; mRS, modified Rankin Scale.

The effect of lumbar drains on the primary outcome was consistent across prespecified subgroups (Figure 3; eFigures 15-34 in Supplement 2).

### Per-Protocol and As-Treated Sensitivity Analysis

End points for sensitivity analysis were the rate of infarctions at discharge and long-term unfavorable outcome. Analyses were performed unadjusted and adjusted for baseline imbalances in age, Hunt-Hess grade, and intraparenchymal and intraventricular hemorrhages. All outcomes in sensitivity analyses were in favor of the lumbar drain group (Table 2; eAppendixes 2 and 3, eTables 14-19, and eFigures 35-38 in Supplement 2).

### Adverse Events

One patient was reported to develop an increasing gradient of more than 5 mm Hg in ICP readings from the external ventricular drain and the lumbar drain, prohibiting continuation of lumbar drainage. One patient developed a local skin infection at the entrance of the lumbar drain requiring surgical excision. In 1 patient, the lumbar drain was torn off, requiring surgical removal. No difference was noted for the rate of suspected infections (Table 2). In multivariate analysis, the presence of an external ventricular drain was the only risk factor associated with the development of infection (eAppendix 4 and eTables 20 and 21 in Supplement 2).

### Post Hoc Analysis

Vasospasm, detected by clinical means, transcranial Doppler, or with angiography, was associated with the rate of infarctions at discharge. Infarctions at discharge were associated with unfavorable outcome at 6 months (eTables 4-9 in Supplement 2).

## Discussion

In the EARLYDRAIN trial involving patients with aneurysmal subarachnoid hemorrhage of all grades, the use of a lumbar drain in addition to standard of care resulted in less infarctions at discharge and decreased the rate of unfavorable outcome at 6 months. The amount of cerebrospinal fluid drained in the first week was similar in the lumbar drain and standard of care groups. The color difference in fluid from a ventricular and a lumbar drain when both are used simultaneously is visually striking (eFigure 1 in Supplement 2). In subarachnoid hemorrhage, the blood is predominantly in the basal cisterns and the ventricular system. Erythrocytes in cerebrospinal fluid tend to sediment by weight, rendering their removal by a lumbar drain more feasible than by an external ventricular drain.

Patients in the lumbar drain group were noted to have significantly lower ICP (eFigures 4 and 5 in Supplement 2). Approximately 80% of patients with subarachnoid hemorrhage exhibit intracranial hypertension above 20 mm Hg at least once,^25^ and unfavorable outcome is linked to duration and magnitude of ICP elevation.^26^ ICP spikes may trigger spreading depolarizations, which are precursors of impending infarction.^27,28^ Cerebrospinal fluid drainage is an established means to treat hydrocephalus and high ICP. EARLYDRAIN trial data indicate that the way of drainage matters, and lumbar drains are more efficient in attenuating ICP.

Vasospasm, regardless of its definition, was associated with the development of secondary infarctions. The lumbar drain and standard of care groups differed in the rate of secondary infarctions but showed similar vasospasm frequency and severity. This suggests additional mechanisms being necessary for the development of secondary infarctions beyond vasospasm of the large brain supplying vessels. Microcirculation disturbances are a factor difficult to assess at bedside. Techniques like local brain tissue oxygenation monitoring, detection of cortical spreading depolarizations, or continuous surface electroencephalography recording may provide further insight but were too infrequently used in the EARLYDRAIN trial for a sophisticated explanation.

Assumed contraindications to a lumbar drainage are obstructive hydrocephalus and compressed basal cisterns. However, both lack a definition commonly agreed on. Data from traumatic brain injury show that cautious lumbar drainage may be used for treatment of refractory ICP.^29^ Safety concerns based on clinical and imaging judgment only led to crossover in several patients from the lumbar drain group to the standard of care group. We proposed to monitor ICP simultaneously on lumbar and ventricular drains. The lack of a gradient between both pressures indicates open cerebrospinal fluid pathways and safety of lumbar drainage.^18^

The rate of infections in the EARLYDRAIN trail reflects a mixed-grade aneurysmal subarachnoid hemorrhage population. More than half of affected patients develop fever, and roughly 20% develop pneumonia.^30^ Our definition of infection included but was not specific for device-associated meningitis. This would have required frequent cerebrospinal fluid analysis, which itself may trigger infections. Furthermore, requirement of direct pathogen confirmation in cerebrospinal fluid is likely to underrate the problem, and to our knowledge, no laboratory test with sufficient diagnostic value for a device-associated infection exists.^31^

Infarct detection at discharge was mainly performed using CT. Magnetic resonance imaging is likely to be a more sensitive measure but was not routinely used in the EARLYDRAIN trial. We did not use the current composite definition for delayed cerebral ischemia,^32^ with either clinical decline or infarction on imaging or both. Instead, we preferred neurological worsening judged by the clinician in charge and an imaging method separately. Of note, the rate of infarctions was higher than in other contemporary works.^4,5,33^ Key difference to other studies is the inclusion of patients with poor grades (Hunt-Hess grade 5 or World Federations of Neurosurgical Societies grade 5 in the EARLYDRAIN trial), which were excluded elsewhere.

The EARLYDRAIN trial was a randomized trial planned to closely reflect clinical routine. At the trial planning stage, the investigators had no agreement which specific group of patients would benefit from lumbar drainage with negligible risks. While cautious centers proposed to include only patients with good grades for safety reasons, others considered a lumbar drain as mandatory to ease surgery, especially in patients with poor grades with brain edema. Consequently, disease severity varied between centers, and patient inclusions reflect clinical equipoise of the local investigators. Therefore, despite that more than half of the patients were recruited in 2 centers only, we think the results of the EARLYDRAIN trial are generalizable for treatment after aneurysmal subarachnoid hemorrhage.

### Limitations

This trial has several limitations. First, we were unable to secure sufficient funding to allow timely completion of the EARLYDRAIN trial, prohibiting hiring of dedicated personnel.

A significant number of patients in the lumbar drain group did not receive the allocated intervention for various reasons. Crossover patients did not reveal a particular pattern, and sensitivity analysis supported the findings from the intention-to-treat analysis. Patients, relatives, and acute care clinicians were not blinded to the intervention. Although this may be a source of bias, blinded outcome assessment of radiological imaging and clinical status at 6 months minimized this potential.

We did not collect data on preexisting hypertension and other premorbid prognostic factors. We have no detailed information on the thickness of clots or the amount of intraparenchymal and intraventricular blood in the initial CT scan. We did not record medical complications during the clinical course, which happen frequently in patients with subarachnoid hemorrhage.^30^

The EARLYDRAIN trial did not investigate the additional application of clot thrombolysis or irrigation of the subarachnoid space. Also, we are unable to evaluate the benefit of possible higher drainage rates than the suggested 5 mL per hour, although some patients did have a higher drainage amount via lumbar route. This points to possible directions for future research.

## Conclusion

In patients with aneurysmal subarachnoid hemorrhage, prophylactic lumbar cerebrospinal fluid drainage is warranted to lessen the burden of infarction at discharge and reduce the rate of unfavorable outcome at 6 months. Our findings support the use of a lumbar drain additional to standard of care.

## Notes

Supplement 1. Trial Protocol

Supplement 2. **eAppendix 1.** Crossover Patients **eAppendix 2.** Per-Protocol Analysis **eAppendix 3.** As-Treated Analysis **eAppendix 4.** Adverse Events **eTable 1.** Inclusion and Exclusion Criteria **eTable 2.** Reasons Provided for Missed Recruitments **eTable 3.** Clinical Data, Intention-to-Treat Analysis **eTable 4.** Univariate Analysis of Clinical Risk Factors for Secondary Infarctions **eTable 5.** Clinical and Radiological Risk Factors for Development of Secondary Infarctions, Stratified Per Factor Level **eTable 6.** Vasospasm Assessment According to Clinical Estimation and Infarction at Discharge **eTable 7.** Vasospasm Assessment by TCD and Infarctions at Discharge **eTable 8.** Angiographic Vasospasm and Infarctions at Discharge **eTable 9.** Infarctions at Discharge and mRS Score at 6 Months **eTable 10.** Univariate Analysis of Factors Considered for Outcome Adjustment in Intention-to-Treat Data **eTable 11.** Causes of Death in the Lumbar Drain Group and in the Standard of Care Group **eTable 12.** Baseline Characteristics of Crossover Patients Compared With Patients Treated Per Protocol **eTable 13.** Clinical Treatment Data in Crossover Patients **eTable 14.** Demographic Comparison Between Groups in Patients Treated Per Protocol **eTable 15.** Clinical Treatment Data in Patients Treated Per Protocol **eTable 16.** Univariate Analysis of Factors Considered for Outcome Adjustment in the Per-Protocol Data **eTable 17.** Demographic Comparison Between Groups As Actually Treated **eTable 18.** Clinical Treatment Data Between Groups As Actually Treated **eTable 19.** Univariate Analysis of Factors Considered for Outcome Adjustment in the As-Treated Data **eTable 20.** Univariate Analysis of Potential Factors for the Development of Infection in the Clinical Course **eTable 21.** Multivariate Analysis of Factors Associated With the Development of Infection **eFigure 1.** Drip Chambers of an EVD and a Lumbar Drain **eFigure 2.** Time Flow of Randomizations **eFigure 3.** Daily Mean Arterial Pressure **eFigure 4.** ICP at 7 AM **eFigure 5.** Highest ICP on Each Day **eFigure 6.** TCD Values **eFigure 7.** Fluid Intake Per Day **eFigure 8.** Net Fluid Balance Per Day **eFigure 9.** Comparison of Fever Burden **eFigure 10.** Lowest Hemoglobin Value Per Day **eFigure 11.** Drainage via EVD **eFigure 12.** Drainage via Lumbar Drain **eFigure 13.** Cerebrospinal Fluid Drainage of External Ventricular and Lumbar Drains Combined **eFigure 14.** Mortality in the Intention-to-Treat Data **eFigure 15.** Outcome in Patients Younger Than and Up to Median Age of 55 Years **eFigure 16.** Outcome in Patients Older Than Median Age of 55 Years **eFigure 17.** Outcome in Female Patients **eFigure 18.** Outcome in Male Patients **eFigure 19.** Outcome in Good-Grade Patients (Hunt-Hess Grades 1 and 2) **eFigure 20.** Outcome in Poor-Grade Patients (Hunt-Hess Grade 3-5) **eFigure 21.** Outcome in Good-Grade Patients (WFNS Grades 1-3) **eFigure 22.** Outcome in Poor-Grade Patients (WFNS Grades 4 and 5) **eFigure 23.** Outcome in Patients Presenting Without Intracerebral Hemorrhage **eFigure 24.** Outcome in Patients With Intracerebral Hemorrhage **eFigure 25.** Outcome in Patients Without Intraventricular Hemorrhage **eFigure 26.** Outcome in Patients With Intraventricular Hemorrhage **eFigure 27.** Outcome in Patients With an Aneurysm of the Anterior Circulation **eFigure 28.** Outcome in Patients With Posterior Circulation Aneurysms **eFigure 29.** Outcome in Patients Treated With Clipping **eFigure 30.** Outcome in Patients Treated With Coiling **eFigure 31.** Outcome of Patients Treated Without an External Ventricular Drain **eFigure 32.** Outcome in Patients Where an External Ventricular Drain was Required **eFigure 33.** Outcome in Patients Treated at the 2 Largest Recruiting Centers **eFigure 34.** Outcome in All Other Than the 2 Largest Recruiting Centers **eFigure 35.** CONSORT Diagram for Per-Protocol Analysis **eFigure 36.** Mortality Analyzed Per Protocol **eFigure 37.** CONSORT Diagram for As-Treated Analysis **eFigure 38.** Mortality According to Actual Treatment

Supplement 3. Group Information. EARLYDRAIN Study Group

Supplement 4. Data Sharing Statement
