# Supplementary material for: Effectiveness of Lumbar Cerebrospinal Fluid Drain Among Patients With Aneurysmal Subarachnoid Hemorrhage: A Randomized Clinical Trial
Source: JAMA Neurol. 2023 Jun 18;80(8):833–42. doi: 10.1001/jamaneurol.2023.1792 (PMC10277935; doi:10.1001/jamaneurol.2023.1792)
Supplement: Supplement 1. — Trial Protocol [file jamaneurol-e231792-s001.pdf]

Supplement 1 – Earlydrain Trial Protocol

Supplement to:

Stefan Wolf, Dorothee Mielke, Christoph Barner, et al, for the Earlydrain Study Group:
Lumbar Drains and Functional Outcomes in Patients with Aneurysmal Subarachnoid Hemorrhage: a Pragmatic Multicenter Randomized Trial

This supplement contains the following items:

Final Protocol, published September 14th, 2011	3
First Protocol Version, dated December 9th, 2010	15
Summary of changes between protocol versions	32
Statistical analysis plan	33
Case report form (English Version)	35

EARLYDRAIN- outcome after early lumbar CSF-drainage in aneurysmal subarachnoid hemorrhage: study protocol for a randomized controlled trial

This is the final version of the protocol which all Earlydrain investigators agreed upon. It was developed by the lead investigators of the two primary study centers, Erlangen and Berlin.

The protocol was subsequently published in TRIALS (Bardutzky et al, Trials 2011, 12:203) and served as introduction for all centers on the Earlydrain trial and its objectives.

Abstract

Background: Aneurysmal subarachnoid hemorrhage (SAH) may be complicated by delayed cerebral ischemia, which is a major cause of unfavorable clinical outcome and death in SAH-patients. Delayed cerebral ischemia is presumably related to the development of vasospasm triggered by the presence of blood in the basal cisterns. To date, oral application of the calcium antagonist nimodipine is the only prophylactic treatment for vasospasm recognized under international guidelines.

In retrospective trials lumbar drainage of cerebrospinal fluid has been shown to be a safe and feasible measure to remove the blood from the basal cisterns and decrease the incidence of delayed cerebral ischemia and vasospasm in the respective study populations. However, the efficacy of lumbar drainage has not been evaluated prospectively in a randomized controlled trial yet.

Methods/Design: This is a protocol for a 2-arm randomized controlled trial to compare an intervention group receiving early continuous lumbar CSF-drainage and standard neurointensive care to a control group receiving standard neurointensive care only. Adults suffering from a first aneurysmal subarachnoid hemorrhage whose aneurysm has been secured by means of coiling or clipping are eligible for trial participation. The effect of early CSF drainage (starting < 72 h after securing the aneurysm) will be measured in the following ways: the primary endpoint will be disability after 6 months, assessed by a blinded investigator during a personal visit or standardized telephone interview using the modified Rankin Scale. Secondary endpoints include mortality after 6 months, angiographic vasospasm, transcranial Doppler sonography (TCD) mean flow velocity in both middle cerebral arteries and rate of shunt insertion at 6 months after hospital discharge.

Discussion: Here, we present the study design of a multicenter prospective randomized controlled trial to investigate whether early application of a lumbar drainage improves clinical outcome after aneurysmal subarachnoid hemorrhage.

Trial registration: www.clinicaltrials.gov Identifier: NCT01258257

Background

Non-traumatic subarachnoid hemorrhage (SAH) is a major cause of stroke accounting for approximately 1-7% of cases. In 80% of SAH-cases the source of bleeding is a ruptured cerebral aneurysm [1,2]. Important for a patient's prognosis is the severity of the initial bleeding and complications associated with the presence of blood in the subarachnoid space. Once the aneurysmal SAH has occurred patients are predominantly threatened by two distinct problems in the acute phase. First, they may experience a further, often more severe, hemorrhage, and second, they may suffer delayed neurologic deterioration (DND) caused by delayed cerebral ischemia (DCI). The consequences of DCI may either be transient or may result in cerebral infarction with persistent neurologic disability or death.

The first problem, aneurysmal rebleeding, is solved through rapid cerebrovascular imaging and subsequent treatment of the ruptured aneurysm, thus preventing recurrent hemorrhage. Aneurysm treatment may be performed either via craniotomy and surgical clipping of the aneurysm or using endovascular techniques by occluding the aneurysm with small platinum coils.

The second problem, DCI, is more difficult to recognize and to handle. Patients after aneurysmal SAH experience DND with an incidence of 30 to 60% [3]. It may be caused by hydrocephalus, cerebral edema, fevers, seizures, electrolyte abnormalities, and DCI. Strongly associated with DCI / cerebral infarction is a constringency reaction of the vessels supplying the brain with blood, called vasospasm [4]. The pathomechanism leading to vasospastic vessel constriction is not completely understood [5] and the quantitative relevance of vasospasm for the development of DCI is less clear than previously assumed [6].

Clinical signs of DND accompanying radiographic vasospasm are variable, depending on the affected blood vessels including alteration of mental status, aphasia, hemiparesis, or any other focal neurologic deficit. Often the consequences of this condition may include permanent neurologic deficits and death due to infarction and subsequent herniation of the brain. DCI, DND and vasospasm may be causatively interlinked, but also be independently present from each other. Vasospasm may be asymptomatic without clinically apparent deterioration of the patient's condition or external circumstances, such as deep sedation, may prevent clinical detection of a deterioration caused by vasospasm.

As DND is unspecific concerning its etiology, clinical judgment, therefore, is unreliable for the prediction and recognition of vasospasm. Thus digital subtraction angiography is the procedure of choice for the detection of vasospasm. Vasospasm may be present in the proximal vessels, the distal branches of the vasculature, or both.

Currently the only measure recognized for the prevention of DCI is the prophylactic application of the calcium channel blocker nimodipine [7]. Newer approaches, to date not included in official guidelines but pursued in several centers, include medication with statins and magnesium [8].

One hypothesis claims that the likelihood of angiographic vasospasm to occur is related to the amount of blood in the basal cisterns. According to this consideration, one prophylactic strategy is to remove as much of this blood as early as possible. If clipping of the aneurysm is performed this can be achieved intraoperatively by opening the terminal lamina and irrigating the blood from the basal cisterns. Albeit promising, studies addressing the efficacy of this measure show inconclusive results [9]. This approach is not feasible if the aneurysm is secured using an endovascular approach.

Excess removal of cerebral spinal fluid (CSF) via an external ventricular drain fails to prevent vasospasm and may lead to a higher incidence of posthemorrhagic shunt dependency [10,11]. Supposedly this is because after aneurysmal SAH, the blood settles and clots in the basal cisterns and therefore only CSF, being more lightweight, is removed via the ventricular drain.

Application of a lumbar drain has been proposed as an alternative approach to address clotting of the blood in the basal cisterns. In two retrospective studies in patients after aneurysmal SAH, the safety of this approach was shown [12,13]. One of these studies addressed the radiologic and clinical outcome after surgical clipping [12], while the other addressed the outcome after endovascular coiling [13]. Both studies led to a markedly diminished incidence of angiographic vasospasm and improvement in clinical outcome measured by the Glasgow Outcome Scale (GOS). Therefore a prospective study addressing the efficacy of this novel approach is warranted and currently being conducted (EARLYDRAIN).

The aim of the EARLYDRAIN study is to examine the efficacy of application of lumbar drainage in patients with acute subarachnoid hemorrhage from a ruptured cerebral aneurysm. The hypothesis is that early application of a lumbar drain after aneurysmal SAH leads to an improved outcome at six months after the hemorrhage, measured by the modified Rankin score. Furthermore, it is hypothesized that this postulated clinical effect will be due to a diminished incidence of cerebral vasospasm and delayed cerebral ischemia. Therefore the incidence of angiographic vasospasm and the development of new infarctions shown on CCT at discharge of the patient will be among the secondary endpoints of the present study.

Methods

Study design

The present study is in compliance with the Helsinki Declaration. Ethical approval was obtained by the ethical committee of the medical faculty of the Friedrich-Alexander-University Erlangen-Nürnberg (reference number: 4171).

The EARLYDRAIN study is a 2-arm randomized controlled trial to compare an intervention group receiving early continuous lumbar CSF-drainage and standard neurointensive care to a control group receiving standard neurointensive care only. It is conducted by a German national study group consisting of neurosurgical centers treating at least 30 patients with aneurysmal subarachnoid hemorrhage per year. Data management and monitoring will be performed by the Center for Stroke Research Berlin (CSB) at Charité University Medicine, Berlin, Germany.

Patients suffering from an aneurysmal SAH and completed elimination of the causative aneurysm are being recruited for this study. The choice of the method of aneurysm treatment is at the discretion of the neurovascular team taking care of a patient and not specified by the study protocol. All medical treatment is performed according to local guidelines and standard operating procedures.

Subject Inclusion criteria

- Age: 18 years or older
- First aneurysmal SAH
- Pre-morbid modified Rankin Scale score 0 ("no symptoms at all") or 1 ("no significant disability despite symptoms")
- Aneurysm treatment performed during the first 48 hours after the initial hemorrhage.
- Informed consent by the patient or his/her legal representative. In case neither the patient is capable of giving informed consent nor a legal representative is available, informed consent can be given by an independent physician neither involved in the patient's treatment nor in conducting the trial.

Subject Exclusion criteria

- Subarachnoid hemorrhage of other than aneurysmal origin
- No hemorrhage visible on initial CCT-scan (Fisher Grade I)
- Pregnancy
- Concurrent participation in another interventional trial (participation in an observational trial is not an exclusion criteria)
- Life expectancy less than 1 year for other reasons than the current SAH
- Other concomitant severe disease that would confound with treatment
- Other clear contraindication for treatment with a lumbar drain (e.g. absent or compressed basal cisterns on the admission CCT)

Interventions

In order not to provoke premature rupture of the aneurysm due to accidental drainage, randomization to the study and eventual placement of a lumbar drain takes place after securing the aneurysm by the preferred method of choice (Figure 1). Every patient in the lumbar drainage group (LD-group) receives a lumbar drain during anesthesia required for the aneurysm treatment. Insertion of a lumbar drain into the subarachnoid space is conducted in standard fully sterile technique. This is to be performed before anticoagulation or anti-platelet therapy is initiated, which sometimes is warranted after endovascular coiling. A post-procedural CCT scan of the brain is performed within 24 hours after aneurysm treatment. In case of neurological worsening after the procedure it is strongly recommended to perform the follow-up CCT-scan as soon as possible.

In patients in the LD-group, CSF drainage via the lumbar drain is started slowly and steadily at a rate of approximately 5 ml per hour after the post-interventional CCT. This leads to a planned daily CSF-drainage of 120 ml per day through the lumbar route. Patients in both groups may receive additional CSF drainage via a ventricular device. The amount of CSF drained via the ventricular route is determined according to clinical requirement and not specified by the study protocol.

In order to enhance accuracy of the amount of CSF drained, regular drainage control every other hour and stopping in case of excess drainage is strongly recommended by the principal investigators. In case of neurological decline suspiciously related to the lumbar drainage, the drain must be closed immediately. Drainage may be gradually restarted after 12 to 24 hours, after performing a CCT scan.

If the post-procedural CCT or any other follow-up CCT scan shows compressed basal cisterns or any signs of threatening herniation, lumbar CSF diversion in the LD- group must not be performed. It may still be feasible to carefully drain CSF via the lumbar route [14], but this is at the discretion of the local investigator and not recommended. In patients requiring sedation and mechanical ventilation, either due to neurological impairment or for other medical reasons, intracranial pressure monitoring is mandatory. This may be performed according to local policy either with parenchymal or ventricular devices. If the intracranial pressure exceeds 20 mmHg, further CSF drainage via lumbar route shall be interrupted until the ICP is below 20 mmHg again. Careful CSF-drainage via the lumbar route may still be feasible in case of high intracranial pressure [14], but again this is at the discretion of the local investigator. A method of detecting the safety of lumbar CSF diversion may be determining the gradient of lumbar versus intracranial CSF pressure [15]. Albeit promising, the Earlydrain investigators explicitly rate this approach preliminary and experimental. In case of doubt, lumbar CSF diversion must not be performed.

Further neuromonitoring with transcranial duplex sonography (TCD), electroencephalography (EEG), brain tissue oxygenation recordings, jugular bulb oxymetry, regional cerebral blood flow measurement, microdialysis or other methods is at the discretion of the center and according to its local guidelines. As far as possible, this data should be saved electronically for post-hoc analysis.

A CCT scan as well as conventional digital subtraction angiography (DSA), CT angiography or MR angiography for assessment of vasospasm in the larger vessels is routinely performed on day 7 to 10 after the initial hemorrhage, according to local guidelines. In case of the occurrence of a DND when vasospasm is assumed to be the cause, angiography may be performed at any time. If it is performed earlier than day 7 to 10 and the patient shows no clinical deterioration thereafter, the angiography on day 7 to 10 is omitted. Treatment of radiographically confirmed vasospasm is according to local guidelines and not specified in the Earlydrain study protocol. It may include augmentation of cerebral blood flow via hypertensive hypervolemia as well as endovascular balloon dilation or intraarterial infusion of vasodilators.

After cerebrovascular imaging on day 7 to 10 the lum- bar drainage of CSF is stopped in the LD-group. If cerebrovascular imaging is carried out before day 7, lumbar drainage is stopped on day 8. It may be pursued on a clinical base, as required.

Amount and duration of CSF drainage

Patients randomized to the lumbar drainage group shall receive a daily drainage of 120 ml CSF, or 5 ml per hour for seven days. If higher amounts of CSF need to be drained on clinical grounds as in patients with hydrocephalus, this is preferably performed via an external ventricular drain.

The drain is planned to remain in place until the con- trol angiography on day 7 to 10 after the initial hemorrhage. The local investigator may decide to remove the drain earlier in patients fully mobilized without clinical necessity of CSF drainage. However, consecutive drainage should not be less than four days to achieve a valid study result. Lumbar CSF drainage may be prolonged beyond the control

angiography on clinical requirement. The amount of CSF drainage may then be adjusted to clinical requirements and bears no further restriction.

Patients randomized to the control group should not receive a lumbar drain before the planned control angiography to be performed on day 7 to 10 after SAH. If the patient develops hydrocephalus, and no EVD was placed initially for CSF drainage, a lumbar drain may be installed at the discretion of the local investigator. These patients are analyzed in the intention-to-treat analysis, but are not suitable for the per-protocol analysis.

Consent to study participation

Consent to study inclusion is sought after explanation and agreement to a specific aneurysm treatment. Thus, patients capable of consenting to the aneurysm treatment will be informed about the study details themselves and may or may not agree to participate. If a patient is incapable of consenting to the proposed treatment, the legal representative should be informed on the conditions of treatment choices and afterwards, on the details of the EARLYDRAIN study. A patient may be randomized if the legal representative gives informed consent to the study, based on the presumed will of the patient. If neither the patient is capable of giving informed consent nor a legal representative is available in due time, an independent physician not involved in the patient’s treatment nor in the trial may be asked for study approval. The latter option reflects a distinct characteristic of German law and the local ethic committee may or may not permit this.

This option was introduced into the consent procedure because the aforementioned retrospective data on lumbar drainage for treatment of aneurysmal SAH suggests a potentially beneficial effect of the measure for the patient. Therefore it shall not be categorically withheld from patients who are not capable of deciding whether to participate in the study or not and who do not have a legal representative.

However, in these cases of deferred consent, a legal representative needs to be established as soon as possible, according to German law. From our experience this legal procedure requires a time period from proposal to establishment of a legal representative of up to 72 hours, thus requiring the consent of an independent physician upfront for study inclusion. As soon as a legal representative is available and/or the patient is capable again to consent to the study, he or she must be asked to give informed consent. If the patient or his/her legal representative refuses consent after inclusion by advice of an independent physician, the patient’s further study participation is no longer possible. In this case, however, the patient or his/her legal representative is asked to give consent for evaluation of already acquired data.

The detailed explanation of the study to the patient, legal representative or independent physician has to be carried out using appropriate explanations and words depending on the previous medical knowledge of the respective person and her/his level of education. During the explanations the respective person will be asked on a regular basis if she/he understands the conveyed information and if any questions have arisen. In addition to these verbal explanations the patient / legal representative / independent physician will be given a leaflet containing the study details. After reading the leaflet the respective person will be given as much time as she/he demands for the decision on study participation.

Randomization

Any patient meeting the inclusion criteria and not violating the exclusion criteria may participate in the EARLYDRAIN study and be randomized to either receive a lumbar drain or not, thus defining the two distinct groups LD and NoLD.

Randomization is performed via a dedicated internet site accessible for all local investigators of the participating trial centers http://www.randomizer.at. No stratification or minimisation is to be used.

The security measures of the online randomization system "randomizer.at" include that 1. All transactions are logged, 2. The audit trail of the trial can be accessed and analysed any time by the trial monitoring committee. 3. Network traffic between the web-browser and the randomizer is encrypted using SSL (Secure Sockets Layer) with strong encryption.

Sample size calculation

In the ISAT trial, the largest trial on the treatment of aneurysmal subarachnoid hemorrhage so far, the mortality at one year follow-up was 8.1% to 10.1% [16]. Given the data from both retrospective studies on lumbar drains after SAH, a reduction from 15% to 2.1% after coiling and from 5% to 3% after clipping was shown. Thus, both studies were way lower in their mortality rate and, therefore, their external validity may be questioned.

In the two retrospective trials, 167 [12] and 107 [13] patients were studied, respectively. The effect of lumbar drainage was a decrease of the incidence of "clinical vasospasm" by 34% [12] and 40% [13], respectively.

In the above-mentioned studies the term "clinical vasospasm" includes neurological deterioration not explainable by hemorrhage, cerebral edema, hydrocephalus, hyponatremia, drug toxicity, infection or seizures. No distinction is made between delayed cerebral ischemia (DCI) and vasospasm as potential causes of the clinical worsening.

The following statistical calculations are based on the assumption that the clearly defined subtype of "delayed neurological deficit" measured in the above-mentioned two retrospective trials is highly correlated with clinical outcome 6 months after SAH, which constitutes the primary endpoint of the EARLYDRAIN study.

For lack of previous studies assessing clinical outcome after lumbar drainage in SAH as a primary endpoint this assumption seemed justified.

To assess a decrease of the incidence of DND from 40% to 20% in a prospective clinical trial, 93 patients in each of the two study arms are required to gain a power of 85%, using an alpha error of 5%. To account for possible imbalances in the randomization procedure concerning severity of clinical and radiological grading of the SAH or the choice of treatment and to facilitate a preplanned analysis on the severity of the initial hemorrhage, the planned study size is to include and randomize altogether 300 patients. This results in a power of 85.2%, again, using an alpha error of 5%, to detect a decrease in the rate of severe disability on a dichotomized modified Rankin scale from 50% to 33%, which would be consistent with the effect size from the retrospective trials on a dichotomized GOS [12,13]. The EARLYDRAIN investigators are aware of other, more conservative calculations for the sample size of patient-centered outcome studies targeting vasospasm, indicating that there may be the necessity to include more than 5000 patients in a single trial [17]. The power calculations, as described above and based on the available retrospective data, do not substantiate numbers this large. Besides feasibility issues, clinical experience from the principal investigators considering the expected effort-benefit ratio does not warrant enlargement of the trial to detect a rather small difference between groups.

Safety of lumbar drains after aneurysmal SAH

In the above-mentioned two retrospective studies, mortality was lower in the lumbar drainage group. Neither of the retrospective studies mentions procedural related complications for the lumbar drains [12,13]. In patients with increased intracranial pressure, careful lumbar drainage of CSF may be a possible treatment even in case of compressed basal cisterns [14]. A feasible strategy to enhance safety is determination of the lumbar-cranial pressure gradient and cessation of lumbar CSF diversion in patients with increasing pressure difference [15].

However, in patients presenting increased intracranial pressure or compressed basal cisterns on CCT-scan the risk associated with lumbar drainage is unclear according to the current state of medical knowledge. In unclear cases the investigator must refrain from the insertion of a lumbar drain.

Outcome assessment

The primary endpoint is disability after 6 months, assessed by the modified Rankin Scale [18] dichotomized at a score of 0 to 2 versus 3 to 6 (6 = death). Assessment is performed by a blinded investigator of the local study center by personal visit. Alternatively, a telephone questionnaire is suitable for outcome assessment using the modified Rankin Scale [19]. Outcome assessment is planned to be done on the whole dataset as well as in preplanned stratified subsets (i.e. for example clinical SAH grade according to the Hunt & Hess scale 1-2 vs. 3-5 [20], CT grading according to Fisher I-III vs. IV [21]).

Secondary outcome criteria are:

- Mortality after 6 months
- mRS score after 6 months as continuous variable
- Angiographic vasospasm on day 7 to 10, as defined by a caliber reduction by 33% or more compared to the initial digital subtraction angiography
- Endovascular rescue therapy performed due to proven vasospasm, using balloon dilation of spastic vessels and/or arterial infusion of vasodilators
- Infarction (due to vasospasm) in the last CT scan before discharge
- Expression of clinical delayed neurological deficit after the aneurysmal SAH until discharge from acute care.
- Daily course of TCD mean flow velocity in both MCA at a depth of 50-60 mm
- Rate of death during the initial hospital treatment after the aneurysmal SAH.
- Rate of CSF shunt insertion during the first six months
- Presence of CSF infection during the first 14 days, as defined by modified CDC criteria for device-associated meningitis (treatment required on either positive culture, or elevated cell count, red cell/white cell ratio, increased lactate and/or decreased glucose) [22].

The following parameters will be recorded and used in predictor-/association models concerning primary and/ or secondary outcome parameters:

- Gender
- Age
- Hunt&Hess grade on admission
- Time from symptom onset to admission
- Location of aneurysm
- Time from symptom onset to aneurysm treatment
- Treatment of aneurysm by clipping or coiling or both
- Time from symptom onset to randomization
- Time from symptom onset to treatment start (i.e. insertion of the lumbar drainage in the treatment arm)
- Time from admission to discharge
- Insertion of EVD (yes/no)
- Duration of EVD being in place
- Duration of lumbar drainage
- Amount of CSF drained by EVD [ml]
- Amount of CSF drained by lumbar drain [ml]
- Use of nimodipine (yes/no)
- Use of statins (yes/no)
- Use of Mg^{2+} (yes/no)

- Transcranial Doppler ultrasound in both MCA at 50-60 mm depth, 1x daily (> 160 cm/s versus < 160 cm/s)
- Presence of CSF infection during hospital stay (yes/ no)

Data Management and Monitoring Body

All data specified in the trial protocol will be documented in the patient's records and on standardised Case Report Forms (CRFs), available as original with two copies. The investigating physician is responsible for appropriate completion of the form. The (CEHRIS) of the Center for Stroke Research Berlin (CSB) is responsible for data base development, data acquisition via double entry, data storage, and validation. Data validation includes controls of completeness, consistence and plausibility of the data documented in the CRF using a query system between data management and investigating physician. After resolution of all queries concerning enrolled patients, the data bank is closed (end of the trial) and forwarded to the biometrician for the purpose of evaluation. After finalization of all evaluations the final report and all original CRFs are delivered to the principal investigator.

The trial is supervised and monitored by the Intensive Care Treatment of Stroke group (ICTOS) of the CSB including initiation and regular site visits, source data verification, and reports of adverse events. All data management and supervising procedures are performed according to Standard Operation Procedures (SOPs) of the CSB and in accordance to ICH-GCP Guidelines (E6) and the declaration of Helsinki.

Adverse events (AE) and severe adverse events (SAE)

Apart from AE and SAE which may occur after the beginning of the trial (synchronous with the insertion of the LD) there are complications related to securing the aneurysm:

Surgery-related complications: Surgical treatment includes the known risks of surgical interventions.

Complications related to endovascular therapy: Endovascular therapy includes the risks known to be associated with it.

Definition of adverse events and severe adverse events

The term "adverse event" (AE) describes any sign, symptom, syndrome or any disease 1. occurring newly in a trial participant after consent to the trial and 2. being of particular interest for the assessment of the disease or the security of the therapeutic concept. In this trial AEs include:

- Arterial or venous thrombosis,
- Complications related to insertion of a lumbar drainage,
- Any SAE

The term AE does not implicate a causal correlation with the participation in the trial. Surgical or endovascular interventions are not necessarily considered as AE but can be necessary for the therapy of an AE. AEs are divided in severe (SAE) and non-severe (AE) adverse events.

An SAE is any AE occurring during the trial that is related to:

- Death
- Any life-threatening condition
- Re-hospitalisation or prolongation of hospitalization
- Long-term or severe restraint of the state of health, or
- Birth deformities.

Documentation

Investigation of AEs is part of every assessment of the study participants. Any AE has to be documented in the CRF.

Every SAE has to be documented on a special documentation form and has to be reported within 24 hours after recording, but at least at the next working day, to the data monitoring center in Berlin.

Statistical Analysis

All data are described according to their mean, median or frequency, as applicable. The dichotomized modified Rankin score as primary outcome variable is investigated in using univariate analysis. Multivariate logistic regression modeling is performed accordingly, adjusted for clinical grade, Fisher grade, ventricular hemorrhage, parenchymal hemorrhage, gender, nimodipine or other concomitant medical treatment. Analysis is planned as intention-to- treat as well as per protocol, excluding the patients who were treated with amounts of CSF drainage via lumbar drain deviating from the specified 5 ml/h or which needed a lumbar drain when randomized to the No-LD group.

Interim Analysis

An interim analysis after inclusion of 150 patients will address safety issues. This analysis focuses on the secondary endpoints and SAEs only, especially the rate of death during hospital stay. During the interim analysis, the recruitment for the EARLYDRAIN study is not stopped. The frequency of further safety analyses will be adjusted to the recommendations of the data and safety monitoring board (DSMB).

Data and Safety Monitoring Board (DSMB)

Safety aspects of the trial are supervised by the DSMB. The DSMB consists of an independent stroke physician, a neurosurgeon, and a neurointensivist, neither involved in the planning nor conduction of the trial nor participating in the trial. The DSMB independently elects a chairman. The DSMB is responsible for critical evaluation and suggestions for improvement of the trial protocol and supervision of the trial course. The DSMB has to be informed about the results of safety issues, especially the number of AEs and SAEs in each treatment group at least after every 10 patients having been enrolled, but at least every 6 months starting with the day of inclusion of the first patient, but also whenever the Steering Committee believes this to be necessary. Based on the results of safety aspects the DSMB will recommend to continue or stop the trial. The members of the DMSB confer personally or via telephone and report their recommendations to the Steering Committee.

Steering Committee

The steering committee consists of the neurosurgical (Stefan Wolf, principal investigator) and the neurological (Jürgen Bardutzky) project manager along with the directors of the two leading trial centers (Stefan Schwab and Peter Vajkoczy). The Steering Committee is responsible for planning of the trial including funding, development of the trial protocol in cooperation with the participating centers, design of patient's and legal representative's information and informed consent, approval of the trial protocol and informed consent including later amendments by legal authorities and ethics committees, selection, verification, and recruitment of potential trial centers, design of the CRF, organisation of a randomization system on a 24-hours/7-days basis including a trial-phone hotline. Based on the recommendations of the Data Safety and Monitoring Board (DSMB) the Steering Committee decides on preliminary termination of the trial. The Steering Committee can also stop the trial preliminarily, if advised so for other reasons by the DSMB. Furthermore the Steering Committee has to give consent to reports and publication of trial results.

Discussion

Here we describe the design of a multi-center prospective randomized controlled trial to investigate whether early lumbar drainage improves clinical outcome after aneurysmal subarachnoid hemorrhage.

If one assumes that the primary hemorrhage usually occurring outside of the hospital is difficult to prevent because carriers of aneurysms are usually asymptomatic, then - apart from elimination of the aneurysm itself - delayed cerebral ischemia due to radiographically detectable vasospasm constitutes the most important aspect of aneurysmal SAH that causes substantial morbidity and mortality. The pathophysiological mechanisms underlying either of these entities and possibly influencing each other have not been understood sufficiently. Arterial narrowing as seen on angiography may be highly correlated with unfavourable clinical outcome but it is assumed that outcome-defining factors are more diverse [6]. To account for the complexity of factors causing early clinical deterioration after aneurysmal SAH the rather abstract term “delayed neurological deficit” has been created. However not only the interaction between these factors but also their influence on clinical long-term outcome remains speculative.

The present study is based on the belief that in the majority of cases DND is caused by angiographically detectable arterial narrowing and that the occurrence of DND, including DCI, and radiographic vasospasm are major factors for unfavorable outcome.

Since the development of nimodipine as a prophylactic agent against DND and DCI no new treatment strategies have been included in international guidelines. This emphasizes the necessity of new ways to approach pro- phylaxis and therapy of DCI.

The hypothesis that lumbar drainage may improve out- come after SAH was derived from the results of two previous retrospective investigations that have suggested a beneficial effect concerning the development of clinical deterioration [12,13]. However, the aforementioned studies used development of “clinical vasospasm” as primary endpoint whereas in the EARLYDRAIN study the primary endpoint is degree of disability after 6 months assessed in a prospective blinded manner using the mRS- score. Thus EARLYDRAIN focuses on clinical outcome, allowing direct conclusions about the benefit of early lumbar drainage in patients having experienced an aneurysmal SAH. Furthermore by choosing clinical outcome as the primary endpoint the authors of the present study tried to avoid ambiguity due to heterogenous believes concerning the etiology of DND and the role of radiographic vasospasm as an outcome-influencing factor or a mere epiphenomenon, respectively.

The focus on clinical outcome is also a feature that clearly distinguishes the EARLYDRAIN study from the LUMAS trial ("Lumbar drainage after subarachnoid hemorrhage”, NCT00842049). This study has been completed in February 2011 and its results are awaited.

The LUMAS trial is a Phase II randomized clinical trial, the primary endpoint is the incidence of delayed ischemic neurologic deficits within three weeks after the initial hemorrhage. Clinical outcome according to the modified Rankin Scale score at 10 days and 6 months after the ictus are among the secondary outcome measures. The focus of LUMAS are efficacy of lumbar drainage after aneurysmal SAH with respect to the primary endpoint. The results of this trial will be studied carefully by the EARLYDRAIN investigators with regard to efficacy and safety of the employed methods. Because of reverse, but comparable primary and secondary endpoints of the two studies their results offer the opportunity of a combined analysis.

List of abbreviations

AE: Adverse event; SAE: Severe adverse events; SAH: Subarachnoid hemorrhage; LD: Lumbar drainage; No-LD: No lumbar drainage; CSF: cerebrospinal fluid; CCT: cranial computed tomography; MRI: Magnetic resonance imaging; TCD: transcranial duplex sonography; DSA: digital subtraction angiography; CRF: case report form; mRS: Modified Rankin scale; GOS: Glasgow outcome scale;

EVD: External ventricular drain; DND: Delayed neurological deficit; DCI: Delayed cerebral ischemia; MCA: Middle cerebral artery; CSB: Center for stroke research Berlin; ICTOS: Intensive care treatment of stroke study group/Charité Berlin; DSMB: Data and safety monitoring board; LUMAS: “Lumbar drainage after subarachnoid hemorrhage"-study.

References

1. van Gijn J, Rinkel GJ: Subarachnoid haemorrhage: diagnosis, causes and management. Brain 2001, 124:249-78.
2. Stehbens WE: Aneurysms and Anatomical Variation of Cerebral Arteries. Arch Pathol 1963, 75:45-64.
3. Keyrouz SG, Diringer MN: Clinical review: Prevention and therapy of vasospasm in subarachnoid hemorrhage. Crit Care 2007, 11:220.
4. Crowley RW, Medel R, Dumont AS, et al: Angiographic vasospasm is strongly correlated with cerebral infarction after subarachnoid hemorrhage. Stroke 2011, 42:919-23.
5. Pluta RM, Hansen-Schwartz J, Dreier J, et al: Cerebral vasospasm following subarachnoid hemorrhage: time for a new world of thought. Neurol Res 2009, 31:151-8.
6. Diringer MN, Bleck TP, Claude Hemphill J, et al: Critical Care Management of Patients Following Aneurysmal Subarachnoid Hemorrhage: Recommendations from the Neurocritical Care Society’s Multidisciplinary Consensus Conference. Neurocrit Care 2011.
7. Allen GS, Ahn HS, Preziosi TJ, et al: Cerebral arterial spasm–a controlled trial of nimodipine in patients with subarachnoid hemorrhage. N Engl J Med 1983, 308:619-24.
8. Otten ML, Mocco J, Connolly ES Jr, Solomon RA: A review of medical treatments of cerebral vasospasm. Neurol Res 2008, 30:444-9.
9. Komotar RJ, Hahn DK, Kim GH, et al: Efficacy of lamina terminalis fenestration in reducing shunt-dependent hydrocephalus following aneurysmal subarachnoid hemorrhage: a systematic review. Clinical article J Neurosurg 2009, 111:147-54.
10. Kasuya H, Shimizu T, Kagawa M: The effect of continuous drainage of cerebrospinal fluid in patients with subarachnoid hemorrhage: a retrospective analysis of 108 patients. Neurosurgery 1991, 28:56-9.
11. Widenka DC, Wolf S, Schurer L, Plev DV, Lumenta CB: Factors leading to hydrocephalus after aneurysmal subarachnoid hemorrhage. Neurol Neurochir Pol 2000, 34:56-60.
12. Klimo P, Kestle JR, MacDonald JD, Schmidt RH: Marked reduction of cerebral vasospasm with lumbar drainage of cerebrospinal fluid after subarachnoid hemorrhage. J Neurosurg 2004, 100:215-24.
13. Kwon OY, Kim YJ, Cho CS, Lee SK, Cho MK: The Utility and Benefits of External Lumbar CSF Drainage after Endovascular Coiling on Aneurysmal Subarachnoid Hemorrhage. J Korean Neurosurg Soc 2008, 43:281-7.
14. Tuettenberg J, Czabanka M, Horn P, et al: Clinical evaluation of the safety and efficacy of lumbar cerebrospinal fluid drainage for the treatment of refractory increased intracranial pressure. J Neurosurg 2009, 110:1200-8.
15. Staykov D, Speck V, Volbers B, et al: Early recognition of lumbar overdrainage by lumbo-ventricular pressure gradient. Neurosurgery 2011, 68(5):1187-91.
16. Molyneux AJ, Kerr RS, Yu LM, et al: International subarachnoid aneurysm trial (ISAT) of neurosurgical clipping versus endovascular coiling in 2143 patients with ruptured intracranial aneurysms: a randomised comparison of effects on survival, dependency, seizures, rebleeding, subgroups, and aneurysm occlusion. Lancet 2005, 366:809-17.
17. Kreiter KT, Mayer SA, Howard G, et al: Sample size estimates for clinical trials of vasospasm in subarachnoid hemorrhage. Stroke 2009, 40:2362-7.
18. Rankin J: Cerebral vascular accidents in patients over the age of 60. II Prognosis Scott Med J 1957, 2:200-15.
19. Lindley RI, Waddell F, Livingstone M, Warlow CP, Dennis MS, Sandercock PAG: Can simple questions assess outcome after stroke? Cerebrovasc Dis 1994, 4:314-32.
20. Hunt WE, Hess RM: Surgical risk as related to time of intervention in the repair of intracranial aneurysms. J Neurosurg 1968, 28:14-20.

21. Fisher CM, Kistler JP, Davis JM: Relation of cerebral vasospasm to subarachnoid hemorrhage visualized by computerized tomographic scanning. Neurosurgery 1980, 6:1-9.
22. Scheithauer S, Burgel U, Ryang YM, et al: Prospective surveillance of drain associated meningitis/ventriculitis in a neurosurgery and neurological intensive care unit. J Neurol Neurosurg Psychiatry 2009, 80:1381-5.

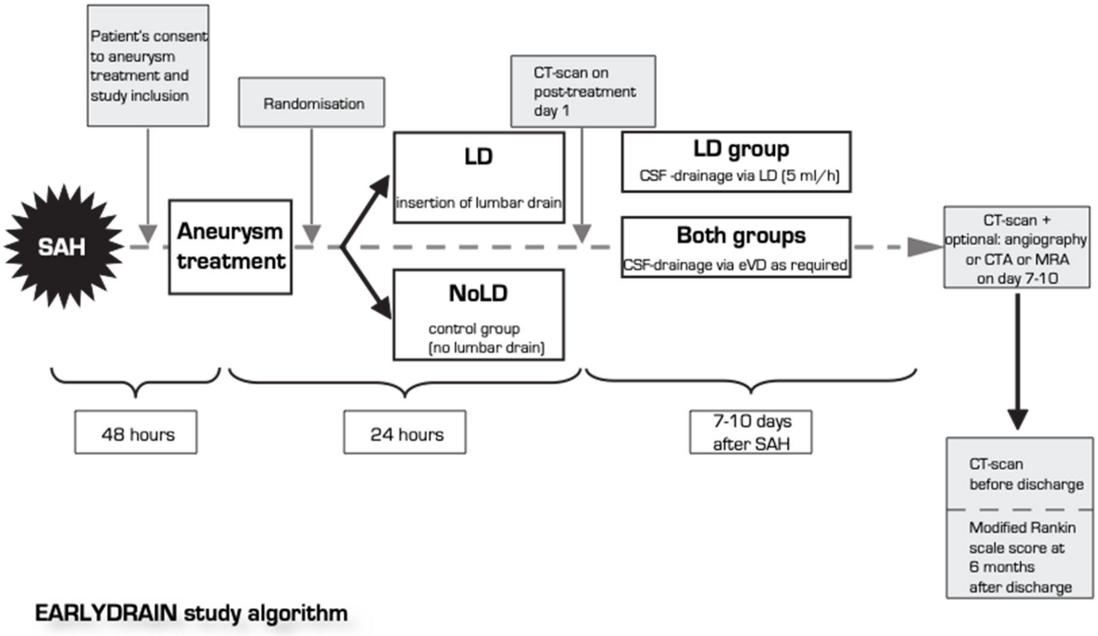

Figure 1: EARLYDRAIN study algorithm.
The EARLYDRAIN study algorithm showing the course of events after the initial aneurysmal subarachnoid hemorrhage (SAH) and the subsequent surgical or interventional aneurysm treatment. The study includes two groups, a treatment group receiving lumbar CSF-drainage (LD) and a control group receiving no lumbar drainage according to protocol (NoLD). The timing of the patient's consent to study participation, randomization, cranial imaging and assessment of clinical outcome is indicated by the shaded boxes. Imaging on day 7 to 10 is scheduled according to local guidelines. If a local center performs no routine cerebrovascular imaging for vasospasm screening in patients without clinical suspicion, it may be omitted.

This was the first version of the EARLYDRAIN study protocol, dated December 9th, 2010. This version was submitted for approval to the lead institutional review board, the Ethical Committee of the medical faculty, Friedrich-Alexander-University Erlangen-Nürnberg, Germany (reference number 4171). All authors agreed on this version. Recruitment of patients started in January 2011.

EARLYDRAIN - Study Protocol

Stefan Wolf, Jürgen Bardutzky, Eric Jüttler, Peter Vajkoczy, Stefan Schwab

Objective:

to investigate whether early application of a lumbar drainage improves clinical outcome after aneurysmal subarachnoid hemorrhage.

Background:

Patients suffering from aneurysmal subarachnoid hemorrhage (SAH) are predominantly threatened by two distinct medical problems. Firstly, they may experience a second – and often more severe – hemorrhage, and secondly, they may suffer a constringency reaction of the vessels supplying the brain with blood, called vasospasm.

The first problem is solved through rapid cerebrovascular imaging and subsequent treatment of the ruptured aneurysm, thus preventing recurrent hemorrhage. Aneurysm treatment may be performed either via craniotomy and surgical clipping of the aneurysm or with endovascular techniques by occluding the aneurysm with small platinum coils.

The vasospasm - the second problem - is more difficult to handle. The incidence of symptomatic vasospasm is about 30 to 60% after aneurysmal SAH, depending on definition (1). The sequelae of cerebral vasospasm are permanent neurologic deficits, including death, due to infarction of the brain. Clinical signs of vasospasm include neurologic decline, hemiparesis or any other focal neurologic deficit not explained by other reasons like posthemorrhagic hydrocephalus or electrolyte imbalances. The pathomechanism leading to vasospastic vessel constriction is incompletely understood (2). Diagnostic procedure of choice is the digital subtraction angiography. Vasospasm may be present in the proximal vessels, the distal branches of the vasculature or both.

Prophylactic application of the calcium channel blocker nimodipine is currently the only therapy recognized for prevention of vasospasm (3). Newer approaches currently not included in official guidelines but performed in several centers are medication with statins and magnesium (4).

A hypothesis is that the development of vasospasm is related to the amount of blood in the basal cisterns. Therefore, a possible strategy tries to remove this blood as much as possible. If the aneurysm leading to the initial hemorrhage is secured via surgical therapy, some surgeons prefer to open the terminal lamina and irrigate the blood from the basal cisterns. Albeit promising, studies addressing this approach show mixed results (5). Opening of the basal cisterns and irrigation of the blood is not feasible if the aneurysm is secured with endovascular techniques.

Excess removal of cerebral spinal fluid (CSF) via an external ventricular drain fails in preventing vasospasm and may lead to a higher incidence of posthemorrhagic shunt dependency (6; 7). Reason is that after aneurysmal SAH, the blood is packed more densely in the basal cisterns and therefore only CSF, being more lightweight, is drained from the ventricles. As an alternative approach, application of a lumbar drain is proposed to address clotting of the blood in the basal cisterns. Three retrospective studies in patients after aneurysmal SAH, the newest being available only as abstract, were able to establish the safety of this approach (8-10). One of the fully published studies addressed vasospasm prophylaxis after surgical clipping (8), while the other was performed in patients after endovascular coiling (9). All studies led to a markedly diminished incidence of angiographic vasospasm. Therefore, a prospective study addressing the efficacy of this novel therapeutic approach is warranted.

The focus of the EARLYDRAIN study is to examine the efficacy of application of lumbar drainage in patients with acute subarachnoid hemorrhage from a cerebral aneurysm. Hypothesis is that early application of lumbar drainage after aneurysmal SAH leads to a diminished incidence of cerebral vasospasm, as assessed by digital subtraction angiography, and an improved outcome, measured by the modified Rankin score, at six months.

Study outline

Patients suffering from aneurysmal SAH are treated according to international standards. Aneurysm treatment is at the discretion of the neurovascular team taking care for a patient and not specified by the study protocol. All medical treatment is performed according to local guidelines and standard operating procedures.

Any patient meeting the inclusion criteria and not violating the exclusion criteria may participate in the EARLYDRAIN study and be randomized to either receive a lumbar drain or not, thus defining the two distinct groups LD and NoLD. To prevent premature rupture of the aneurysm due to accidental drainage, randomization to the study and eventual placement of a lumbar drain takes place after securing the aneurysm by the preferred method of choice. Any patient in the LD group receives a lumbar drain during anesthesia required for aneurysm treatment. This is to be performed before anticoagulation or anti-platelet therapy is initiated, which sometimes is warranted after endovascular coiling. A post-procedural CCT scan of the brain is performed within to 24 hours of aneurysm treatment. In case of any neurological worsening after the procedure it is strongly recommended to perform the follow-up CCT scan as soon as possible.

In patients in the LD group, CSF drainage is started via LD slowly and steadily at a rate of approximately 5 ml per hour after the post-interventional CCT. This leads to a planned daily CSF drainage of about 120 ml per day via lumbar route. Patients in both groups may receive additional CSF drainage via a ventricular device as required. The amount of CSF drained via ventricular route is according to clinical requirement and not specified.

To facilitate accuracy of drainage, regular drainage control every other hour and stopping in case of excess drainage is strongly recommended by the principal investigators. In case of neurological decline suspiciously related to the lumbar drainage, the drain is closed immediately and may be gradually restarted after 12 to 24 hours, after performing a CCT scan.

If the post-procedural CCT or any other follow-up CCT scan shows absent basal cisterns or any signs of threatening herniation, lumbar CSF diversion in the LD group shall not be performed. It may still be

feasible to carefully drain CSF via the lumbar route may (11), but this is at the discretion of the local investigator and not recommended.

In patients requiring sedation and mechanical ventilation, either due to neurological impairment or otherwise, intracranial pressure monitoring is mandatory. This may be performed according to local policy either with parenchymal or ventricular devices. If the intracranial pressure exceeds 20 mmHg, further CSF drainage via lumbar route shall be interrupted until the ICP is below 20 mmHg again. Careful CSF drainage via the lumbar route may be still feasible in case of high intracranial pressure (11), but is at the discretion of the local investigator.

Further neuromonitoring with TCD, EEG, brain tissue oxygenation, jugular bulb oxymetry, regional cerebral blood flow, microdialysis or other devices is at the discretion of the center and according to its local guidelines. As far as possible, this data should be saved electronically for post-hoc analysis.

A CCT scan as well as conventional digital subtraction angiography, CT angiography or MR angiography for assessment of vasospasm in the larger vessels is routinely performed on day 7 to 10 after the initial hemorrhage, regardless of the patient condition. In case of clinical suspicion of vasospasm, angiography may be performed at any time. If it is performed earlier and the patient shows no clinical deterioration thereafter, the angiography on day 7 to 10 is omitted.

After cerebrovascular imaging on day 7 to 10, or day 8 in case of an earlier angiography, the lumbar drainage of CSF is stopped in the LD group. It may be pursued on a clinical base, as required.

Amount and duration of CSF drainage

Patients randomized to the lumbar drainage group shall receive a daily drainage of 120 ml CSF, or 5 ml per hour for seven days. If higher amounts of CSF need to be drained on clinical grounds as in patients with hydrocephalus, this is preferably performed via external ventricular drain.

The drain is planned to remain in place until the control angiography on day 7 to 10 after the initial hemorrhage. The local investigator may decide to remove the drain earlier in patients fully mobilized without clinical necessity of CSF drainage. However, consecutive drainage should not be less than four

days to achieve a valid study result. Lumbar CSF drainage may be prolonged beyond the control angiography on clinical requirement. The amount of CSF drainage may then be adjusted to clinical needs and bears no further restriction.

Patients randomized to the control group should not receive a lumbar drain before the planned control angiography to be performed on day 7 to 10 after SAH. If the patient develops hydrocephalus, and no EVD was placed initially for CSF drainage, a lumbar drain may be installed at the discretion of the local investigator. These patients are analyzed in the intention-to-treat analysis, but are not suitable for per-protocol analysis.

Study in- and exclusion criteria

Inclusion criteria:

- Age of 18 years or older
- First aneurysmal SAH
- Pre-morbid modified Rankin Scale score 0 or 1
- Aneurysm treatment performed in the first 48 hours after the initial hemorrhage.
- Informed consent by the patient or his/her legal representative. In case neither the patient is capable of giving informed consent nor a legal representative is available, informed consent can be given by an independent physician neither involved in the patient's treatment nor the trial (for specification see below)

Exclusion criteria:

- Subarachnoid hemorrhage of other than aneurysmal origin
- No hemorrhage visible on initial CCT scan (Fisher Grade I)
- Pregnancy
- Concurrent participation in another interventional trial (participation in an observational trial is allowed)
- Life expectancy less than 1 year for other reasons than the actual SAH
- Other concomitant severe disease that would confound with treatment

- Other clear contraindication for treatment

Consent to the study

Consent for study inclusion is sought after explanation and agreement to a specific aneurysm treatment. Thus, patients capable of consenting to the aneurysm treatment get the study details explained themselves and may or may not agree to participate. If a patient is incapable for consenting to the proposed treatment, the legal representative should be informed on the conditions of treatment choices and afterwards, on the details of the EARLYDRAIN study. A patient may be randomized if the legal representative gives informed consent to the study, based on the presumed will of the patient. If neither the patient is capable of giving informed consent nor a legal representative is available in due time, an independent physician not involved in the patient's treatment nor in the trial may be asked for study approval. In these cases of deferred consent, a legal representative needs to be established as soon as possible, according to German law. As soon as a legal representative is available and/or the patient is capable again to consent to the study, he or she must be asked to give informed consent. If the patient or his/her legal representative refuses consent after inclusion by advice of an independent physician, no further study participation of the patient is possible. In this case, however, the patient or his/her legal representative are asked to give consent for evaluation of already acquired data.

Safety of lumbar drains after aneurysmal SAH

In all three retrospective studies, mortality was lower in the lumbar drainage group. None of the retrospective studies mentions procedural related complications for the lumbar drains (8-10). In patients with increased intracranial pressure, careful lumbar drainage of CSF may be a possible treatment even in case of compressed basal cisterns (11). To date, there is no data available indicating an increased risk of lumbar drainage in a controlled neurointensive care environment.

Insurance coverage

As the EARLYDRAIN study compares two standard procodures of CSF drainage after subarachnoid hemorrhage used in clinical routine, no additional patient insurance is necessary to perform the study. German laws §§ 40 to 42 Arzneimittelgesetz or §§ 20 to 23 Medizinproduktegesetz are not applicable.

Any hypothetical adverse events of either treatment are covered by the regular treatment contracts which do include clinical research.

Outcome assessment:

The primary endpoint is disability after 6 months, assessed by the modified Rankin Scale (12; 13), dichotomized at a score of 0 to 2 versus 3 to 6 (6=death). Assessment is performed by a blinded investigator of the local study center by personal visit. Alternatively, a telephone questionnaire is suitable for outcome assessment of the modified Rankin Scale (13). Outcome assessment is planned to be done on the whole dataset as well as in preplanned stratified subsets (i.e. for example clinical SAH grade according to the Hunt&Hess scale 1-2 vs. 3-5 (14), CT grading according to Fisher I-III vs. IV (15)).

Secondary outcome criteria include:

- Mortality after 6 months
- mRS score after 6 months as continuous variable
- Angiographic vasospasm at day 7 to 9, as defined by a caliber reduction by 33% or more compared to the initial digital subtraction angiography
- Vasospastic infarction in the last CT scan before discharge
- Expression of clinical delayed neurological deficit after the aneurysmal SAH until discharge from acute care.
- Daily course of TCD mean flow velocity in both MCA at a depth of 50-60 mm
- Rate of death during the initial hospital treatment after the aneurysmal SAH.
- Rate of CSF shunt insertion in the first six months
- Presence of CSF infection during the first 14 days, as defined by modified CDC criteria for device-associated meningitis (treatment required on either positive culture, or elevated cell count, red cell/ white cell ratio, increased lactate and/or decreased glucose). (16)

The following parameters will be recorded and used in predictor-/association models concerning primary and/or secondary outcome parameters:

- Gender
- Age
- Hunt&Hess Scale on admission
- Time from symptom onset to admission
- Localisation of aneurysm
- Time from symptom onset to aneurysm treatment
- Treatment of aneurysm by clipping or coiling or both
- Time from symptom onset to randomization
- Time from symptom onset to treatment start (i.e. insertion of the lumbar drainage in the treatment arm)
- Time from admission to discharge
- Insertion of EVD (yes/no)
- Duration of EVD
- Duration of lumbar drainage
- Amount of CSF drainage drained by EVD (ml)
- Amount of CSF drainage drained by lumbar drainage (ml)
- Use of Nimodipine (yes/no)
- Use of statins (yes/no)
- Use of Mg^{2+} (yes/no)
- Transcranial Doppler ultrasound in both MCA at 50-60 mm depth, 1x daily (>160 cm/s versus <160 cm/s)
- Presence of CSF infection during hospital stay (yes/no)

Serious adverse events (SAE)

Adverse events, risks

Every patient is treated according to international guidelines. 50% of patients are additionally treated by lumbar drainage.

Surgery-related complications: Surgical treatment includes the known risks of surgical interventions.

Complications related to endovascular therapy: Endovascular therapy includes the risks known to be associated with.

Definition of adverse events and severe adverse events

The term „adverse event“ (AE) describes any sign, symptom, syndrome or any disease 1. occurring newly in a trial participant after consent to the trial and 2. being of particular interest for the assessment of the disease or the security of the therapeutic concept. In this trial AEs include:

- arterial or venous thromboses,
- complications related to insertion of a lumbar drainage,
- any SAE

The term AE does not implicate a causal correlation with the participation in the trial. Surgical interventions are not necessarily considered as AE but can be necessary for the therapy of an AE. AEs are divided in severe (SAE) and not severe (AE) adverse events.

A SAE is any AE occurring during the trial that is related to:

- death
- any live-threatening condition,
- re-hospitalisation or prologation of hospitalisiation,
- long-term or severe restraint of the state of health, or
- birth deformities.

Documentation

Investigation of AEs is part of every visit and any AE has do be documented in the CRF.

Every SAE has to be documented on a special documentation form and has to be reported within 24 hours after recording, but at least at the next working day, to the data monitoring center:

CSB Centrum für Schlaganfallforschung Berlin

CSB Sekretariat

Charité - Universitätsmedizin Berlin

Campus Mitte

Charitéplatz 1

10117 Berlin

Fon +49 30 450 xxx xxx

Fax +49 30 450 xxx xxx

csb@charite.de

The CSB informs the principial investigators of the study of the occurrence of SAEs. SAEs may require to be reported to the ethics committee according to local standards. The Data Safety and Monitoring Board (DSMB) has to be informed about SAEs at least every 6 months, starting with the day of inclusion of the first patient. The responsible study center has to pursue further changes and outcomes of SAEs, regarding intensity and potential relations to treatment. Evaluation of SAEs is performed by CSB.

Emergency

The occurance of a SAE does not automatically implicate a preliminary stop of the patient's participation in the trial, but requires immediate initiation of diagnostic and therapeutic measures for the protection of the patient's health.

Principal investigators:

Stefan Wolf, M.D.

Department of Neurosurgery, Charité Berlin, Augustenburger Platz 1, 13353 Berlin, Germany

stefan.wolf@charite.de

and

Jürgen Bardutzky, M.D., Ph.D., Department of Neurology, Albert-Ludwigs-University Freiburg, Breisacher Straße 64, 79106 Freiburg, Germany

juergen.bardutzky@uniklinik-freiburg.de

Study sites:

Study sites to participate in the EARLYDRAIN trial should treat at least 30 patients with aneurysmal subarachnoid hemorrhage per year. Preferably, to decrease the variability between centers and ensure adequate recruitment frequency, a maximum of 8 to 10 centers is warranted.

Promotion of the EARLYDRAIN study is performed at the next national meetings of the German societies of Neurology and Neurosurgery as well as direct propagation.

Ethic approval:

Each participating center seeks for its own ethic approval. The ethic vote from the principal investigators should be provided. On request from local investigators, the data monitoring center (Centrum für Schlaganfall-Forschung Berlin, see below) is able to provide assistance for the ethic approval.

Trial registration

The trial protocol is registered on the internet at www.clinicaltrials.gov.

Study size planning

In the ISAT trial, the largest trial on the treatment of aneurysmal subarachnoid hemorrhage so far, the mortality at one year follow-op was about 8.1% to 10.1% (17). Given the data from both fully published studies on lumbar drains after SAH, a reduction from 15% to 2.1% after coiling and from 5% to 3 % after clipping was shown. Thus, both studies were way lower in their mortality rate and, therefore, their external validity may be questioned.

In the three retrospective trials, 167, 107 and 79 patients were studied. The effect of LD was a decrease in the incidence of vasospasm by 34%, 40% and 54%, respectively. However, the definition of endpoints was not equal in the retrospective studies.

To assess a decrease of the incidence of clinical vasospasm from 40% to 20% in a prospective clinical trial, 93 patients in each of the two study arms are required to gain a power of 85%, using an alpha error of 5%. To account for possible imbalances in the randomization procedure concerning severity of clinical and radiological grading of the SAH or the choice of treatment and to facilitate a preplanned analysis on the severity of the initial hemorrhage, the planned study size is to include and randomize altogether 300 patients. This gives a power of 85.2%, again using an alpha error of 5%, to detect a decrease in the rate of severe disability on a dichotomized modified Rankin scale from 50% to 33%, which would be consistent with the effect size from the retrospective trials (8-10).

The EARLYDRAIN investigators are aware of other, more conservative calculations for the sample size of vasospasm studies, indicating there may be the necessity to include more than 5000 patients in a single trial (18). The power calculations, as described above and based on the available retrospective data, do not substantiate numbers this large. Besides feasibility issues, clinical experience from the principal investigators considering the expected effort-benefit ratio does not warrant enlargement of the trial to detect a rather small difference between groups.

Statistical Analysis

All data are described according to their mean, median or frequency, as applicable. The dichotomized modified Rankin score as target outcome variable is investigated univariately against treatment group. Multivariate logistic regression modeling is performed accordingly, adjusted for clinical grade, fisher grade, ventricular hemorrhage, parenchymal hemorrhage, gender, nimodipine or other concomitant medical treatment. Analysis is planned as intention-to-treat as well as per protocol, excluding the patients who were treated with amounts of CSF drainage via lumbar drain deviating from the specified 5 ml/h or which needed a lumbar drain when randomized to the No-LD group.

Interim Analysis

An interim analysis after inclusion of 150 patients is planned to address safety issues. This analysis focuses on the secondary endpoints and SAEs only, especially the rate of death during the hospital stay. During the interim analysis, the recruitment for the EARLYDRAIN study is not stopped.

Randomization:

Randomization is performed via a dedicated internet site accessible for all local investigators of the participating trial centers (www.randomizer.org). Application of a lumbar drain is performed accordingly, while the patient is still under anesthesia for aneurysm treatment.

At each participating center, a local database of all patients treated with aneurysmal SAH is to be established. In this database, reasons for patient exclusion should be documented (e.g. missing consent, aneurysm treatment not possible in the first 48 hours after aneurysmal SAH).

Study duration and other time points

Start of the study is November 2010. Depending on center participation and patient recruitment, inclusion of 300 patients is expected to be finished by the end of 2011. As the main endpoint of the study is the MRS after 6 months, study completion is planned summer 2012. Data gathering and analysis is planned to be finished fall 2012. Preparation and submission of a manuscript describing the results to a leading international Journal is planned in autumn 2012.

Intellectual properties

The principal investigators retain the rights for first and last authorship of the manuscript containing the main results. The local investigators of the participating centers are granted access to the anonymized whole data set as well as co-authorship on the manuscript with the main results. The rank of co-authorship is determined by decreasing inclusion frequency. A separate trial contract is performed between a participating center and the principal investigators.

After acceptance of the main manuscript, participating centers may query the anonymized database on own research questions and for own publications. As EARLYDRAIN is a collaborative study, the whole study group shall receive credit on additional manuscripts (eg. "and the EARLYDRAIN Study Group", with mentioning of local investigators and participating centers in an appendix or a footnote.). For coordination purposes and to avoid ambiguity of intellectual properties, this additional research and resulting publications require to be harmonized with the principal investigators.

Data monitoring and surveillance site

The study is monitored by the Centrum für Schlaganfallforschung of the Charite, Humboldt University Berlin:

CSB Sekretariat
Charité - Universitätsmedizin Berlin
Campus Mitte
Charitéplatz 1
10117 Berlin
Fon +49 30 450 xxx xxx
Fax +49 30 450 xxx xxx
csb@charite.de

A dedicated study nurse visits the participating centers according to their recruiting frequency. During this visit, the clinical files of a recruited patient are reviewed on their consistency with the EARLYDRAIN data sheet. The study center performs the telephone interviews for outcome assessment. Additionally, on request from local investigators, the data monitoring is able to provide assistance for the initial local ethic approval.

References:

1. Keyrouz SG, Diringer MN. Clinical review: Prevention and therapy of vasospasm in subarachnoid hemorrhage. Crit Care 2007;11(4):220.

2. Pluta RM, Hansen-Schwartz J, Dreier J, Vajkoczy P, Macdonald RL, Nishizawa S, Kasuya H, Wellman G, Keller E, Zauner A, Dorsch N, Clark J, Ono S, Kiris T, Leroux P, Zhang JH. Cerebral vasospasm following subarachnoid hemorrhage: time for a new world of thought. Neurol. Res 2009;31(2):151-158.

3. Allen GS, Ahn HS, Preziosi TJ, Battye R, Boone SC, Boone SC, Chou SN, Kelly DL, Weir BK, Crabbe RA, Lavik PJ, Rosenbloom SB, Dorsey FC, Ingram CR, Mellits DE, Bertsch LA, Boisvert DP, Hundley MB, Johnson RK, Strom JA, Transou CR. Cerebral arterial spasm--a controlled trial of nimodipine in patients with subarachnoid hemorrhage. N. Engl. J. Med 1983;308(11):619-624.

4. Otten ML, Mocco J, Connolly ES, Solomon RA. A review of medical treatments of cerebral vasospasm. Neurol. Res 2008 Jun;30(5):444-449.

5. Komotar RJ, Hahn DK, Kim GH, Starke RM, Garrett MC, Merkow MB, Otten ML, Sciacca RR, Connolly ES. Efficacy of lamina terminalis fenestration in reducing shunt-dependent hydrocephalus following aneurysmal subarachnoid hemorrhage: a systematic review. Clinical article. J. Neurosurg 2009;111(1):147-154.

6. Kasuya H, Shimizu T, Kagawa M. The effect of continuous drainage of cerebrospinal fluid in patients with subarachnoid hemorrhage: a retrospective analysis of 108 patients. Neurosurgery 1991;28(1):56-59.

7. Widenka DC, Wolf S, Schürer L, Plev DV, Lumenta CB. Factors leading to hydrocephalus after aneurysmal subarachnoid hemorrhage. Neurol. Neurochir. Pol 2000;34(6 Suppl):56-60.

8. Klimo P, Kestle JRW, MacDonald JD, Schmidt RH. Marked reduction of cerebral vasospasm with lumbar drainage of cerebrospinal fluid after subarachnoid hemorrhage. J. Neurosurg 2004;100(2):215-224.

9. Kwon OY, Kim Y, Kim YJ, Cho CS, Lee SK, Cho MK. The Utility and Benefits of External Lumbar CSF Drainage after Endovascular Coiling on Aneurysmal Subarachnoid Hemorrhage. J Korean Neurosurg Soc 2008;43(6):281-287.

10. Tizi K, Güresir E, Beck J, Raabe A, Seifert V, Vatter H. The effect of lumbar drainage on cerebral vasospasm and infarction after subarachnoid haemorrhage (abstract). 60th annual meeting of the German Society of Neurosurgery (DGNC) 2009;

11. Tuettenberg J, Czabanka M, Horn P, Woitzik J, Barth M, Thomé C, Vajkoczy P, Schmiedek P, Muench E. Clinical evaluation of the safety and efficacy of lumbar cerebrospinal fluid drainage for the treatment of refractory increased intracranial pressure. J. Neurosurg 2009;110(6):1200-1208.

12. Rankin J. Cerebrovascular accidents in patients over the age of 60: prognosis. Scott Med J 1957;(2):200-215.

13. Lindley RI, Waddell F, Livingstone M, Sandercock P, Dennis MS, Slattery J, Smith B, Warlow C. Can Simple Questions Assess Outcome after Stroke? Cerebrovasc Dis 1994;4(4):314-324.

14. Hunt WE, Hess RM. Surgical risk as related to time of intervention in the repair of intracranial aneurysms. J. Neurosurg 1968;28(1):14-20.

15. Fisher CM, Kistler JP, Davis JM. Relation of cerebral vasospasm to subarachnoid hemorrhage visualized by computerized tomographic scanning. Neurosurgery 1980;6(1):1-9.

16. Scheithauer S, Bürgel U, Ryang Y, Haase G, Schiefer J, Koch S, Häfner H, Lemmen S. Prospective surveillance of drain associated meningitis/ventriculitis in a neurosurgery and neurological intensive care unit. Journal of Neurology, Neurosurgery & Psychiatry 2009 Dec;80(12):1381-1385.

17. Molyneux AJ, Kerr RSC, Yu L, Clarke M, Sneade M, Yarnold JA, Sandercock P. International subarachnoid aneurysm trial (ISAT) of neurosurgical clipping versus endovascular coiling in 2143 patients with ruptured intracranial aneurysms: a randomised comparison of effects on survival, dependency, seizures, rebleeding, subgroups, and aneurysm occlusion. Lancet 2005;366(9488):809-817.

18. Kreiter KT, Mayer SA, Howard G, Knappertz V, Ilodigwe D, Sloan MA, Macdonald RL. Sample size estimates for clinical trials of vasospasm in subarachnoid hemorrhage. Stroke 2009 Jul;40(7):2362-2367.

Summary of changes between initial and final EARLYDRAIN protocols

Besides clarification in scientific reasoning and wording, the final protocol includes:

- More accurate background information on aneurysmal subarachnoid hemorrhage, including more references to other works and a short discussion of the LUMAS trial, which was pending completion.
- Mentioning of trial approval by the Ethical Committee of the medical faculty, Friedrich-Alexander-University Erlangen-Nürnberg, Germany (reference number 4171)
- Mentioning of the trial registration at clinicaltrials.gov, NCT01258257
- A flowchart of the EARLYDRAIN study algorithm
- More detailed description of clinical and study procedures
- Extension of the list of secondary endpoints of interest
- Specification of logistic regression as method of analysis
- A discussion of the study and its importance in the field. Justification of the primary outcome parameter being clinical performance instead of surrogate endpoints like delayed neurologic deficit or the role of radiographic vasospasm.
- Description of Data and Safety Monitoring Board and Steering Committee
- A list of collaborating centers and investigators
- Explicit mentioning that there will be no funding for trial participation.

In the final protocol, removed from the initial protocol were:

- Contact information for the *CSB Centrum für Schlaganfallforschung, Berlin, Germany*
- Overly enthusiastic time schedule for the Earlydrain trial

The final protocol was partially rewritten, reformatted and logical flow reordered to meet the publication requirements of the Trials journal.

Statistical Analysis Plan

No dedicated separate statistical analysis plan was written for Earlydrain. The relevant procedures were described in the final protocol (Bardutzy et al, Trials 2011, 12:203).

Primary endpoint is disability after 6 months, assessed by the modified Rankin Scale and dichotomized at a score of 0 to 2 versus 3 to 6 (6 = death).

Outcome assessment was planned to be done on the whole dataset as well as in preplanned stratified subsets (for example clinical SAH grade according to the Hunt & Hess scale 1-2 vs. 3-5, CT grading according to Fisher I-III vs. IV).

Prespecified secondary outcome criteria were:

- Mortality after 6 months
- mRS score after 6 months as continuous variable
- Angiographic vasospasm on day 7 to 10, as defined by a caliber reduction by 33% or more compared to the initial digital subtraction angiography
- Endovascular rescue therapy performed due to proven vasospasm, using balloon dilation of spastic vessels and/or arterial infusion of vasodilators
- Infarction (due to vasospasm) in the last CT scan before discharge
- Expression of clinical delayed neurological deficit after the aneurysmal SAH until discharge from acute care.
- Daily course of TCD mean flow velocity in both MCA at a depth of 50-60 mm
- Rate of death during the initial hospital treatment after the aneurysmal SAH.
- Rate of CSF shunt insertion during the first six months
- Presence of CSF infection during the first 14 days, as defined by modified CDC criteria for device-associated meningitis (treatment required on either positive culture, or elevated cell count, red cell/white cell ratio, increased lactate and/or decreased glucose).

Further parameters recorded and used for treatment group comparison in predictor / association models were:

- Gender
- Age
- Hunt&Hess grade on admission
- Time from symptom onset to admission
- Location of aneurysm
- Time from symptom onset to aneurysm treatment
- Treatment of aneurysm by clipping or coiling or both
- Time from symptom onset to randomization
- Time from symptom onset to treatment start (i.e. insertion of the lumbar drainage in the treatment arm)
- Time from admission to discharge
- Insertion of EVD (yes/no)
- Duration of EVD being in place
- Duration of lumbar drainage
- Amount of CSF drained by EVD [ml]
- Amount of CSF drained by lumbar drain [ml]
- Use of nimodipine (yes/no)
- Use of statins (yes/no)
- Use of Mg^{2+} (yes/no)
- Transcranial Doppler ultrasound in both MCA at 50-60 mm depth, 1x daily (> 160 cm/s versus < 160 cm/s)
- Presence of CSF infection during hospital stay (yes/ no)

Method of analysis for the primary endpoint is univariate logistic regression (R function glm (… , family = binomial (link="logit")). This was chosen to allow for easy expansion to multivariate analysis for adjustment for known factors associated with outcome, like age or clinical severity grade.

All data are described according to their mean, median or frequency, as applicable.

Case Report Form (English version)

This is the English version of the EARLYDRAIN Case Report Form. A German version was available, too, translated by the primary investigator. Both were in printed in paper, providing a copy for the local site. Data was entered in a Microsoft Access database by double entry after finishing of recruitment.

EARLYdrain - Visitation 1 - Screening and admission
Pat.ID □□□ Study Center ID □□□ Date_____________

Status on study inclusion

Modified Fisher Scale on admission (*)

Grade I No blood detected.

Grade II Diffuse subarachnoid layer less than 1 mm thick

Grade IIIa Localized clot and/or vertical layers 1 mm or more in thickness, no intraventricular hemorhage

Grade IIIb Localized clot and/or vertical layers 1 mm or more in thickness and with intraventricular hemorhage

Grade IVa all SAH with intracerebral hematoma, no intraventricular hemorhage

Grade IVb all SAH with intracerebral hematoma and additional intraventricular hemorrhage

Hunt and Hess grade on *admission*

(without correction for severe systemic disease)

Grade I Asymptomatic or mild headache, no or only slightly stiff neck

Grade II Severe headache, stiff neck, no neurologic deficit except cranial nerve palsy

Grade III Drowsy or confused, mild focal neurologic deficit

Grade IV Stuporous, moderate or severe hemiparesis

Grade V Deep coma, decerebrate posturing, moribund appearance

Age of the patient: ______ years
sex: □ male □ female

height: _______ cm

weight: __________ kg

(*) This scale was conceived to serve as combined inquiry for the original Fisher scale (Fisher et al, Neurosurgery 1980) as well the modified Fisher scale (Frontera et al, Neurosurgery 2006). During data analysis, it was recognized that it erroneously did not allow to distinguish between modified Fisher grades I and II (mild SAH without or with intraventricular hemorrhage). All centers were asked to reevaluate the original CT scans of all patients on this item.

EARLYdrain - Visitation 1 - Screening and admission
Pat.ID □□□ Study Center ID □□□ Date_____________

Diagnostic Imaging

Time of first cranial CT:

Date ______________ DD MM YYYY Time ___________ hh mm

(final) detection of aneurysm:

- o conventional angiography (DSA)
- o MRA
- o CTA

Date ______________ DD MM YYYY Time ___________ hh mm

Localisation of the ruptured aneurysm:

Middle cerebral artery (MCA) o
Anterior communicating artery (ACoA) o
Posterior communicating artery (PCoA) o
Anterior cerebral artery (ACA) o
Internal carotid artery (ICA) o
Posterior cerebral artery(PCA) o
Vertebral artery (VA) o
Basilar artery (BA): o
Other: _______________________ o

Aneurysm size (diameter) ________ mm

In case of bilaterally present arteries: o right o left

Multiple aneurysms: o yes o no

If yes, number of aneurysms: _______

EARLYdrain - Visitation 1 - Screening and admission
Pat.ID □□□ Study Center ID □□□ Date_____________

When did the subarachnoid hemorrhage occur?

Date ______________ DD MM YYYY Time ___________ hh mm

Glasgow Coma Scale (GCS) on *admission*

Eye opening

- ○ 4 – spontaneous
- ○ 3 – response to verbal command
- ○ 2 – response to pain stimulus
- ○ 1 – No reaction

Verbal Communication

- ○ 5 – able to speak, oriented
- ○ 4 – able to speak, disoriented
- ○ 3 – incoherent words
- ○ 2 – incomprehensible sounds
- ○ 1 – No verbal reaction

Motor response

- ○ 6 – obeys commands
- ○ 5 – localizing response to pain
- ○ 4 – withdrawal response to pain
- ○ 3 – flexion to pain
- ○ 2 – extension to pain
- ○ 1 – No motor response to pain

Paresis/Speech impairment on *admission*

Is paresis present?

□ No □ Paresis □ Plegia

Does the patient have a speech impairment?

□ No □ Sensory □ Motor

EARLYdrain - Visitation 1 - Screening and admission
Pat.ID □□□ Study Center ID □□□ Date_____________

Modified Rankin Scale (mRS) (Status before the current SAH)

0 – no symptoms

1 - No significant disability despite some symptoms; but able to carry out all usual duties and activities

2 - Slight disability; unable to carry out all previous activities, but able to look after own affairs without assistance

3 – moderate disability; requiring some help, but able to walk without assistance

4 – moderate severe disability; unable to walk without assistance and unable to attend to own bodily needs without assistance

5 - Severe disability; bedridden, incontinent and requiring constant nursing care and attention

Details of the patient´s consent to study inclusion

- Consent given by the patient
- Consent given by legal representative
- Consent given by independent physician not involved in conducting the trial

Date of consent: ____________ DD MM YYYY

EARLYdrain - Visitation 1 - Screening and admission
Pat.ID □□□ Study Center ID □□□ Date_____________

Study inclusion criteria	Yes	No
Age >=18 years	O	O
First aneurysmal SAH	O	O
Pre-SAH Status mRS <= 1	O	O
Treatment of aneurysm (surgical or interventional) within 48 hours after symptom onset has been accomplished	O	O
Written consent has been given by the patient OR by the patient's legal representative OR by an independent physician (in case the patient is not able to give informed consent and does not have a legal representative)	O	O

Study exclusion criteria		
Non-aneurysmal SAH	O	O
Head CT does not show SAH (Fisher grad I)	O	O
Pregnancy	O	O
Participation in another interventional study (simultaneous participation in an observational study, including studies using invasive monitoring devices, does NOT constitute an exclusion criterion)	O	O
Life expectancy < 1 year for other reasons than the current SAH	O	O
Other severe medical conditions that might interfere with study treatment	O	O
Other clear indicators that would constitute exclusion from treatment	O	O

EARLYdrain - Visitation 1 - Screening and admission
Pat.ID □□□ Study Center ID □□□ Date_____________

Worst neurological status *before intubation and intervention*

Glasgow Coma Scale (GCS) *before intervention*

Eye opening

- 4 – spontaneous
- 3 – response to verbal command
- 2 – response to pain stimulus
- 1 – No reaction

Verbal communication

- 5 – able to speak, oriented
- 4 – able to speak, disoriented
- 3 – incoherent words
- 2 – incomprehensible sounds
- 1 – No verbal reaction

Motor response

- 6 – obeys commands
- 5 – localizing response to pain
- 4 – withdrawal response to pain
- 3 – flexion to pain
- 2 – extension to pain
- 1 – No motor response to pain

Hunt and Hess grade *before intervention*

(without correction for severe systemic disease)

Grade I Asymptomatic or mild headache, no or only slightly stiff neck

Grade II Severe headache, stiff neck, no neurologic deficit except cranial nerve palsy

Grade III Drowsy or confused, mild focal neurologic deficit

Grade IV Stuporous, moderate or severe hemiparesis

Grade V Deep coma, decerebrate posturing, moribund appearance

Paresis/Speech impairment *before intervention*

Is paresis present?

□ No □ Paresis □ Plegia

Does the patient have a speech impairment?

□ No □ Sensory □ Motor

EARLYdrain - Visitation 1 - Screening and admission
Pat.ID □□□ Study Center ID □□□ Date_____________

Aneurysm treatment and randomization

Method of aneurysm treatment

o Coiling **o** Clipping **o** Other method ________________

When was the treatment conducted?

Date _____________ DD MM Year

After admission and before the intervention has there been evidence of a re-bleeding?

□ Yes □ No

Randomization

Randomization (treatment group) ?

o Lumbar Drain (LD)

o No lumbar drain (NoLD)

Randomization number □□□□

Control head CT after aneurysm treatment

(Time frame: 6 hours until day 1 after treatment)

Date _____________ DD MM Year

Ischemic infarction newly occurred after intervention? **o** yes **o** no

Bleeding newly occurred after intervention? **o** yes **o** no

Name of physician (capital letters, please) ____________________________

Date / Signature ____________________________

EARLYdrain - Visitation 2 – Daily visitations
Pat.ID □□□ Study Center ID □□□ Date_____________

Day 1 after aneurysm treatment 0:00 – 23:59 / page 1

Blood pressure at 7:00 a.m. (syst/diast) _____ / _____ mmHg

Have catecholamines been administered on this day?
□ No □ Noradrenaline □ Dopamine □ Dobutamine □ Other

Total 24-hours fluid balance day 1: intake: __________ml output: ___________ml

Intracranial pressure recorded? □ Yes □ No

If yes:

ICP at 7.00 a.m.: _____ mmHg

Highest ICP-value observed on this day: _____ mmHg

Lowest ICP-value observed on this day: _____ mmHg

Is the patient intubated at 7:00 a.m.? □ Yes □ No

Is the patient sedated at 7:00 a.m.? □ Yes □ No

Did the patient receive a blood transfusion on treatment day 1? □ Yes □ No

Hemoglobin (~7:00 a.m.) _____ g/dl

Highest body temperature observed _____ °C

Neuromonitoring:

Mean blood flow velocity in MCA:

Right MCA _______________cm/s

Left MCA _______________cm/s

Is there clinical evidence for vasospasm?

□ Yes □ No □ Cannot be determined due to sedation

EARLYdrain - Visitation 2 – Daily visitations
Pat.ID □□□ Study Center ID □□□ Date_____________

Day 1 after aneurysm treatment 0:00 – 23:59 / page 2

Has further neuromonitoring been carried out?

□ Microdialysis □ Jugular bulb oximetry
□ Cerebral blood flow □ Brain tissue oxygenation $p_{br}O_2$ recording
□ ....

Ventricular drain in place?

□ Yes □ No

If yes: Amount of ventricular drainage on this day: ______ ml/d

Treatment with Lumbar drainage:

(if not applicable please skip next page)

Amount of lumbar drainage on this day ___ml/d

Has the lumbar drainage been discontinued for more than 1 hour?

□ Yes □ No

If yes, why?

□ LD clinically not indicated □ Risk of LD considered too high

□ accidental over-drainage □ ....

Has the lumbar drain been opened again?

□ Yes □ No

In case of a necessary discontinuation of the LD for > 1 hour:

Has a control head CT been done within 24 hours after discontinuation of the drain?

□ Yes □ No

If yes, please give a summary of the radiological CT-report:

....
....
....

Day 1 after aneurysm treatment 0:00 – 23:59 / page 3

Adverse events (AE) / Severe adverse events (SAE)

SAE present according to protocol? □Yes □No

CAUTION !

If a severe adverse event occurs, the study center must be notified within 24 hours of discovery!

For that purpose please fill in the appropriate fax form.

CSB – Secretary
Charite Berlin Campus Mitte
Chariteplatz 1
10117 Berlin
Germany
Phone: +49 30 xxx xxx xxx
Fax: +49 30 xxx xxx xxx

Name of physician (capital letters, please) ______________________________

Date / Signature ______________________________

EARLYdrain - Visitation 2 – Daily visitations
Pat.ID □□□ Study Center ID □□□ Date_____________

Day 2 after aneurysm treatment 0:00 – 23:59 / page 1

Blood pressure at 7:00 a.m. (syst/diast) _____ / _____ mmHg

Have catecholamines been administered on this day?
□ No □ Noradrenaline □ Dopamine □ Dobutamine □ Other

Total 24-hours fluid balance day 1: intake: __________ml output: ___________ml

Intracranial pressure recorded? □ Yes □ No

If yes:

ICP at 7.00 a.m.: _____ mmHg

Highest ICP-value observed on this day: _____ mmHg

Lowest ICP-value observed on this day: _____ mmHg

Is the patient intubated at 7:00 a.m.? □ Yes □ No

Is the patient sedated at 7:00 a.m.? □ Yes □ No

Did the patient receive a blood transfusion on treatment day 1? □ Yes □ No

Hemoglobin (~7:00 a.m.) _____ g/dl

Highest body temperature observed _____ °C

Neuromonitoring:

Mean blood flow velocity in MCA:

Right MCA _______________cm/s

Left MCA _______________cm/s

Is there clinical evidence for vasospasm?

□ Yes □ No □ Cannot be determined due to sedation

EARLYdrain - Visitation 2 – Daily visitations
Pat.ID □□□ Study Center ID □□□ Date_____________

Day 2 after aneurysm treatment 0:00 – 23:59 / page 2

Has further neuromonitoring been carried out?

□ Microdialysis □ Jugular bulb oximetry
□ Cerebral blood flow □ Brain tissue oxygenation $p_{br}O_2$ recording
□ ....

Ventricular drain in place?

□ Yes □ No

If yes: Amount of ventricular drainage on this day: ______ ml/d

Treatment with Lumbar drainage:

(if not applicable please skip next page)

Amount of lumbar drainage on this day ___ml/d

Has the lumbar drainage been discontinued for more than 1 hour?

□ Yes □ No

If yes, why?

□ LD clinically not indicated □ Risk of LD considered too high

□ accidental over-drainage □ ....

Has the lumbar drain been opened again?

□ Yes □ No

In case of a necessary discontinuation of the LD for > 1 hour:

Has a control head CT been done within 24 hours after discontinuation of the drain?

□ Yes □ No

If yes, please give a summary of the radiological CT-report:

....
....
....

Pat.ID □□□ Study Center ID □□□ Date______________

Day 2 after aneurysm treatment 0:00 – 23:59 / page 3

Adverse events (AE) / Severe adverse events (SAE)

SAE present according to protocol? □Yes □No

CAUTION !

If a severe adverse event occurs, the study center must be notified within 24 hours of discovery!

For that purpose please fill in the appropriate fax form.

CSB – Secretary
Charite Berlin Campus Mitte
Chariteplatz 1
10117 Berlin
Germany
Phone: +49 30 xxx xxx xxx
Fax: +49 30 xxx xxx xxx

Name of physician (capital letters, please) ________________________________

Date / Signature ________________________________

EARLYdrain - Visitation 2 – Daily visitations
Pat.ID □□□ Study Center ID □□□ Date_____________

Day 3 after aneurysm treatment 0:00 – 23:59 / page 1

Blood pressure at 7:00 a.m. (syst/diast) _____ / _____ mmHg

Have catecholamines been administered on this day?
□ No □ Noradrenaline □ Dopamine □ Dobutamine □ Other

Total 24-hours fluid balance day 1: intake: __________ml output: ___________ml

Intracranial pressure recorded? □ Yes □ No

If yes:

ICP at 7.00 a.m.: _____ mmHg

Highest ICP-value observed on this day: _____ mmHg

Lowest ICP-value observed on this day: _____ mmHg

Is the patient intubated at 7:00 a.m.? □ Yes □ No

Is the patient sedated at 7:00 a.m.? □ Yes □ No

Did the patient receive a blood transfusion on treatment day 1? □ Yes □ No

Hemoglobin (~7:00 a.m.) _____ g/dl

Highest body temperature observed _____ °C

Neuromonitoring:

Mean blood flow velocity in MCA:

Right MCA _______________cm/s

Left MCA _______________cm/s

Is there clinical evidence for vasospasm?

□ Yes □ No □ Cannot be determined due to sedation

Day 3 after aneurysm treatment 0:00 – 23:59 / page 2

Has further neuromonitoring been carried out?

□ Microdialysis □ Jugular bulb oximetry
□ Cerebral blood flow □ Brain tissue oxygenation $p_{br}O_2$ recording
□ ....

Ventricular drain in place?

□ Yes □ No

If yes: Amount of ventricular drainage on this day: ______ ml/d

Treatment with Lumbar drainage:

(if not applicable please skip next page)

Amount of lumbar drainage on this day ___ml/d

Has the lumbar drainage been discontinued for more than 1 hour?

□ Yes □ No

If yes, why?

□ LD clinically not indicated □ Risk of LD considered too high

□ accidental over-drainage □ ....

Has the lumbar drain been opened again?

□ Yes □ No

In case of a necessary discontinuation of the LD for > 1 hour:

Has a control head CT been done within 24 hours after discontinuation of the drain?

□ Yes □ No

If yes, please give a summary of the radiological CT-report:

....
....
....

EARLYdrain - Visitation 2 – Daily visitations
Pat.ID □□□ Study Center ID □□□ Date_____________

Day 3 after aneurysm treatment 0:00 – 23:59 / page 3

Adverse events (AE) / Severe adverse events (SAE)

SAE present according to protocol? □Yes □No

CAUTION !

If a severe adverse event occurs, the study center must be notified within 24 hours of discovery!

For that purpose please fill in the appropriate fax form.

CSB – Secretary
Charite Berlin Campus Mitte
Chariteplatz 1
10117 Berlin
Germany
Phone: +49 30 xxx xxx xxx
Fax: +49 30 xxx xxx xxx

Name of physician (capital letters, please) ______________________________

Date / Signature ______________________________

EARLYdrain - Visitation 2 – Daily visitations
Pat.ID □□□ Study Center ID □□□ Date_____________

Day 4 after aneurysm treatment 0:00 – 23:59 / page 1

Blood pressure at 7:00 a.m. (syst/diast) _____ / _____ mmHg

Have catecholamines been administered on this day?
□ No □ Noradrenaline □ Dopamine □ Dobutamine □ Other

Total 24-hours fluid balance day 1: intake: __________ml output: ___________ml

Intracranial pressure recorded? □ Yes □ No

If yes:

ICP at 7.00 a.m.: _____ mmHg

Highest ICP-value observed on this day: _____ mmHg

Lowest ICP-value observed on this day: _____ mmHg

Is the patient intubated at 7:00 a.m.? □ Yes □ No

Is the patient sedated at 7:00 a.m.? □ Yes □ No

Did the patient receive a blood transfusion on treatment day 1? □ Yes □ No

Hemoglobin (~7:00 a.m.) _____ g/dl

Highest body temperature observed _____ °C

Neuromonitoring:

Mean blood flow velocity in MCA:

Right MCA _______________cm/s

Left MCA _______________cm/s

Is there clinical evidence for vasospasm?

□ Yes □ No □ Cannot be determined due to sedation

EARLYdrain - Visitation 2 – Daily visitations
Pat.ID □□□ Study Center ID □□□ Date_____________

Day 4 after aneurysm treatment 0:00 – 23:59 / page 2

Has further neuromonitoring been carried out?

□ Microdialysis □ Jugular bulb oximetry
□ Cerebral blood flow □ Brain tissue oxygenation $p_{br}O_2$ recording
□ ....

Ventricular drain in place?

□ Yes □ No

If yes: Amount of ventricular drainage on this day: ______ ml/d

Treatment with Lumbar drainage:

(if not applicable please skip next page)

Amount of lumbar drainage on this day ___ml/d

Has the lumbar drainage been discontinued for more than 1 hour?

□ Yes □ No

If yes, why?

□ LD clinically not indicated □ Risk of LD considered too high

□ accidental over-drainage □ ....

Has the lumbar drain been opened again?

□ Yes □ No

In case of a necessary discontinuation of the LD for > 1 hour:

Has a control head CT been done within 24 hours after discontinuation of the drain?

□ Yes □ No

If yes, please give a summary of the radiological CT-report:

....
....
....

EARLYdrain - Visitation 2 – Daily visitations
Pat.ID □□□ Study Center ID □□□ Date_____________

Day 4 after aneurysm treatment 0:00 – 23:59 / page 3

Adverse events (AE) / Severe adverse events (SAE)

SAE present according to protocol? □Yes □No

CAUTION !

If a severe adverse event occurs, the study center must be notified within 24 hours of discovery!

For that purpose please fill in the appropriate fax form.

CSB – Secretary
Charite Berlin Campus Mitte
Chariteplatz 1
10117 Berlin
Germany
Phone: +49 30 xxx xxx xxx
Fax: +49 30 xxx xxx xxx

Name of physician (capital letters, please) ______________________________

Date / Signature ______________________________

EARLYdrain - Visitation 2 – Daily visitations
Pat.ID □□□ Study Center ID □□□ Date_____________

Day 5 after aneurysm treatment 0:00 – 23:59 / page 1

Blood pressure at 7:00 a.m. (syst/diast) _____ / _____ mmHg

Have catecholamines been administered on this day?
□ No □ Noradrenaline □ Dopamine □ Dobutamine □ Other

Total 24-hours fluid balance day 1: intake: __________ml output: ___________ml

Intracranial pressure recorded? □ Yes □ No

If yes:

ICP at 7.00 a.m.: _____ mmHg

Highest ICP-value observed on this day: _____ mmHg

Lowest ICP-value observed on this day: _____ mmHg

Is the patient intubated at 7:00 a.m.? □ Yes □ No

Is the patient sedated at 7:00 a.m.? □ Yes □ No

Did the patient receive a blood transfusion on treatment day 1? □ Yes □ No

Hemoglobin (~7:00 a.m.) _____ g/dl

Highest body temperature observed _____ °C

Neuromonitoring:

Mean blood flow velocity in MCA:

Right MCA _______________cm/s

Left MCA _______________cm/s

Is there clinical evidence for vasospasm?

□ Yes □ No □ Cannot be determined due to sedation

EARLYdrain - Visitation 2 – Daily visitations
Pat.ID □□□ Study Center ID □□□ Date_____________

Day 5 after aneurysm treatment 0:00 – 23:59 / page 2

Has further neuromonitoring been carried out?

□ Microdialysis □ Jugular bulb oximetry
□ Cerebral blood flow □ Brain tissue oxygenation $p_{br}O_2$ recording
□ ....

Ventricular drain in place?

□ Yes □ No

If yes: Amount of ventricular drainage on this day: ______ ml/d

Treatment with Lumbar drainage:

(if not applicable please skip next page)

Amount of lumbar drainage on this day ___ml/d

Has the lumbar drainage been discontinued for more than 1 hour?

□ Yes □ No

If yes, why?

□ LD clinically not indicated □ Risk of LD considered too high

□ accidental over-drainage □ ....

Has the lumbar drain been opened again?

□ Yes □ No

In case of a necessary discontinuation of the LD for > 1 hour:

Has a control head CT been done within 24 hours after discontinuation of the drain?

□ Yes □ No

If yes, please give a summary of the radiological CT-report:

....
....
....

Day 5 after aneurysm treatment 0:00 – 23:59 / page 3

Adverse events (AE) / Severe adverse events (SAE)

SAE present according to protocol? □Yes □No

CAUTION !

If a severe adverse event occurs, the study center must be notified within 24 hours of discovery!

For that purpose please fill in the appropriate fax form.

CSB – Secretary
Charite Berlin Campus Mitte
Chariteplatz 1
10117 Berlin
Germany
Phone: +49 30 xxx xxx xxx
Fax: +49 30 xxx xxx xxx

Name of physician (capital letters, please) ________________________________

Date / Signature ________________________________

EARLYdrain - Visitation 2 – Daily visitations
Pat.ID □□□ Study Center ID □□□ Date_____________

Day 6 after aneurysm treatment 0:00 – 23:59 / page 1

Blood pressure at 7:00 a.m. (syst/diast) _____ / _____ mmHg

Have catecholamines been administered on this day?
□ No □ Noradrenaline □ Dopamine □ Dobutamine □ Other

Total 24-hours fluid balance day 1: intake: __________ml output: ___________ml

Intracranial pressure recorded? □ Yes □ No

If yes:

ICP at 7.00 a.m.: _____ mmHg

Highest ICP-value observed on this day: _____ mmHg

Lowest ICP-value observed on this day: _____ mmHg

Is the patient intubated at 7:00 a.m.? □ Yes □ No

Is the patient sedated at 7:00 a.m.? □ Yes □ No

Did the patient receive a blood transfusion on treatment day 1? □ Yes □ No

Hemoglobin (~7:00 a.m.) _____ g/dl

Highest body temperature observed _____ °C

Neuromonitoring:

Mean blood flow velocity in MCA:

Right MCA _______________cm/s

Left MCA _______________cm/s

Is there clinical evidence for vasospasm?

□ Yes □ No □ Cannot be determined due to sedation

EARLYdrain - Visitation 2 – Daily visitations
Pat.ID □□□ Study Center ID □□□ Date_____________

Day 6 after aneurysm treatment 0:00 – 23:59 / page 2

Has further neuromonitoring been carried out?

□ Microdialysis
□ Jugular bulb oximetry
□ Cerebral blood flow
□ Brain tissue oxygenation $p_{br}O_2$ recording
□ ....

Ventricular drain in place?

□ Yes □ No

If yes: Amount of ventricular drainage on this day: ______ ml/d

Treatment with Lumbar drainage:

(if not applicable please skip next page)

Amount of lumbar drainage on this day ___ml/d

Has the lumbar drainage been discontinued for more than 1 hour?

□ Yes □ No

If yes, why?

□ LD clinically not indicated
□ Risk of LD considered too high
□ accidental over-drainage
□ ....

Has the lumbar drain been opened again?

□ Yes □ No

In case of a necessary discontinuation of the LD for > 1 hour:

Has a control head CT been done within 24 hours after discontinuation of the drain?

□ Yes □ No

If yes, please give a summary of the radiological CT-report:

....
....
....

EARLYdrain - Visitation 2 – Daily visitations
Pat.ID □□□ Study Center ID □□□ Date_______________

Day 6 after aneurysm treatment 0:00 – 23:59 / page 3

Adverse events (AE) / Severe adverse events (SAE)

SAE present according to protocol? □Yes □No

CAUTION !

If a severe adverse event occurs, the study center must be notified within 24 hours of discovery!

For that purpose please fill in the appropriate fax form.

CSB – Secretary
Charite Berlin Campus Mitte
Chariteplatz 1
10117 Berlin
Germany
Phone: +49 30 xxx xxx xxx
Fax: +49 30 xxx xxx xxx

Name of physician (capital letters, please) ________________________________

Date / Signature ________________________________

EARLYdrain - Visitation 2 – Daily visitations
Pat.ID □□□ Study Center ID □□□ Date_____________

Day 7 after aneurysm treatment 0:00 – 23:59 / page 1

Blood pressure at 7:00 a.m. (syst/diast) _____ / _____ mmHg

Have catecholamines been administered on this day?
□ No □ Noradrenaline □ Dopamine □ Dobutamine □ Other

Total 24-hours fluid balance day 1: intake: __________ml output: ___________ml

Intracranial pressure recorded? □ Yes □ No

If yes:

ICP at 7.00 a.m.: _____ mmHg

Highest ICP-value observed on this day: _____ mmHg

Lowest ICP-value observed on this day: _____ mmHg

Is the patient intubated at 7:00 a.m.? □ Yes □ No

Is the patient sedated at 7:00 a.m.? □ Yes □ No

Did the patient receive a blood transfusion on treatment day 1? □ Yes □ No

Hemoglobin (~7:00 a.m.) _____ g/dl

Highest body temperature observed _____ °C

Neuromonitoring:

Mean blood flow velocity in MCA:

Right MCA _______________cm/s

Left MCA _______________cm/s

Is there clinical evidence for vasospasm?

□ Yes □ No □ Cannot be determined due to sedation

EARLYdrain - Visitation 2 – Daily visitations
Pat.ID □□□ Study Center ID □□□ Date_____________

Day 7 after aneurysm treatment 0:00 – 23:59 / page 2

Has further neuromonitoring been carried out?

□ Microdialysis □ Jugular bulb oximetry
□ Cerebral blood flow □ Brain tissue oxygenation $p_{br}O_2$ recording
□ ....

Ventricular drain in place?

□ Yes □ No

If yes: Amount of ventricular drainage on this day: ______ ml/d

Treatment with Lumbar drainage:

(if not applicable please skip next page)

Amount of lumbar drainage on this day ___ml/d

Has the lumbar drainage been discontinued for more than 1 hour?

□ Yes □ No

If yes, why?

□ LD clinically not indicated □ Risk of LD considered too high

□ accidental over-drainage □ ....

Has the lumbar drain been opened again?

□ Yes □ No

In case of a necessary discontinuation of the LD for > 1 hour:

Has a control head CT been done within 24 hours after discontinuation of the drain?

□ Yes □ No

If yes, please give a summary of the radiological CT-report:

....
....
....

EARLYdrain - Visitation 2 – Daily visitations
Pat.ID □□□ Study Center ID □□□ Date_____________

Day 7 after aneurysm treatment 0:00 – 23:59 / page 3

Adverse events (AE) / Severe adverse events (SAE)

SAE present according to protocol? □Yes □No

CAUTION !

If a severe adverse event occurs, the study center must be notified within 24 hours of discovery!

For that purpose please fill in the appropriate fax form.

CSB – Secretary
Charite Berlin Campus Mitte
Chariteplatz 1
10117 Berlin
Germany
Phone: +49 30 xxx xxx xxx
Fax: +49 30 xxx xxx xxx

Name of physician (capital letters, please) ______________________________

Date / Signature ______________________________

EARLYdrain - Visitation 2 – Daily visitations
Pat.ID □□□ Study Center ID □□□ Date_____________

Day 8 after aneurysm treatment 0:00 – 23:59 / page 1

Blood pressure at 7:00 a.m. (syst/diast) _____ / _____ mmHg

Have catecholamines been administered on this day?
□ No □ Noradrenaline □ Dopamine □ Dobutamine □ Other

Total 24-hours fluid balance day 1: intake: __________ml output: ___________ml

Intracranial pressure recorded? □ Yes □ No

If yes:

ICP at 7.00 a.m.: _____ mmHg

Highest ICP-value observed on this day: _____ mmHg

Lowest ICP-value observed on this day: _____ mmHg

Is the patient intubated at 7:00 a.m.? □ Yes □ No

Is the patient sedated at 7:00 a.m.? □ Yes □ No

Did the patient receive a blood transfusion on treatment day 1? □ Yes □ No

Hemoglobin (~7:00 a.m.) _____ g/dl

Highest body temperature observed _____ °C

Neuromonitoring:

Mean blood flow velocity in MCA:

Right MCA _______________cm/s

Left MCA _______________cm/s

Is there clinical evidence for vasospasm?

□ Yes □ No □ Cannot be determined due to sedation

EARLYdrain - Visitation 2 – Daily visitations
Pat.ID □□□ Study Center ID □□□ Date_____________

Day 8 after aneurysm treatment 0:00 – 23:59 / page 2

Has further neuromonitoring been carried out?

□ Microdialysis □ Jugular bulb oximetry
□ Cerebral blood flow □ Brain tissue oxygenation $p_{br}O_2$ recording
□ ....

Ventricular drain in place?

□ Yes □ No

If yes: Amount of ventricular drainage on this day: ______ ml/d

Treatment with Lumbar drainage:

(if not applicable please skip next page)

Amount of lumbar drainage on this day ___ml/d

Has the lumbar drainage been discontinued for more than 1 hour?

□ Yes □ No

If yes, why?

□ LD clinically not indicated □ Risk of LD considered too high

□ accidental over-drainage □ ....

Has the lumbar drain been opened again?

□ Yes □ No

In case of a necessary discontinuation of the LD for > 1 hour:

Has a control head CT been done within 24 hours after discontinuation of the drain?

□ Yes □ No

If yes, please give a summary of the radiological CT-report:

....
....
....

EARLYdrain - Visitation 2 – Daily visitations
Pat.ID □□□ Study Center ID □□□ Date_____________

Day 8 after aneurysm treatment 0:00 – 23:59 / page 3

Adverse events (AE) / Severe adverse events (SAE)

SAE present according to protocol? □Yes □No

CAUTION !

If a severe adverse event occurs, the study center must be notified within 24 hours of discovery!

For that purpose please fill in the appropriate fax form.

CSB – Secretary
Charite Berlin Campus Mitte
Chariteplatz 1
10117 Berlin
Germany
Phone: +49 30 xxx xxx xxx
Fax: +49 30 xxx xxx xxx

Name of physician (capital letters, please) ______________________________

Date / Signature ______________________________

EARLYdrain - Visitation 3 – Discharge
Pat.ID □□□ Study Center ID □□□ Date_____________

Discharge

When was the patient transferred or discharged?

Date ______________ DD MM YYYY

Where to?

o Other acute care clinic
o Rehabilitation clinic
o Home
o Local hospital
o Other type of establishment
o Patient deceased

Was the patient treated according to randomisation?

LD-Group: At least 4 days of lumbar drainage with 120 ml/day

No-LD-Group: No lumbar drainage for the first 8 days after intervention

□ Yes □ No

If no, why not?

....
....
....

During the patient's stay were there parameters of infections correspondent to CDC criteria?

□ Yes □ No

One of the following symptoms without other cause evident:
Fever (>38°C), headache, stiff neck, Meningismus, cranial nerve symptoms and irritability
AND
At least one of the following criteria:
Cultural detection of pathogens in the CSF
Elevated white blood cell count, increased protein content and/or decreased glucose in CSF
Microscopic detection of microorganisms in cerebrospinal fluid, cultural detection of pathogens in the blood, positive antigen detection in cerebrospinal fluid, blood or urine, diagnostic single antibody titer (IgM) or fourfold increase in titer (IgG) in repeatedly extracted serum samples for the pathogens in question.

EARLYdrain - Visitation 3 – Discharge
Pat.ID □□□ Study Center ID □□□ Date_____________

Adverse events (AE) / Severe adverse events (SAE)

SAE present according to protocol? □Yes □No

CAUTION !

If a severe adverse event occurs, the study center must be notified within 24 hours of discovery!
For that purpose please fill in the appropriate fax form.

Modified Rankin Scale (mRS) at discharge

0 – no symptoms

1 - No significant disability despite some symptoms; but able to carry out all usual duties and activities

2 - Slight disability; unable to carry out all previous activities, but able to look after own affairs without assistance

3 – moderate disability; requiring some help, but able to walk without assistance

4 – moderate severe disability; unable to walk without assistance and unable to attend to own bodily needs without assistance

5 – Severe disability; bedridden, incontinent and requiring constant nursing care and attention

6 – Deceased

Please add a brief written statement:

....
....
....

EARLYdrain - Visitation 3 – Discharge
Pat.ID □□□ Study Center ID □□□ Date_____________

Imaging

Vascular control on day 7 to 10 after aneurysm

When was the control angiography performed?

Date ______________ DD MM YYYY

Type of Angiography (Multiple answers possible)

o DSA **o** MRA **o** CTA **o** not performed

Could a vasospasm be detected?

□ Yes □ No

If yes, how much was the lumen of the most affected vessel reduced by?

o up to 33% **o** up to 66% **o** more than 66%

If yes, was any endovascular rescue therapy performed?

o no **o** percutaneous transluminal angioplasty **o** intraarterial vasodilator

Type of final imaging performed before discharge:

o CCT **o** MRT

Performed on:

Date ______________ DD MM YYYY

Compared to the imaging from day one (postoperative/ post-interventional imaging control), are there new infarctions visible?

□ Yes □ No

EARLYdrain - Visitation 3 – Discharge
Pat.ID □□□ Study Center ID □□□ Date_____________

Medication and clinical questions

Was the following medication given to the patient during his hospital stay for vasospasm prophylaxis or therapy?

Was Nimodipine given? □ Yes □ No

Were statins given? □ Yes □ No

Was Mg++ given? □ Yes □ No

Was Milrinone given? □ Yes □ No

Was an antimicrobially coated LD used? □ Yes □ No

Was an antimicrobially coated EVD used? □ Yes □ No

At any time during the hospital stay, was there an indication of a clinical vasospasm?

□ Yes □ No

Was a ventriculo-peritoneal (or other) shunt implanted?

□ Yes □ No

If yes, when:

Date ______________ DD MM YYYY

EARLYdrain - Visitation 3 – Discharge
Pat.ID □□□ Study Center ID □□□ Date_____________

Glasgow-Outcome-Scale und extended Glasgow-Outcome-Scale

Please check the main GOS category as well as the sub-categories for GOS 3-5. If uncertain about assessment please contact the study center

□ **GOS-5: Good recovery**

- □ upper good recovery
  Patient has no residual symptoms and is able to have the same quality of life as before the SAH, for example, is able to resume work

- □ lower good recovery
  Patient has residual symptoms but is able to have almost the same quality of life as before the SAH and is able to resume at least 50% of social and past-time activities

□ **GOS-4: Moderate Disability, requires no assistance in daily life**

- □ upper moderate disability
  Patient is limited in his ability to work and/or picks less than 50% of previous past-time activities back up and/or has occasional but noticeable behavioral problems

- □ lower moderate disability
  Patient can only work in a supervised establishment or is not able to work at all and/or is barely or not at all able to participate in family events or past-time activities and/or has daily, severe and intolerable behavioral problems

□ **GOS-3: Severe disability requiring help in daily life**

- □ upper severe disability
  Patient can be left unsupervised for at least 8 hours during the day but is not able to move independently in daily life or go going shopping on his own.

- □ lower severe disability
  Patient requires help frequently or is dependent on permanent care

□ **GOS-2: Persistent vegetative state**

Patient is unresponsive and without speech, may have open eyes and / or sleep/wake cycles

□ **GOS-1: Deceased**

EARLYdrain - Visitation 3 – Discharge
Pat.ID □□□ Study Center ID □□□ Date____________

Early Rehabilitation Index at discharge

	Yes / Points	No / Points
Requires intensive care monitoring (eg vegetative crises)	o - 50	o 0
Tracheostomy requiring suction	o - 50	o 0
intermittent artificial respiration necessary	o - 50	o 0
Patient disoriented/confused, requiring observation	o - 50	o 0
Behavioral problems requiring observation (danger to self or others)	o - 50	o 0
Severe comprehension / communication problems	o - 25	o 0
Dysphagia requiring observation	o - 50	o 0
**Total**		

**Barthel Index at discharge / Part 1**	**Points**
**Eating**	
Independent, eats independently, uses cutlery	o10
Requires some assistance e.g. cutting meats or bread	o 5
Not independent even with help as listed above	o 0
**Bed- (Wheelchair-) Transfer**	
Independent without assistance	o 10
With assistance	o 5
Not possible	o 0
**Personal hygiene**	
Independently washes face, brushes hair and teeth	o 5
Cannot perform activities listed above independently	o 0
**Use of facilities**	
Independent (sitting down, standing up, undressing and dressing and wiping)	o 10
Requires some assistance, but is able to complete some tasks independently	o 5
Not independent, even with above assistance	o 0
**Bathing**	
Able to shower or bathe completely independent	o 5
Cannot shower or bathe independently	o 0

EARLYdrain - Visitation 3 – Discharge
Pat.ID □□□ Study Center ID □□□ Date_____________

**Barthel Index at discharge / Part 2**	**Points**
**Walking/ Use of a wheelchair**	
Able to walk independently over 50 m, assistive equipment allowed, no walker	o 15
Requires some assistance or supervision, is able to walk 50 m with assistive equipment or walker	o 10
Cannot walk independently, able to use a wheelchair independently, even around corners and at the table or bed	o 5
Cannot walk or use wheelchair independently	o 0
**Climbing stairs**	
Able to climb stairs (several steps)	o10
Requires assistance or supervision while climbing stairs	o 5
Not independent, not able to climb stairs, even with assistance	o 0
**Dressing and undressing**	
Dresses and undresses independently (if applicable including brace or truss)	o10
Requires assistance but able to perform 50% of the task independently	o 5
Not independent even if assistance listed above is given	o 0
**Bladder control**	
Constantly continent, possibly with independent supply of indwelling catheter / Cystofix	o10
Constantly continent, max. once per week incontinent	
Frequently / permanently incontinent	o 5
	o 0
**Bowel control**	
Constantly continent	o 10
Constantly continent, max. once / week incontinent	o 5
Frequently / permanently incontinent	o 0
	**Total**
**Total Barthel-Index**	
**Total Early Rehabilitation-Index**	
**Early Rehabilitation + Barthel-Index**	

Name of physician (capital letters, please) ______________________________

Date / Signature ______________________________

EARLYdrain – Visitation 4 – 180 Days (6 months) after inclusion
Pat.ID □□□ Study Center ID □□□ Date_____________

VISITATION 4 – Final assessment

Who was interviewed during this visitation?

Patient □Yes
Relative □Yes
Nursing staff □Yes
Legal guardian □Yes
Station / primary physician □Yes

Since the last visit, was the patient hospitalized in an acute care hospital?

□ Yes □ No

If yes, reason:

____

The patient continues to be/ is once again in rehabilitation:

□ Yes □ No

Did the patient die after being discharged for the first time (Visitation 3) but before Visitation 4 (180 Days)

□ Yes □ No

If yes, when?

Date ______________ DD MM YYYY

If yes, cause of death:

____

Adverse events (AE) / Severe adverse events (SAE)

SAE present according to protocol? □Yes □No

CAUTION !

If a severe adverse event occurs, the study center must be notified within 24 hours of discovery!
For that purpose please fill in the appropriate fax form.

EARLYdrain – Visitation 4 – 180 Days (6 months) after inclusion
Pat.ID □□□ Study Center ID □□□ Date_____________

Modified Rankin Scale (mRS) on Day 180

0 – no symptoms

1 - No significant disability despite some symptoms; but able to carry out all usual duties and activities

2 - Slight disability; unable to carry out all previous activities, but able to look after own affairs without assistance

3 – moderate disability; requiring some help, but able to walk without assistance

4 – moderate severe disability; unable to walk without assistance and unable to attend to own bodily needs without assistance

5 – Severe disability; bedridden, incontinent and requiring constant nursing care and attention

6 – Deceased

Was a ventriculo-peritoneal (or other) shunt inserted after the initial discharge (visitation 3) but before the final assessment (visitation 4)?

□ Yes □ No

If yes, when:

Date ______________ DD MM YYYY

EARLYdrain - Visitation 4 – 180 Days (6 months) after inclusion
Pat.ID □□□ Study Center ID □□□ Date_____________

Glasgow-Outcome-Scale und extended Glasgow-Outcome-Scale at day 180

Please check the main GOS category as well as the sub-categories for GOS 3-5. If uncertain about assessment please contact the study center

□ **GOS-5: Good recovery**

- □ upper good recovery
  Patient has no residual symptoms and is able to have the same quality of life as before the SAH, for example, is able to resume work

- □ lower good recovery
  Patient has residual symptoms but is able to have almost the same quality of life as before the SAH and is able to resume at least 50% of social and past-time activities

□ **GOS-4: Moderate Disability, requires no assistance in daily life**

- □ upper moderate disability
  Patient is limited in his ability to work and/or picks less than 50% of previous past-time activities back up and/or has occasional but noticeable behavioral problems

- □ lower moderate disability
  Patient can only work in a supervised establishment or is not able to work at all and/or is barely or not at all able to participate in family events or past-time activities and/or has daily, severe and intolerable behavioral problems

□ **GOS-3: Severe disability requiring help in daily life**

- □ upper severe disability
  Patient can be left unsupervised for at least 8 hours during the day but is not able to move independently in daily life or go going shopping on his own.

- □ lower severe disability
  Patient requires help frequently or is dependent on permanent care

□ **GOS-2: Persistent vegetative state**

Patient is unresponsive and without speech, may have open eyes and / or sleep/wake cycles

□ **GOS-1: Deceased**

EARLYdrain - Visitation 4 – 180 Days (6 months) after inclusion
Pat.ID □□□ Study Center ID □□□ Date_____________

Early Rehabilitation Index at day 180

	Yes / Points	No / Points
Requires intensive care monitoring (eg vegetative crises)	o - 50	o 0
Tracheostomy requiring suction	o - 50	o 0
intermittent artificial respiration necessary	o - 50	o 0
Patient disoriented/confused, requiring observation	o - 50	o 0
Behavioral problems requiring observation (danger to self or others)	o - 50	o 0
Severe comprehension / communication problems	o - 25	o 0
Dysphagia requiring observation	o - 50	o 0
**Total**		

**Barthel Index at day 180 / Part 1**	**Points**
**Eating**	
Independent, eats independently, uses cutlery	o10
Requires some assistance e.g. cutting meats or bread	o 5
Not independent even with help as listed above	o 0
**Bed- (Wheelchair-) Transfer**	
Independent without assistance	o 10
With assistance	o 5
Not possible	o 0
**Personal hygiene**	
Independently washes face, brushes hair and teeth	o 5
Cannot perform activities listed above independently	o 0
**Use of facilities**	
Independent (sitting down, standing up, undressing and dressing and wiping)	o 10
Requires some assistance, but is able to complete some tasks independently	o 5
Not independent, even with above assistance	o 0
**Bathing**	
Able to shower or bathe completely independent	o 5
Cannot shower or bathe independently	o 0

EARLYdrain - Visitation 4 – 180 Days (6 months) after inclusion
Pat.ID □□□ Study Center ID □□□ Date_____________

**Barthel Index at day 180 / Part 2**	**Points**
**Walking/ Use of a wheelchair**	
Able to walk independently over 50 m, assistive equipment allowed, no walker	o 15
Requires some assistance or supervision, is able to walk 50 m with assistive equipment or walker	o 10
Cannot walk independently, able to use a wheelchair independently, even around corners and at the table or bed	o 5
Cannot walk or use wheelchair independently	o 0
**Climbing stairs**	
Able to climb stairs (several steps)	o10
Requires assistance or supervision while climbing stairs	o 5
Not independent, not able to climb stairs, even with assistance	o 0
**Dressing and undressing**	
Dresses and undresses independently (if applicable including brace or truss)	o10
Requires assistance but able to perform 50% of the task independently	o 5
Not independent even if assistance listed above is given	o 0
**Bladder control**	
Constantly continent, possibly with independent supply of indwelling catheter / Cystofix	o10
Constantly continent, max. once per week incontinent	
Frequently / permanently incontinent	o 5
	o 0
**Bowel control**	
Constantly continent	o 10
Constantly continent, max. once / week incontinent	o 5
Frequently / permanently incontinent	o 0
	**Total**
**Total Barthel-Index**	
**Total Early Rehabilitation-Index**	
**Early Rehabilitation + Barthel-Index**	

Name of physician (capital letters, please) ______________________________

Date / Signature ______________________________
