# Supplementary material for: Effectiveness of Lumbar Cerebrospinal Fluid Drain Among Patients With Aneurysmal Subarachnoid Hemorrhage: A Randomized Clinical Trial
Source: JAMA Neurol. 2023 Jun 18;80(8):833–42. doi: 10.1001/jamaneurol.2023.1792 (PMC10277935; doi:10.1001/jamaneurol.2023.1792)
Supplement: Supplement 2. — eAppendix 1. Crossover Patients eAppendix 2. Per-Protocol Analysis eAppendix 3. As-Treated Analysis eAppendix 4. Adverse Events eTable 1. Inclusion and Exclusion Criteria eTable 2. Reasons Provided for Missed Recruitments eTable 3. Clinical Data, Intention-to-Treat Analysis eTable 4. Univariate Analysis of Clinical Risk Factors for Secondary Infarctions eTable 5. Clinical and Radiological Risk Factors for Development of Secondary Infarctions, Stratified Per Factor Level eTable 6. Vasospasm Assessment According to Clinical Estimation and Infarction at Discharge eTable 7. Vasospasm Assessment by TCD and Infarctions at Discharge eTable 8. Angiographic Vasospasm and Infarctions at Discharge eTable 9. Infarctions at Discharge and mRS Score at 6 Months eTable 10. Univariate Analysis of Factors Considered for Outcome Adjustment in Intention-to-Treat Data eTable 11. Causes of Death in the Lumbar Drain Group and in the Standard of Care Group eTable 12. Baseline Characteristics of Crossover Patients Compared With Patients Treated Per Protocol eTable 13. Clinical Treatment Data in Crossover Patients eTable 14. Demographic Comparison Between Groups in Patients Treated Per Protocol eTable 15. Clinical Treatment Data in Patients Treated Per Protocol eTable 16. Univariate Analysis of Factors Considered for Outcome Adjustment in the Per-Protocol Data eTable 17. Demographic Comparison Between Groups As Actually Treated eTable 18. Clinical Treatment Data Between Groups As Actually Treated eTable 19. Univariate Analysis of Factors Considered for Outcome Adjustment in the As-Treated Data eTable 20. Univariate Analysis of Potential Factors for the Development of Infection in the Clinical Course eTable 21. Multivariate Analysis of Factors Associated With the Development of Infection eFigure 1. Drip Chambers of an EVD and a Lumbar Drain eFigure 2. Time Flow of Randomizations eFigure 3. Daily Mean Arterial Pressure eFigure 4. ICP at 7 AM eFigure 5. Highest ICP on Each Day eFigure 6. TCD Va [file jamaneurol-e231792-s002.pdf]

Supplemental Online Content

Wolf S, Mielke D, Barner C, et al; EARLYDRAIN Study Group. Effectiveness of lumbar cerebrospinal fluid drain among patients with aneurysmal subarachnoid hemorrhage: a randomized clinical trial. *JAMA Neurol*. Published online June 18, 2023. doi:10.1001/jamaneurol.2023.1792

eAppendix 1. Crossover Patients
eAppendix 2. Per-Protocol Analysis
eAppendix 3. As-Treated Analysis
eAppendix 4. Adverse Events
eTable 1. Inclusion and Exclusion Criteria
eTable 2. Reasons Provided for Missed Recruitments
eTable 3. Clinical Data, Intention-to-Treat Analysis
eTable 4. Univariate Analysis of Clinical Risk Factors for Secondary Infarctions
eTable 5. Clinical and Radiological Risk Factors for Development of Secondary Infarctions, Stratified Per Factor Level
eTable 6. Vasospasm Assessment According to Clinical Estimation and Infarction at Discharge
eTable 7. Vasospasm Assessment by TCD and Infarctions at Discharge
eTable 8. Angiographic Vasospasm and Infarctions at Discharge
eTable 9. Infarctions at Discharge and mRS Score at 6 Months
eTable 10. Univariate Analysis of Factors Considered for Outcome Adjustment in Intention-to-Treat Data
eTable 11. Causes of Death in the Lumbar Drain Group and in the Standard of Care Group
eTable 12. Baseline Characteristics of Crossover Patients Compared With Patients Treated Per Protocol
eTable 13. Clinical Treatment Data in Crossover Patients
eTable 14. Demographic Comparison Between Groups in Patients Treated Per Protocol
eTable 15. Clinical Treatment Data in Patients Treated Per Protocol
eTable 16. Univariate Analysis of Factors Considered for Outcome Adjustment in the Per-Protocol Data
eTable 17. Demographic Comparison Between Groups As Actually Treated
eTable 18. Clinical Treatment Data Between Groups As Actually Treated
eTable 19. Univariate Analysis of Factors Considered for Outcome Adjustment in the As-Treated Data
eTable 20. Univariate Analysis of Potential Factors for the Development of Infection in the Clinical Course
eTable 21. Multivariate Analysis of Factors Associated With the Development of Infection
eFigure 1. Drip Chambers of an EVD and a Lumbar Drain
eFigure 2. Time Flow of Randomizations
eFigure 3. Daily Mean Arterial Pressure
eFigure 4. ICP at 7 AM
eFigure 5. Highest ICP on Each Day
eFigure 6. TCD Values
eFigure 7. Fluid Intake Per Day
eFigure 8. Net Fluid Balance Per Day
eFigure 9. Comparison of Fever Burden
eFigure 10. Lowest Hemoglobin Value Per Day
eFigure 11. Drainage via EVD
eFigure 12. Drainage via Lumbar Drain
eFigure 13. Cerebrospinal Fluid Drainage of External Ventricular and Lumbar Drains Combined
eFigure 14. Mortality in the Intention-to-Treat Data
eFigure 15. Outcome in Patients Younger Than and Up to Median Age of 55 Years
eFigure 16. Outcome in Patients Older Than Median Age of 55 Years
eFigure 17. Outcome in Female Patients
eFigure 18. Outcome in Male Patients
eFigure 19. Outcome in Good-Grade Patients (Hunt-Hess Grades 1 and 2)
eFigure 20. Outcome in Poor-Grade Patients (Hunt-Hess Grade 3-5)

eFigure 21. Outcome in Good-Grade Patients (WFNS Grades 1-3)
eFigure 22. Outcome in Poor-Grade Patients (WFNS Grades 4 and 5)
eFigure 23. Outcome in Patients Presenting Without Intracerebral Hemorrhage
eFigure 24. Outcome in Patients With Intracerebral Hemorrhage
eFigure 25. Outcome in Patients Without Intraventricular Hemorrhage
eFigure 26. Outcome in Patients With Intraventricular Hemorrhage
eFigure 27. Outcome in Patients With an Aneurysm of the Anterior Circulation
eFigure 28. Outcome in Patients With Posterior Circulation Aneurysms
eFigure 29. Outcome in Patients Treated With Clipping
eFigure 30. Outcome in Patients Treated With Coiling
eFigure 31. Outcome of Patients Treated Without an External Ventricular Drain
eFigure 32. Outcome in Patients Where an External Ventricular Drain was Required
eFigure 33. Outcome in Patients Treated at the 2 Largest Recruiting Centers
eFigure 34. Outcome in All Other Than the 2 Largest Recruiting Centers
eFigure 35. CONSORT Diagram for Per-Protocol Analysis
eFigure 36. Mortality Analyzed Per Protocol
eFigure 37. CONSORT Diagram for As-Treated Analysis
eFigure 38. Mortality According to Actual Treatment

This supplementary material has been provided by the authors to give readers additional information about their work.

© 2023 Wolf S et al. *JAMA Neurology*.

eFigure 1. Drip Chambers of an EVD and a Lumbar Drain

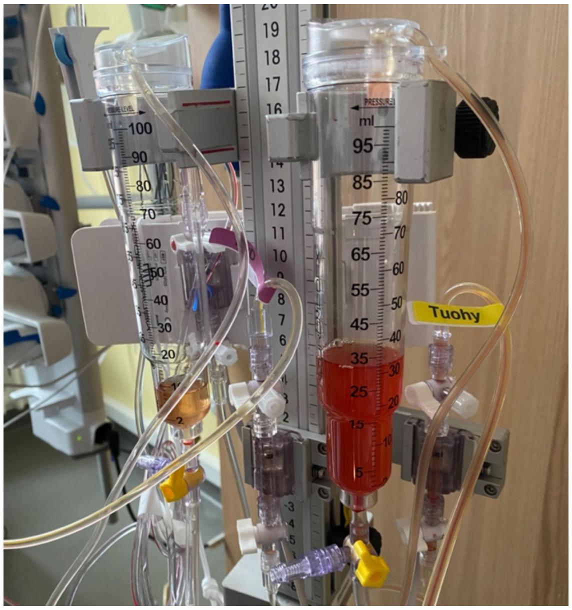

Drip chambers of an EVD (left) and a lumbar drain (right) used side by side in a patient with subarachnoid hemorrhage. Visible is the darker, more reddish staining of the cerebrospinal fluid from the lumbar drain, indicating the higher amount of blood and blood degradation products.

© 2023 Wolf S et al. *JAMA Neurology*.

eTable 1. Inclusion and Exclusion Criteria

Subject Inclusion Criteria

- Aneurysmal SAH of all clinical grades
- First aneurysmal SAH
- Age: 18 years or older
- Pre-morbid modified Rankin Scale score 0 (“no symptoms at all”) or 1 (“no significant disability despite symptoms”)
- Aneurysm treatment performed during the first 48 hours after the initial hemorrhage.
- Informed consent by the patient or his/her legal representative. In case neither the patient being capable of giving informed consent nor a legal representative is available, informed consent can be given by an independent physician neither involved in the patient’s treatment nor in conducting the trial.

Subject Exclusion Criteria

- Subarachnoid hemorrhage of other than aneurysmal origin
- No hemorrhage visible on initial CT scan (Fisher Grade I / modified Fisher Grade 0)
- Pregnancy
- Concurrent participation in another interventional trial (participation in an observational trial is not an exclusion criterion)
- Life expectancy less than 1 year for other reasons than the current SAH
- Other concomitant severe disease that would confound with treatment
- Other clear contraindication for treatment with a lumbar drain (e.g. absent or compressed basal cisterns on the admission CT)

© 2023 Wolf S et al. *JAMA Neurology*.

eTable 2. Reasons Provided for Missed Recruitments

- No informed consent (n=24)
- Late referral (n=42)
- anticoagulation necessary (n=23)
- no drain considered (n=21)
- primarily use of lumbar drain (n=14)
- poor grade (n=63)
- patient missed (n=18)
- no aneurysm detected (n=47)
- other reasons / no reason given (n=120)

Screening logs were provided from five of the 19 centers, including the two largest recruiting sites. In summary, there were 591 patients seen by these centers, resulting in 219 randomizations and 372 patients not being considered.
No information is available for the number of patients screened at the 14 remaining sites, which contributed 88 recruitments.

© 2023 Wolf S et al. *JAMA Neurology*.

eFigure 2. Time Flow of Randomizations

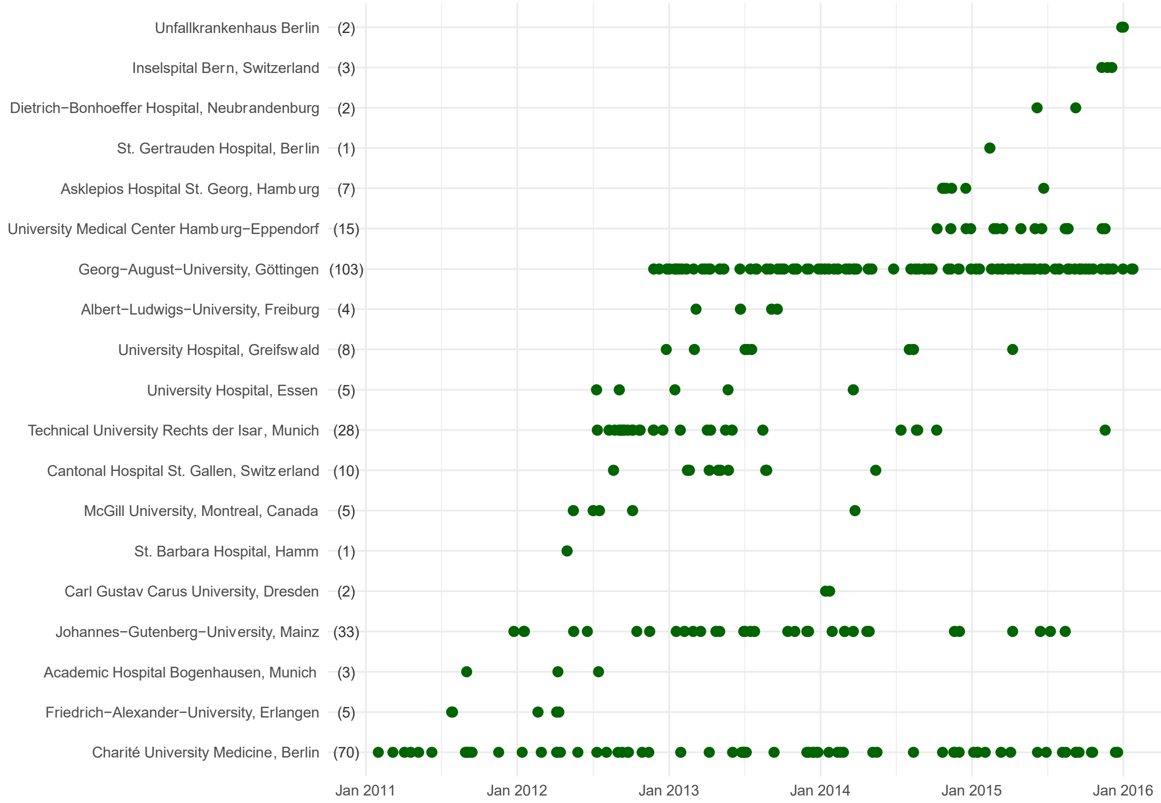

Centers are ordered according to the date of their initiation for the EARLYDRAIN study. Numbers in parenthesis correspond to the number of randomizations contributed.

Invalid Recruitment

307 randomizations were performed for the EARLYDRAIN trial. In 20 instances, patients failed allocation and treatment according to their assignment.

Main reason for an allocation failure was lack or withdrawal of informed consent (four patients in each group). Two patients in the Lumbar Drain group and one in the Standard of Care group unexpectedly required antiplatelet treatment and therapeutic anticoagulation during or after aneurysm coiling. The eventual placement of a lumbar drain after the intervention was not feasible. Two patients in the Lumbar Drain group accidently got randomized twice due to a delay in response from the randomization server. Two patients in the Lumbar Drain group had aneurysm treatment later than 48 hours after the index subarachnoid hemorrhage, thus prohibiting starting lumbar drainage in due time as specified by the protocol. No source data was traceable for one patient in each group. In one patient in the Standard of Care group, no aneurysm was detected as bleeding source, and the patient was excluded from participating in EARLYDRAIN. In one instance, the patient was erroneously randomized and allocated to the Standard of Care group. In all these instances, no data was provided from the local investigators.

One patient randomized to the Lumbar Drain group was unable to receive the assigned intervention. Although initial not planned, double-platelet inhibition was required during aneurysm treatment with coiling. Therefore, placement of a lumbar drain was not feasible afterwards and no attempt was considered. This patient died on day eight after subarachnoid hemorrhage due to vasospastic infarctions. The data was excluded from all following analysis.

One patient randomized to the Standard of Care group was unable to receive scheduled aneurysm treatment due to rapidly developing brain edema. The patient died the day after admission. The data was excluded from all following analysis.

© 2023 Wolf S et al. *JAMA Neurology*.

eFigure 3. Daily Mean Arterial Pressure

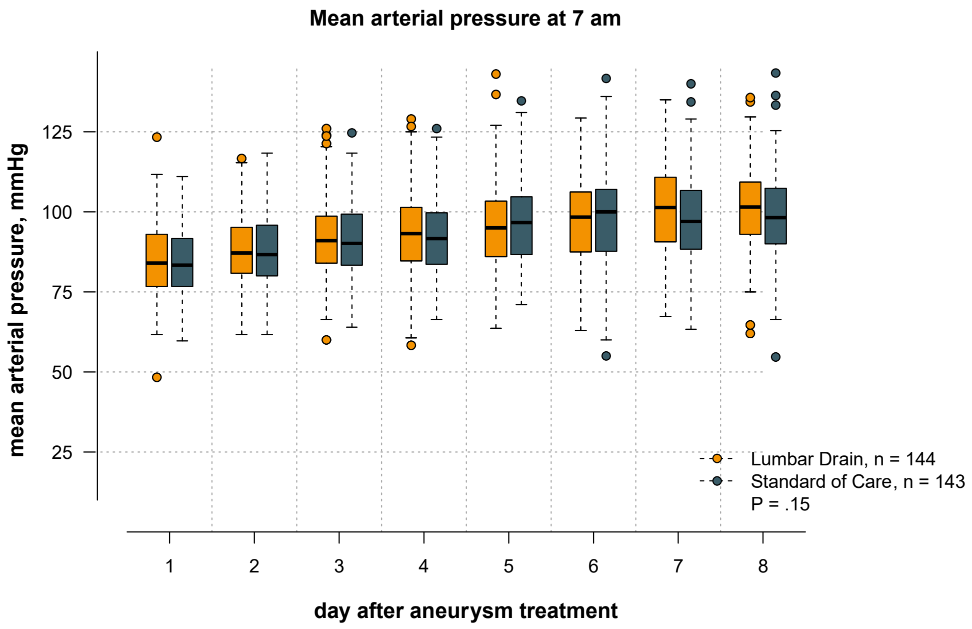

Mean arterial pressure measured at 7 am.

Data is shown as boxplots, with whiskers extending to 1.5 times the interquartile range, representing roughly the 10% and 90% percentiles. P values denote significance of a mixed effect model comparing Lumbar Drain group and Standard of Care group.

© 2023 Wolf S et al. *JAMA Neurology*.

eFigure 4. ICP at 7 AM

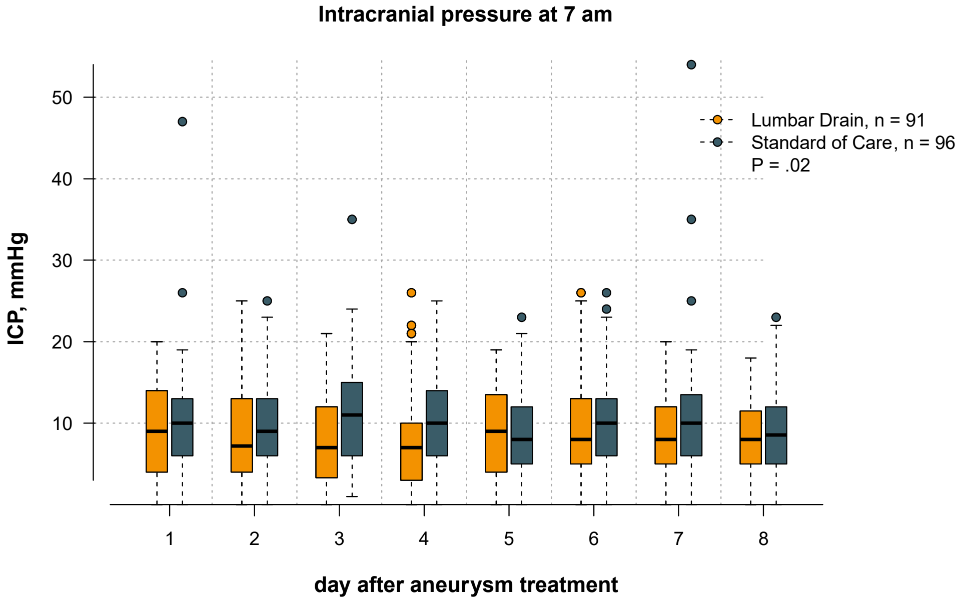

Mean ICP at 7 am was lower by 1.5 mmHg in the Lumbar Drain group. ICP was measured with external ventricular drains or parenchymal probes, according to the local policy of each center. In 100 patients, no ICP recording at 7 am was available or ICP monitoring was not performed.

© 2023 Wolf S et al. *JAMA Neurology*.

eFigure 5. Highest ICP on Each Day

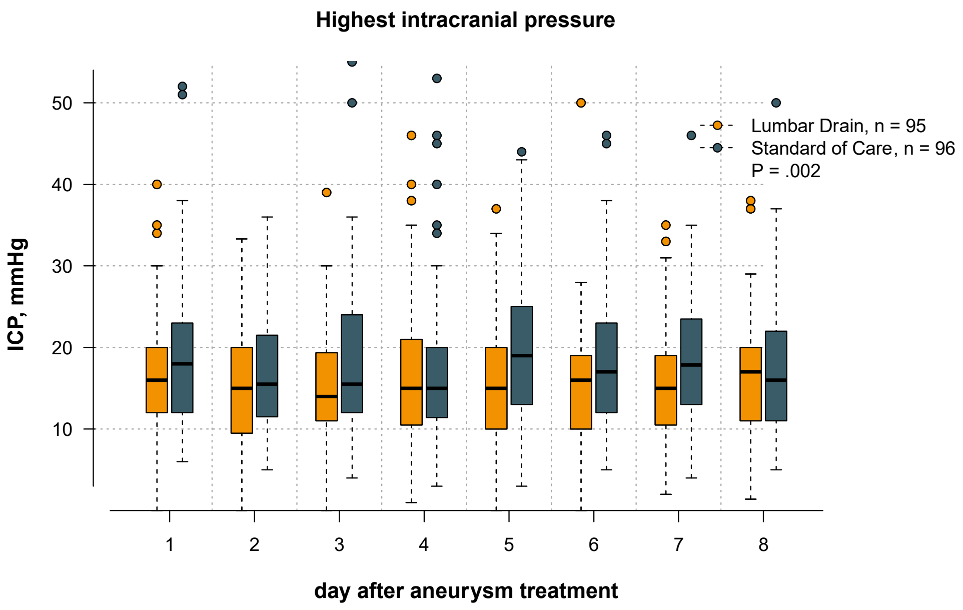

On average, highest ICP was 2.9 mmHg lower in the Lumbar Drain group. No data on highest ICP was available, or no ICP monitoring was performed in 96 patients.

© 2023 Wolf S et al. *JAMA Neurology*.

eFigure 6. TCD Values

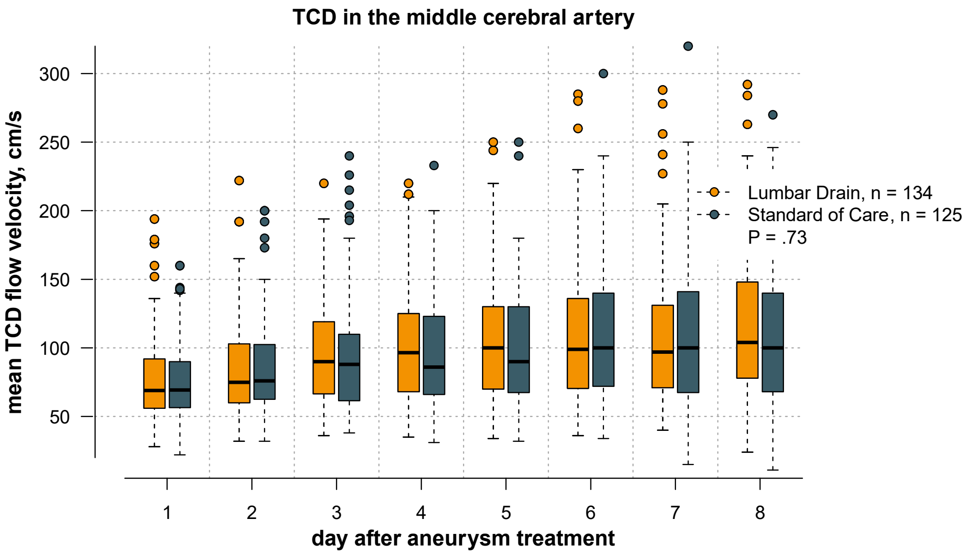

TCD values were recorded with mean flow velocity in the middle cerebral artery at a depth of 50-60 mm. In 28 patients, TCD monitoring was either not performed or no acoustic window present.

© 2023 Wolf S et al. *JAMA Neurology*.

eFigure 7. Fluid Intake Per Day

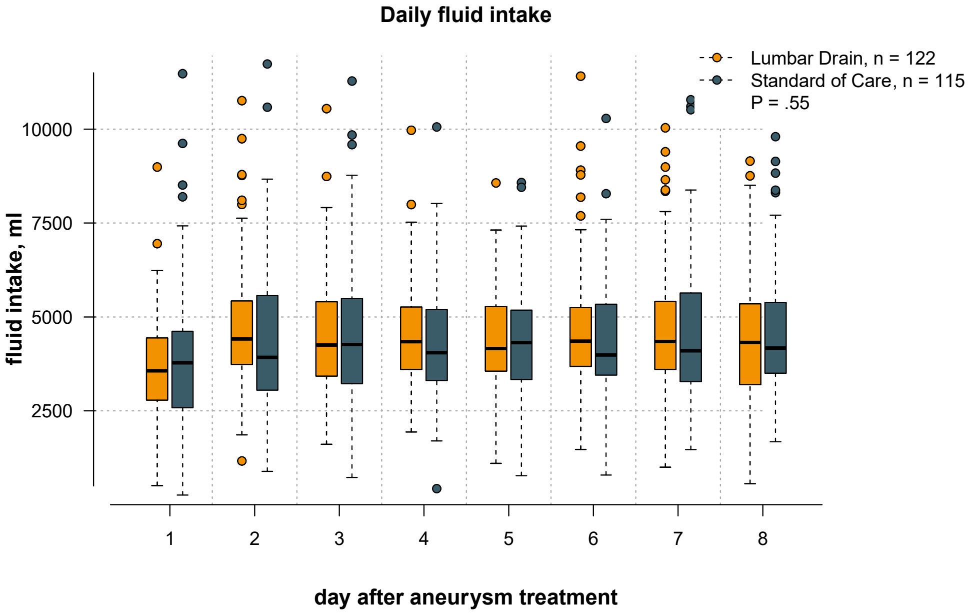

Fluid management was recorded with daily values. In 50 patients, no data on fluid intake and fluid balance was available.

© 2023 Wolf S et al. *JAMA Neurology*.

eFigure 8. Net Fluid Balance Per Day

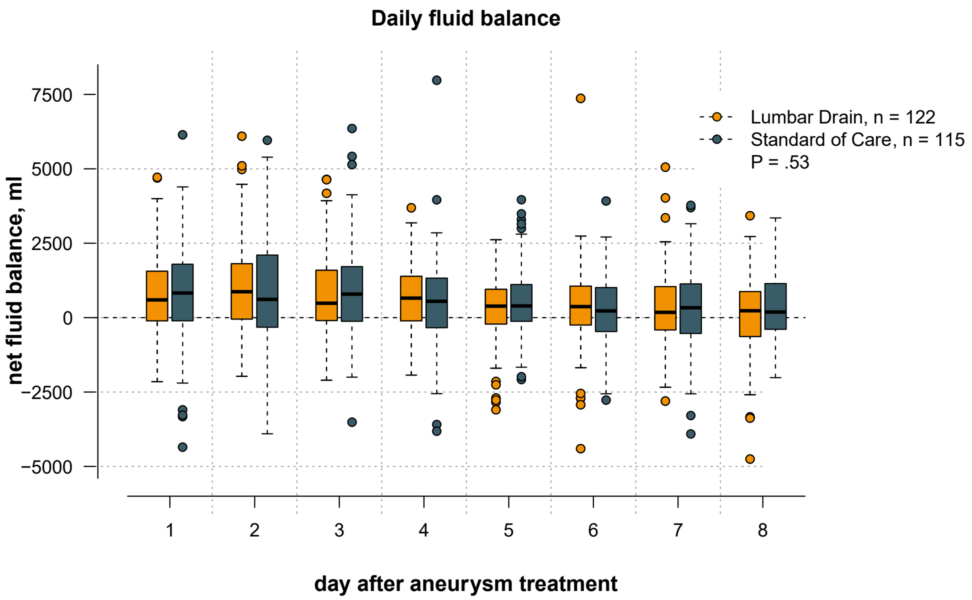

© 2023 Wolf S et al. *JAMA Neurology*.

eFigure 9. Comparison of Fever Burden

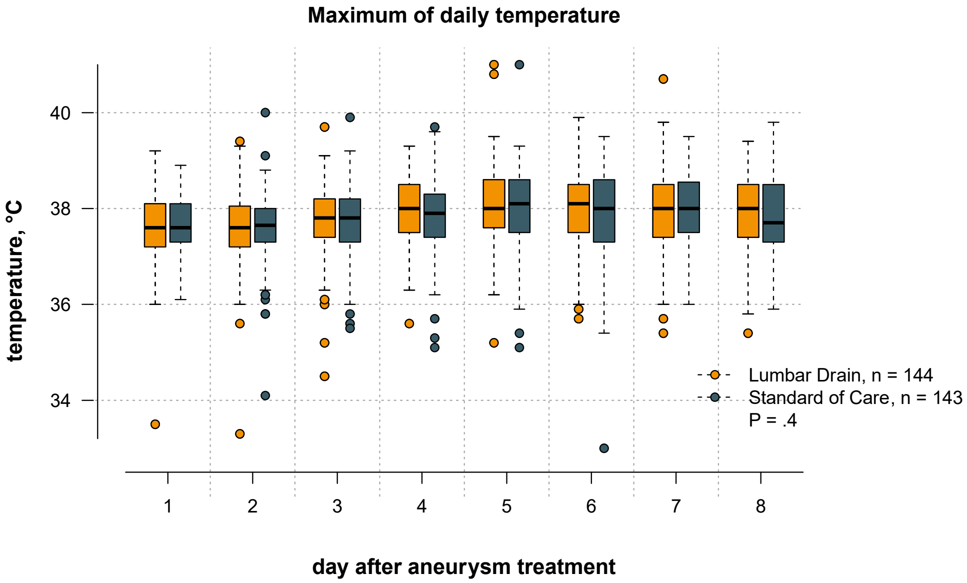

© 2023 Wolf S et al. *JAMA Neurology*.

eFigure 10. Lowest Hemoglobin Value Per Day

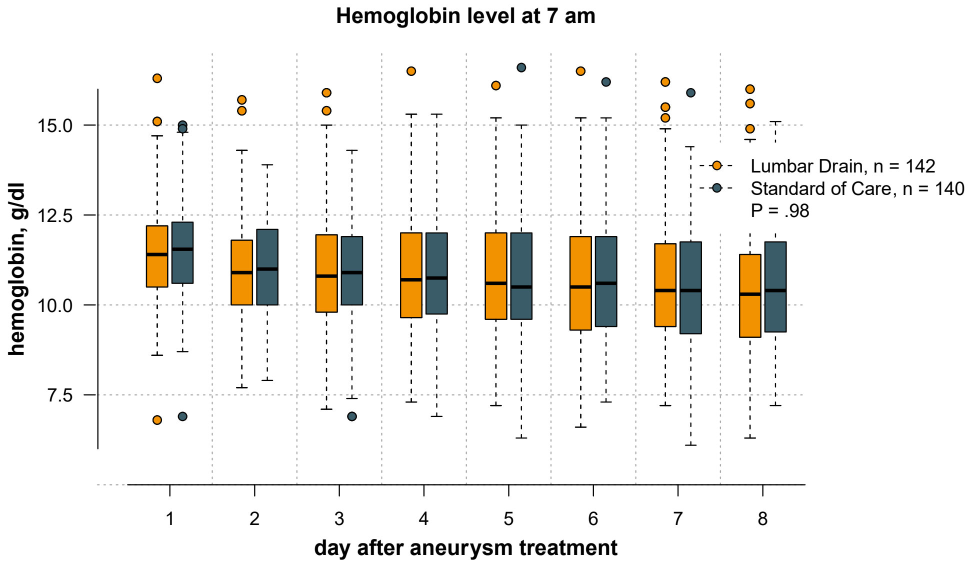

Hemoglobin measurement was performed with centralized lab and local blood gas analyzers. In five patients, no data on hemoglobin was recorded.

© 2023 Wolf S et al. *JAMA Neurology*.

eFigure 11. Drainage via EVD

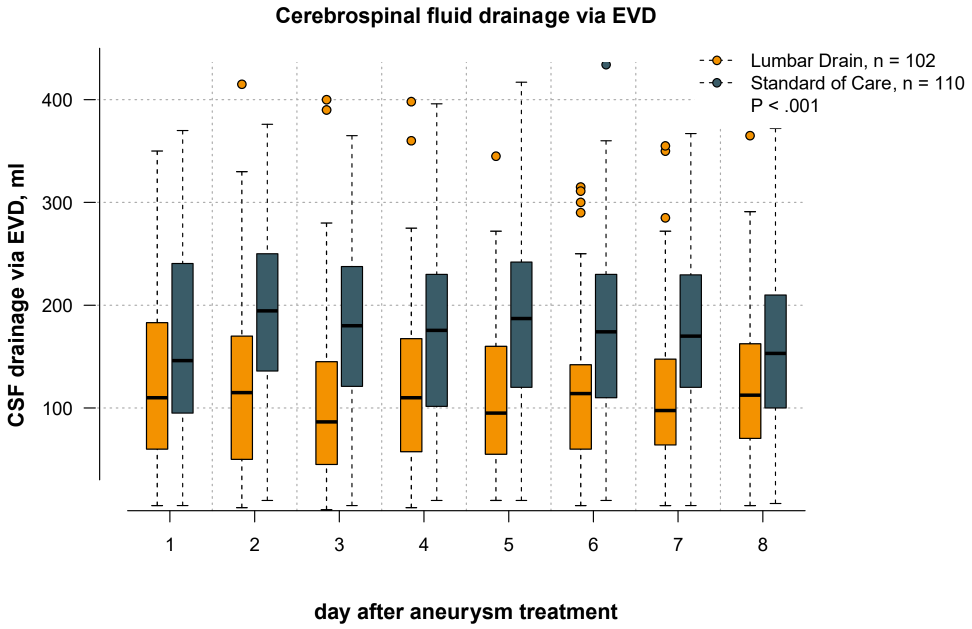

Drainage of cerebrospinal fluid was performed via EVD on clinical demand at the discretion of the local investigators.
75 patients did not require treatment with an external ventricular drain. On average, drainage via EVD was 60 ml less per day in the Lumbar Drain group.

© 2023 Wolf S et al. *JAMA Neurology*.

eFigure 12. Drainage via Lumbar Drain

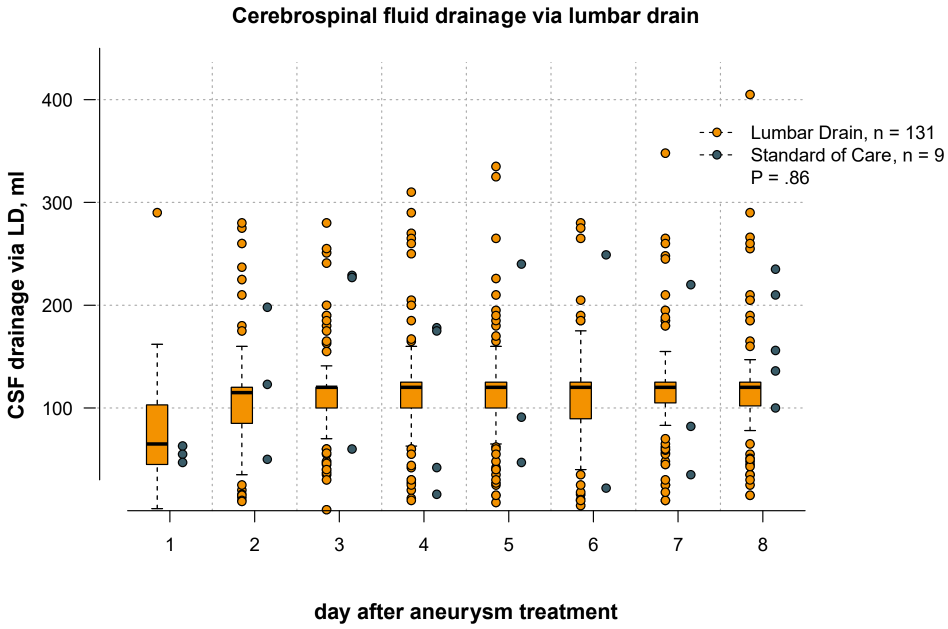

In the Lumbar Drain group, a fixed rate of 5 ml per hour was proposed via lumbar route. 13 patients in the Lumbar Drain group did not receive a lumbar drain at any time. Main reason for not performing lumbar drainage as specified in the protocol were either failure to place the drain or early clotting with consecutive removal.

For the Standard of Care patients, only single data points are shown. Nine patients had a lumbar drain inserted either as allocation error, for treatment of hydrocephalus in patients without an EVD, or in once case as rescue therapy for refractory ICP. Two of these 9 patients showed more than 480 ml of CSF drainage during the first 8 days and were considered protocol violations.

© 2023 Wolf S et al. *JAMA Neurology*.

eFigure 13. Cerebrospinal Fluid Drainage of External Ventricular and Lumbar Drains Combined

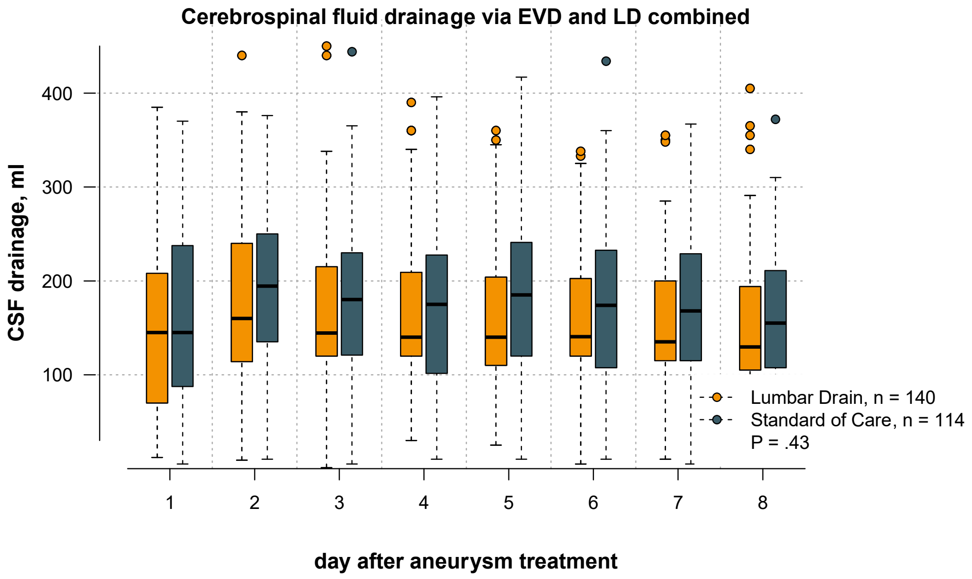

33 patients had neither an external ventricular drainage nor a lumbar drain implanted.

© 2023 Wolf S et al. *JAMA Neurology*.

eTable 3. Clinical Data, Intention-to-Treat Analysis

	Lumbar Drain (n = 144)	Standard of Care (n = 143)	P value
**Aneurysm treatment**			
Aneurysm treatment, day after SAH	1 (0 to 1)	1 (0 to 1)	.26
Recurrent SAH before treatment	9 (6.2)	8 (5.6)	1
Aneurysm treatment			.68
Clipping	68 (47.2)	72 (50.3)	
Coiling	76 (52.8)	71 (49.7)	
Postprocedural CT, day after aneurysm treatment	1 (0 to 1)	1 (0 to 1)	.4
Infarct after aneurysm treatment	15 (10.4)	19 (13.3)	.57
Hemorrhage after aneurysm treatment	12 (8.3)	13 (9.1)	.99
**Vasospasm prophylaxis**			
Vasospasm prophylaxis with nimodipine	142 (98.6)	142 (99.3)	1
Vasospasm prophylaxis with Mg++	81 (56.2)	75 (52.4)	.6
Vasospasm prophylaxis with statins	23 (16)	23 (16.1)	1
**Cerebrospinal fluid drainage management**			
Start of recorded ICU treatment, day after SAH	2 (1 to 2)	2 (1 to 2)	.34
Patients with EVD	102 (70.8)	110 (76.9)	.3
Daily drainage via EVD, ml	98 (60 to 150)	171 (110 to 225)	< .001
Total drainage via EVD, ICU day 1-8, ml	552 (239 to 974)	1218 (814 to 1662)	< .001
Patients with LD	131 (91)	9 (6.3)	< .001
Start of lumbar drainage, day after SAH	2 (1 to 2)	4 (2 to 7)	.02
Daily drainage via LD, ml	108 (92 to 118)	96 (55 to 116)	.34
Total drainage via LD, ICU day 1-8, ml	790 (590 to 898)	191 (55 to 321)	.006
Total CSF drainage, ICU day 1-8, ml [a]	1170 (792 to 1597)	1202 (776 to 1657)	.77
**Vasospasm assessment**			
Angiographic vasospasm assessment, day after SAH [b]	9 (7 to 10)	8 (7 to 10)	.89
Amount of angiographic vasospasm [b]			.59
no vasospasm	61 (54)	61 (56)	
up to 33%	14 (12.4)	19 (17.4)	
up to 66%	24 (21.2)	19 (17.4)	
more than 66%	14 (12.4)	10 (9.2)	
Endovascular rescue treatment	10 (6.9)	14 (9.8)	.51
**Hospital stay**			
Days in acute hospital, day after SAH	25 (19 to 32)	23 (18 to 31)	.22
Last imaging before discharge, day after SAH	18 (11 to 26)	17 (12 to 27)	.99
Final imaging modality			1
Computed tomography	132 (91.7)	131 (91.6)	
Magnetic resonance imaging	12 (8.3)	12 (8.4)	
Discharge location			.34
Home	45 (31.2)	33 (23.1)	
Rehabilitation	74 (51.4)	79 (55.2)	
other hospital	10 (6.9)	9 (6.3)	
died in acute hospital	15 (10.4)	22 (15.4)	
**Interview of surviving patients on day 180**			
Person who was queried [c]			.99
Patient	88 (71)	82 (70.7)	
Relative	27 (21.8)	26 (22.4)	
Healthcare professional	9 (7.3)	8 (6.9)	

Data are median (IQR) or n (%). Percentages might not total 100 because of rounding. [a] Numbers do not add up, as medians are reported, and some patients do not have both drains. Difference in significance to eFigure 13 may be explained by different statistical approaches. [b] No angiography performed after aneurysm occlusion in 65 patients due to early death or local standard operating procedures. [c] Data on 240 surviving patients (3 missing data) were available.

© 2023 Wolf S et al. *JAMA Neurology*.

eTable 4. Univariate Analysis of Clinical Risk Factors for Secondary Infarctions

	Relative Risk	95% CI	P value	AIC
age, per year increase	1.00	0.99 to 1.01	.98	372.5
female sex	1.26	0.88 to 1.73	.21	370.9
Hunt-Hess grade > 2	1.78	1.29 to 2.34	.001	361.6
Hunt-Hess grade > 3	2.22	1.68 to 2.75	< .001	348.3
WFNS grade > 2	1.74	1.27 to 2.26	.001	361.7
WFNS grade > 3	2.02	1.5 to 2.57	< .001	354.5
modified Fisher grade 4	1.29	0.92 to 1.72	.13	370.2
intracerebral hemorrhage	1.51	1.1 to 1.95	.01	366.2
intraventricular hemorrhage	1.26	0.89 to 1.69	.18	370.7
intraventricular or parenchymal hemorrhage	1.32	0.91 to 1.83	.14	370.3
posterior circulation aneurysm	0.77	0.43 to 1.21	.3	371.4

AIC = Akaike Information Criterion

Clinical grading according to Hunt and Hess or WFNS and radiological grading scales showed collinearity. Models with lower Akaike Information Criterion values were chosen for multivariate analysis. Accordingly, in multivariate assessment of clinical risk factors for infarction, adjustment for baseline imbalances was performed with the parameters Hunt-Hess grade larger than 3 and intracerebral or intraventricular hemorrhages (Table 2, main manuscript). Additionally, age was included to maintain comparability with multivariate assessment of outcome measured by the modified Rankin scale.

© 2023 Wolf S et al. *JAMA Neurology*.

eTable 5. Clinical and Radiological Risk Factors for Development of Secondary Infarctions, Stratified Per Factor Level

	Lumbar Drain (n = 144)	Standard of Care (n = 143)	P value
Hunt-Hess classification			< .001
1	5 / 29 (17.2%)	6 / 25 (24%)	
2	8 / 41 (19.5%)	10 / 28 (35.7%)	
3	6 / 25 (24%)	8 / 34 (23.5%)	
4	12 / 20 (60%)	14 / 24 (58.3%)	
5	10 / 29 (34.5%)	19 / 32 (59.4%)	
WFNS classification			< .001
1	11 / 53 (20.8%)	10 / 42 (23.8%)	
2	4 / 22 (18.2%)	9 / 21 (42.9%)	
3	1 / 7 (14.3%)	1 / 10 (10%)	
4	6 / 14 (42.9%)	5 / 15 (33.3%)	
5	19 / 48 (39.6%)	32 / 55 (58.2%)	
Modified Fisher classification			.09
1	0 / 7 (0%)	0 / 3 (0%)	
2	1 / 5 (20%)	3 / 7 (42.9%)	
3	13 / 47 (27.7%)	19 / 54 (35.2%)	
4	27 / 85 (31.8%)	35 / 79 (44.3%)	
Intracerebral hemorrhage			.02
No	21 / 88 (23.9%)	31 / 83 (33.3%)	
Yes	20 / 56 (35.7%)	26 / 50 (52%)	
Intraventricular hemorrhage			.23
No	13 / 54 (24.1%)	20 / 58 (34.5%)	
Yes	28 / 90 (31.1%)	37 / 85 (43.5%)	

Data are n / N (%). Comparison of factor levels with χ^2 test.

© 2023 Wolf S et al. *JAMA Neurology*.

eTable 6. Vasospasm Assessment According to Clinical Estimation and Infarction at Discharge

	**No infarct at discharge (n = 189)**	**Infarct at discharge (n = 98)**
No clinical vasospasm	139 (73.5%)	59 (60.2%)
Clinical vasospasm suspected	50 (26.5%)	39 (39.8%)

Data are n (%). χ^2 test, P = .03.

© 2023 Wolf S et al. *JAMA Neurology*.

eTable 7. Vasospasm Assessment by TCD and Infarctions at Discharge

	**No infarct at discharge (n = 189)**	**Infarct at discharge (n = 98)**
no TCD performed	18 (9.5%)	17 (17.3%)
TCD not suggestive for vasospasm	139 (73.5%)	46 (46.9%)
TCD suggestive for vasospasm	32 (16.9%)	35 (35.7%)

Data are n (%). A threshold of 160 cm/s mean flow velocity in the median cerebral artery at 50-60 mm depth was assumed to be suggestive for vasospasm. Other thresholds provided similar results (data not shown). Percentages might not total 100 because of rounding. χ^2 test, $P < .001$

© 2023 Wolf S et al. *JAMA Neurology*.

eTable 8. Angiographic Vasospasm and Infarctions at Discharge

	**No infarct at discharge (n = 189)**	**Infarct at discharge (n = 98)**
no angiography performed	50 (26.5%)	15 (15.3%)
no vasospasm	78 (41.3%)	44 (44.9%)
up to 33% vasospasm	26 (13.8%)	7 (7.1%)
up to 66% vasospasm	27 (14.3%)	16 (16.3%)
more than 66% vasospasm	8 (4.2%)	16 (16.3%)

Data are n (%). Percentages might not total 100 because of rounding. χ^2 test, P = .001

eTable 9. Infarctions at Discharge and mRS Score at 6 Months

modified Rankin Score at 6 months	No infarct at discharge (n = 189)	Infarct at discharge (n = 98)
0	46 (24.3)	5 (5.1)
1	66 (34.9)	11 (11.2)
2	33 (17.5)	15 (15.3)
3	17 (9)	7 (7.1)
4	11 (5.8)	10 (10.2)
5	10 (5.3)	12 (12.2)
6	6 (3.2)	38 (38.8)

Data are n (%). Percentages might not total 100 because of rounding. χ^2 test, P < .001

© 2023 Wolf S et al. *JAMA Neurology*.

eTable 10. Univariate Analysis of Factors Considered for Outcome Adjustment in Intention-to-Treat Data

	Relative Risk	95% CI	P value	AIC
Age, per year increase	1.04	1.02 to 1.07	< .001	367.3
Female sex	1.08	0.77 to 1.43	.64	386.8
Hunt-Hess grade > 2	2.58	1.94 to 3.23	< .001	355.2
Hunt-Hess grade > 3	2.85	2.31 to 3.32	< .001	335.6
WFNS grade > 2	2.62	2.01 to 3.22	< .001	351
WFNS grade > 3	2.9	2.28 to 3.48	< .001	339.7
Modified Fisher grade 4	1.77	1.33 to 2.24	< .001	374
Intracerebral hemorrhage	2.08	1.63 to 2.51	< .001	361.9
Intraventricular hemorrhage	1.99	1.47 to 2.54	< .001	370
Intracerebral or intraventricular hemorrhage	2.6	1.81 to 3.49	< .001	364.5
Posterior circulation aneurysm	0.86	0.52 to 1.27	.5	386.5

Univariate analysis for outcome predictors in intention-to-treat data

For aneurysmal subarachnoid hemorrhage patients, known risk factors for worse outcome are age, and clinical and radiological severity grades. We analyzed the clinical data available on admission for association with the primary endpoint, unfortunate outcome measured by a modified Rankin Score larger than 2.

© 2023 Wolf S et al. *JAMA Neurology*.

eFigure 14. Mortality in the Intention-to-Treat Data

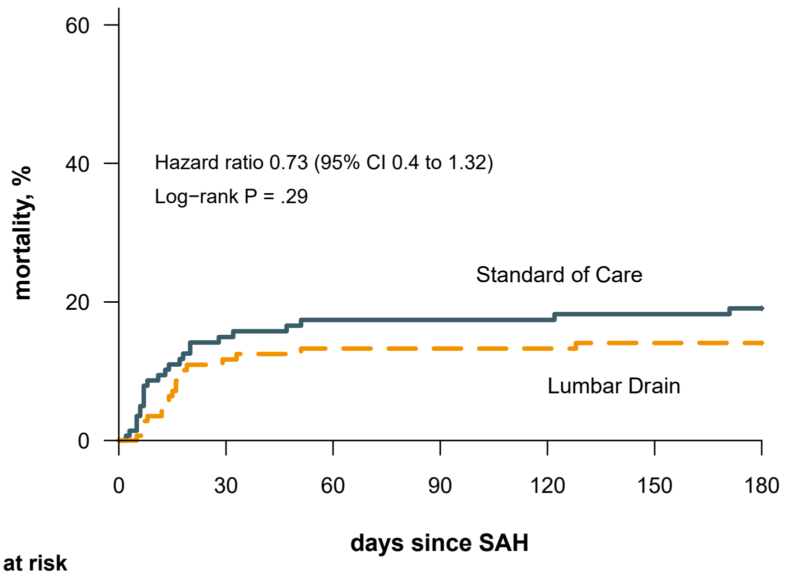

Number at risk							
Lumbar Drain	144	128	126	126	126	125	125
Standard of Care	143	123	120	120	120	119	118

P value and confidence interval from Cox proportional hazard analysis, without adjustment.

© 2023 Wolf S et al. *JAMA Neurology*.

eTable 11. Causes of Death in the Lumbar Drain Group and in the Standard of Care Group

	Lumbar Drain (n = 19)	Standard of Care (n = 25)
**Cause of death**		
cerebral infarction	9 (47.4)	13 (52)
intracranial hemorrhage	0 (0)	2 (8)
brain edema	4 (21.1)	3 (12)
septic shock	4 (21.1)	3 (12)
pneumonia	1 (5.3)	0 (0)
cardiac arrest	0 (0)	1 (4)
unknown / not reported	1 (5.3)	3 (12)

Data are n (%). χ^2 test, P = .51. Percentages might not total 100 because of rounding.

Cerebral herniation was documented for three deceased patients in the Lumbar Drain group and four deceased patients in the Standard of Care group.

© 2023 Wolf S et al. *JAMA Neurology*.

Intention-to-treat subgroup analysis

The following comparisons were performed to visualize the distribution of functional outcomes measured by the modified Rankin Scale in the intention-to-treat population. All p values were derived from logistic regression for a dichotomized mRS score of 0-2 vs. 3-6.

eFigure 15. Outcome in Patients Younger Than and Up to Median Age of 55 Years

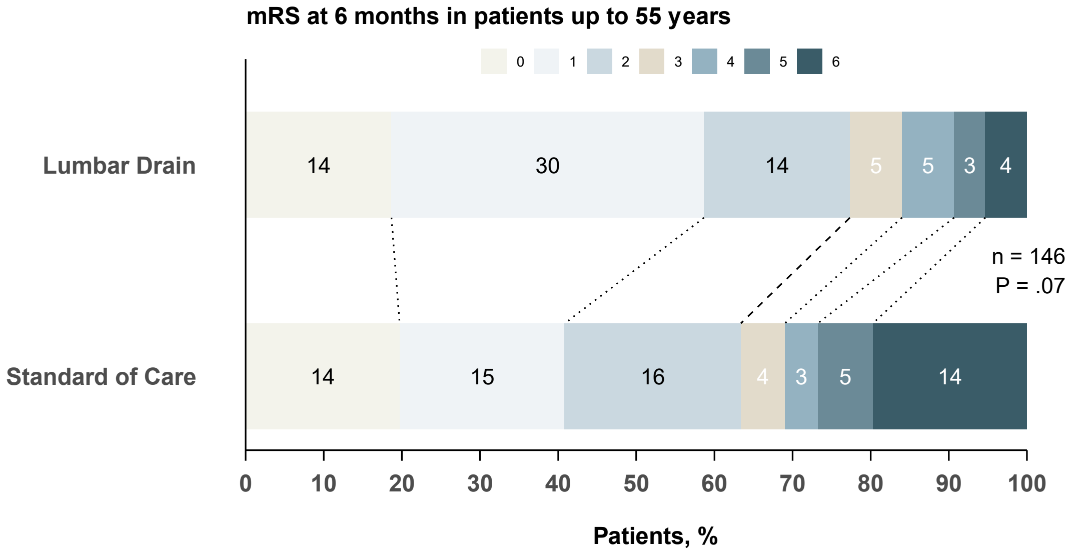

© 2023 Wolf S et al. *JAMA Neurology*.

eFigure 16. Outcome in Patients Older Than Median Age of 55 Years

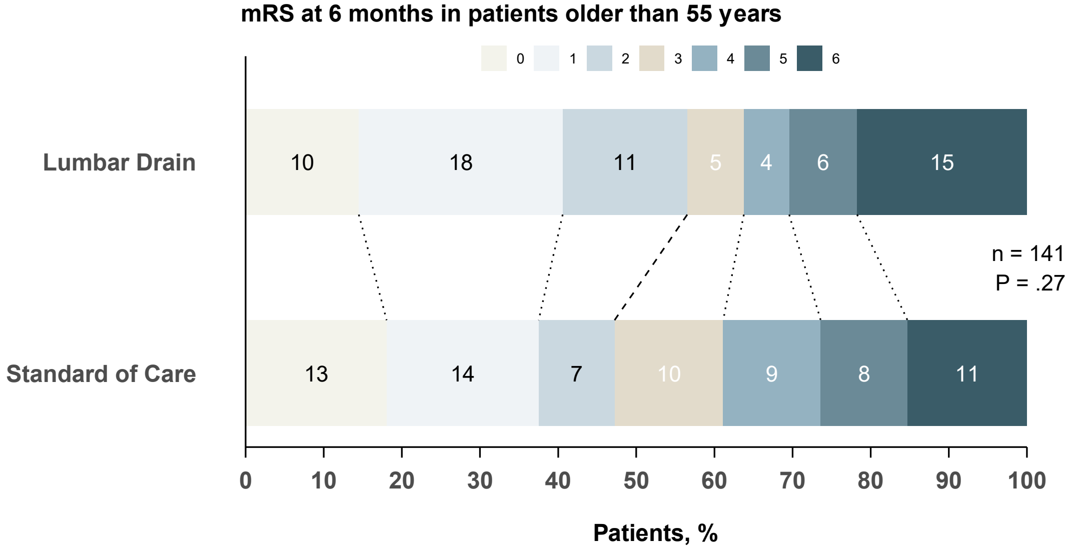

© 2023 Wolf S et al. *JAMA Neurology*.

eFigure 17. Outcome in Female Patients

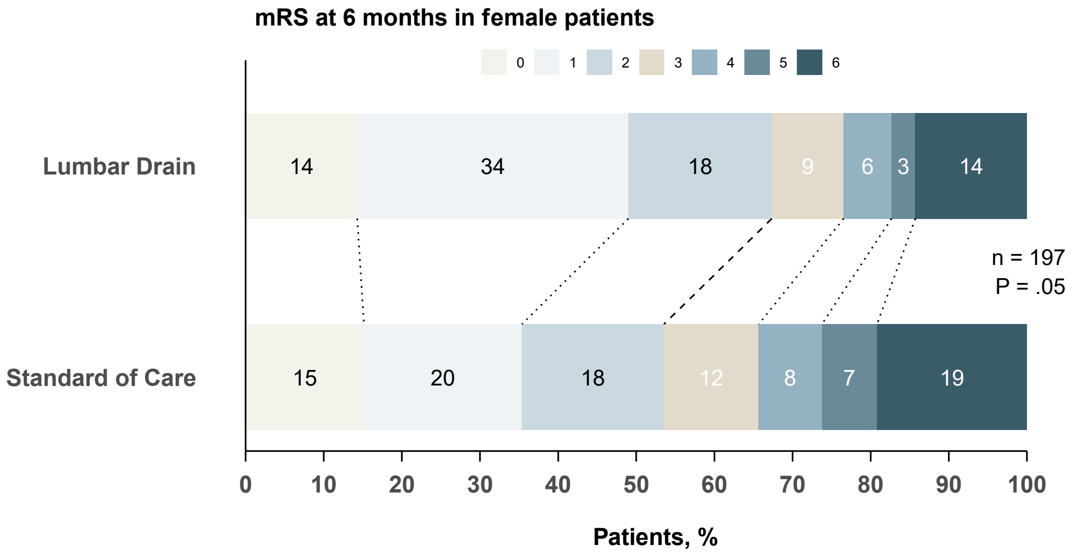

© 2023 Wolf S et al. *JAMA Neurology*.

eFigure 18. Outcome in Male Patients

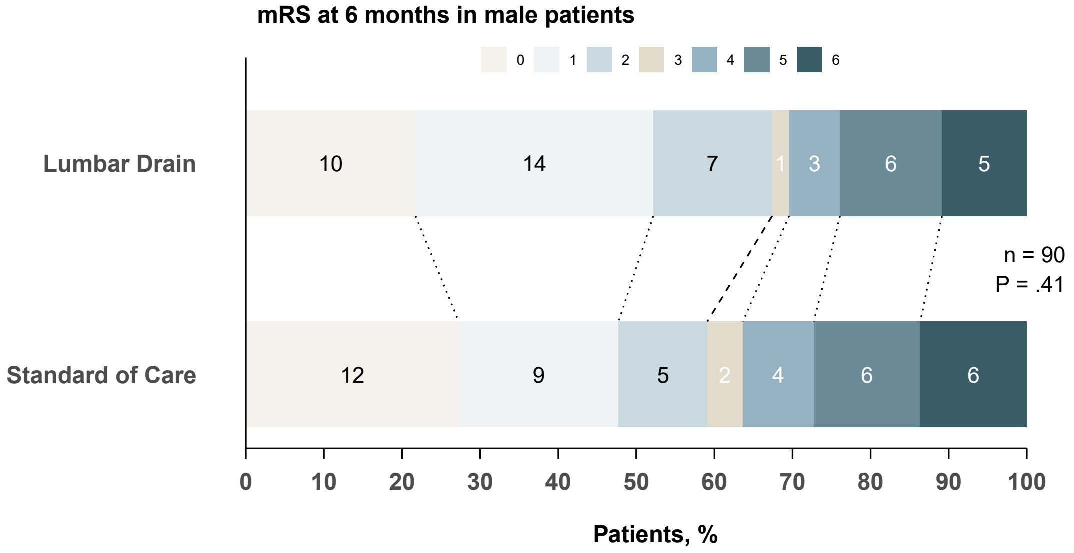

© 2023 Wolf S et al. *JAMA Neurology*.

eFigure 19. Outcome in Good-Grade Patients (Hunt-Hess Grades 1 and 2)

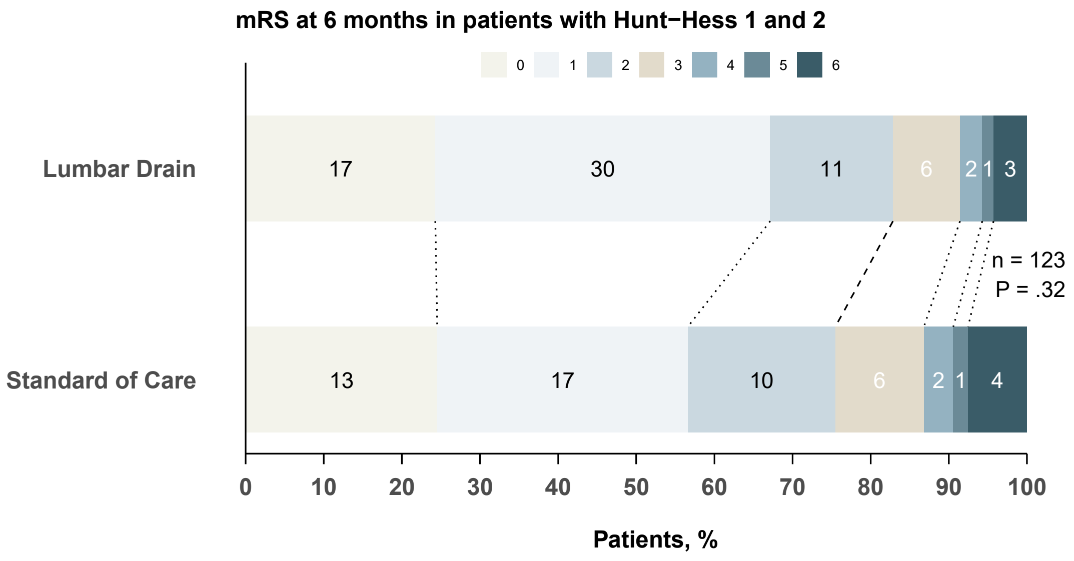

© 2023 Wolf S et al. *JAMA Neurology*.

eFigure 20. Outcome in Poor-Grade Patients (Hunt-Hess Grade 3-5)

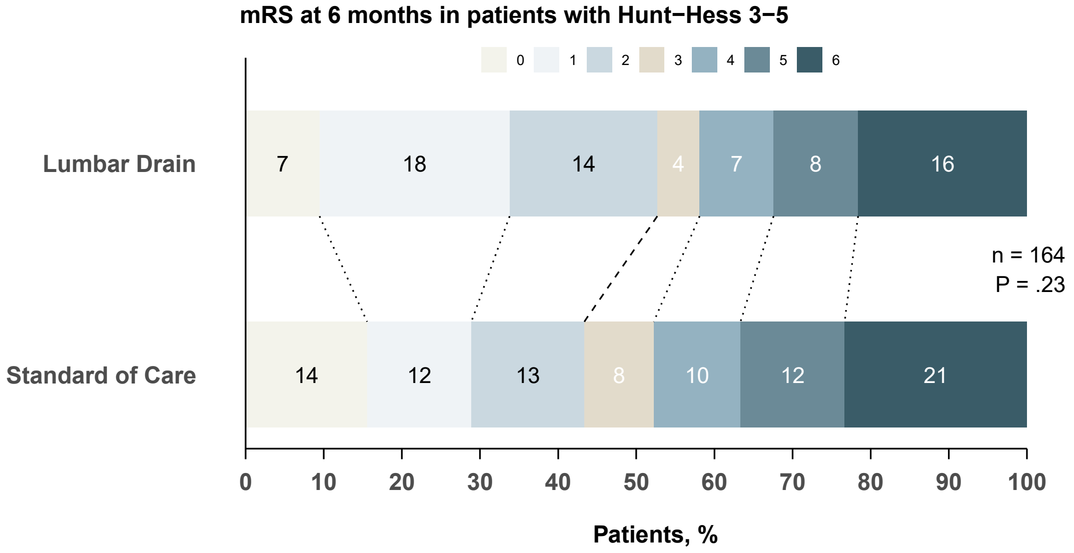

© 2023 Wolf S et al. *JAMA Neurology*.

eFigure 21. Outcome in Good-Grade Patients (WFNS Grades 1-3)

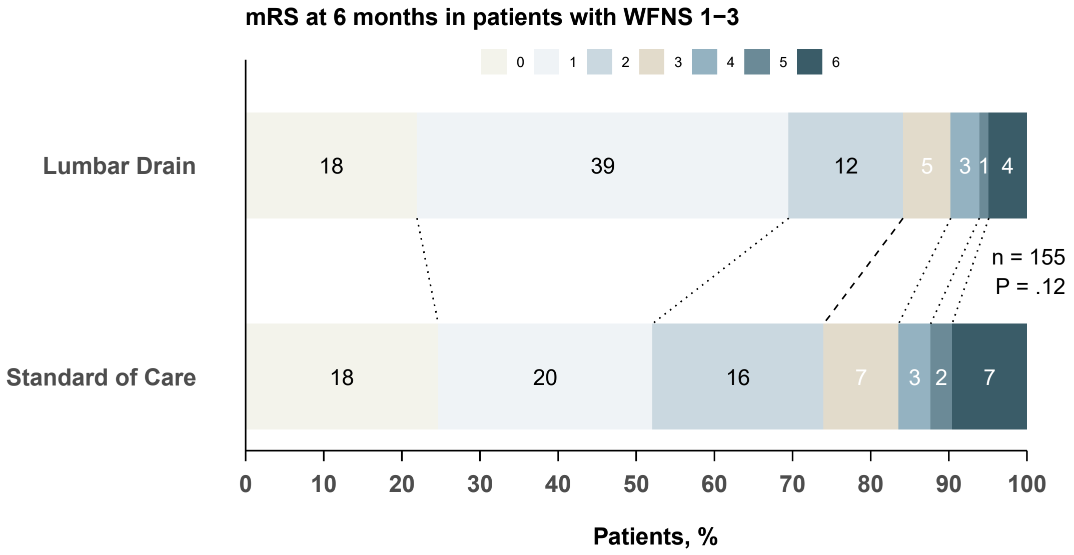

© 2023 Wolf S et al. *JAMA Neurology*.

eFigure 22. Outcome in Poor-Grade Patients (WFNS Grades 4 and 5)

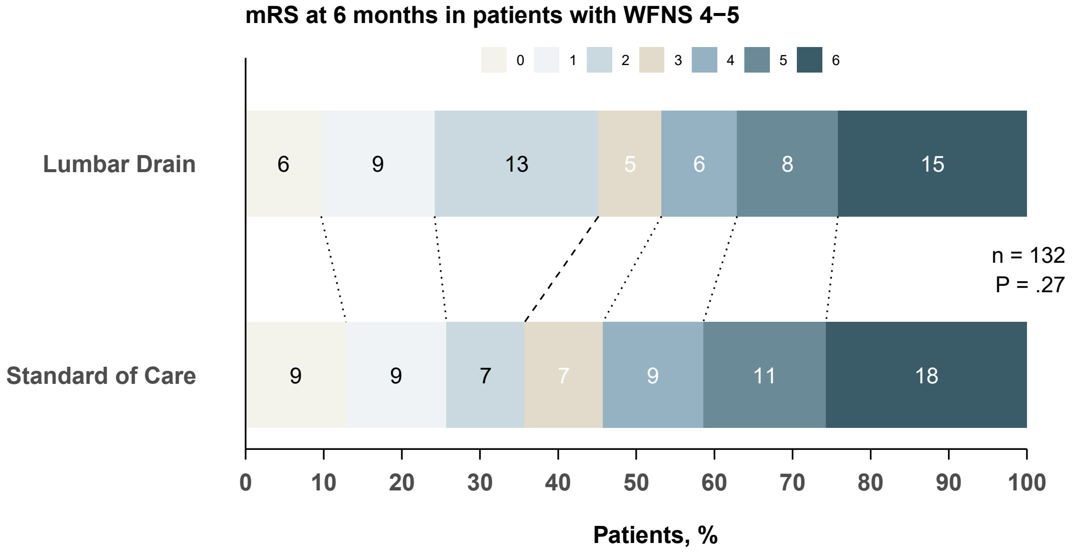

© 2023 Wolf S et al. *JAMA Neurology*.

eFigure 23. Outcome in Patients Presenting Without Intracerebral Hemorrhage

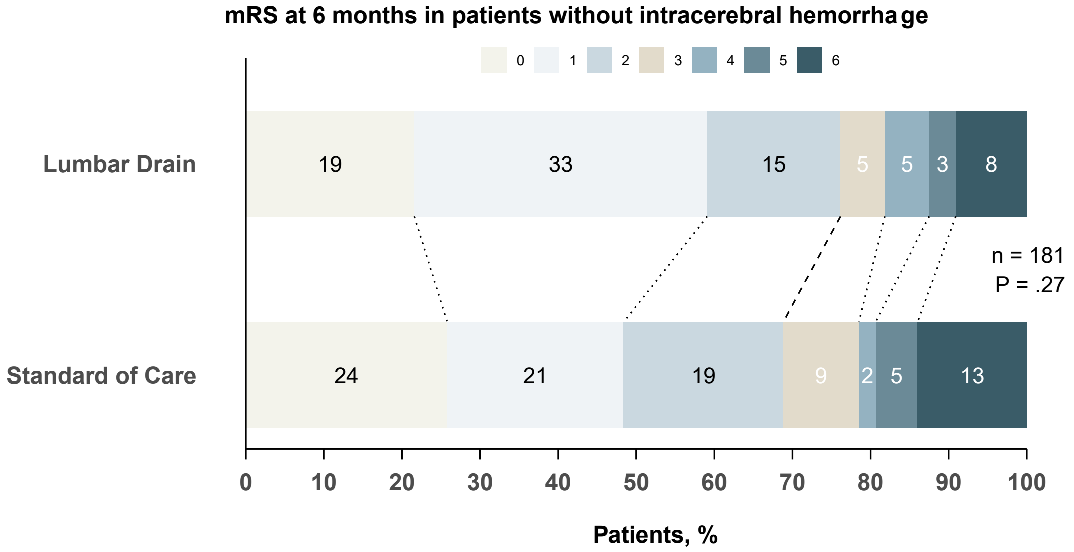

Original Fisher scale grades 2 and 3, all modified Fisher scale grades

© 2023 Wolf S et al. *JAMA Neurology*.

eFigure 24. Outcome in Patients With Intracerebral Hemorrhage

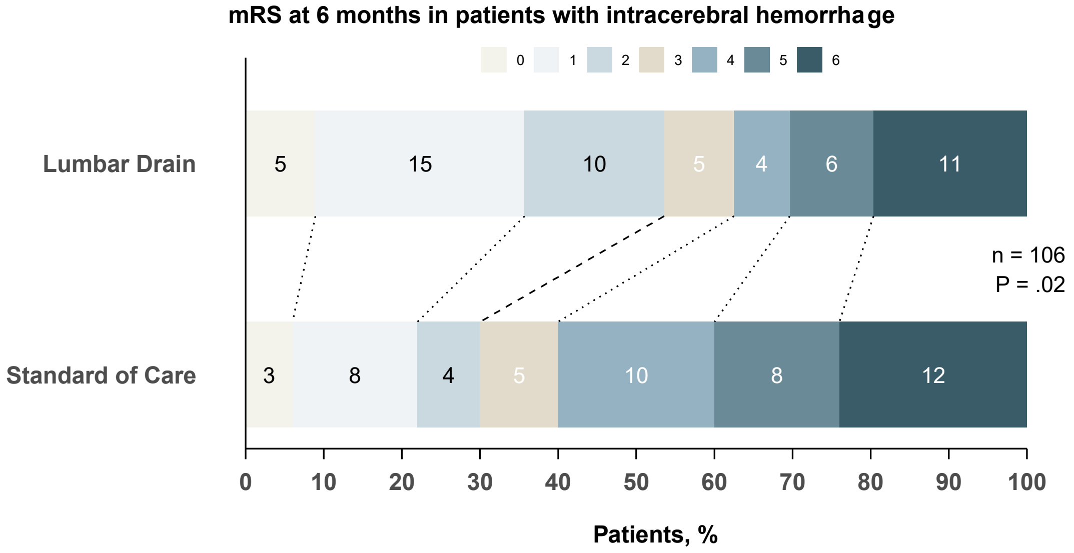

Original Fisher scale grade 4, all modified Fisher scale grades

© 2023 Wolf S et al. *JAMA Neurology*.

eFigure 25. Outcome in Patients Without Intraventricular Hemorrhage

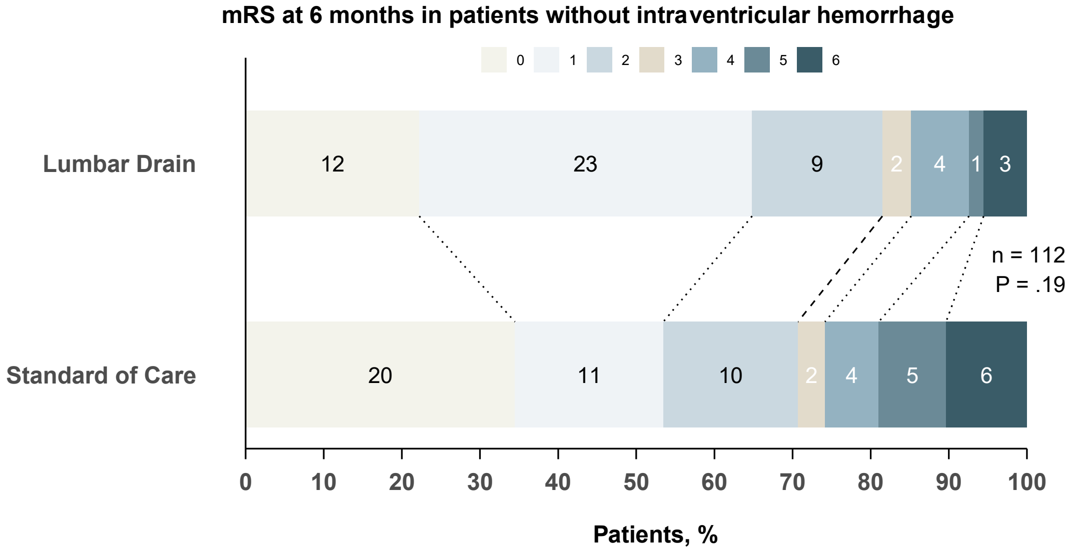

Original Fisher scale grades 2 and 3, modified Fisher grades 1 and 3

© 2023 Wolf S et al. *JAMA Neurology*.

eFigure 26. Outcome in Patients With Intraventricular Hemorrhage

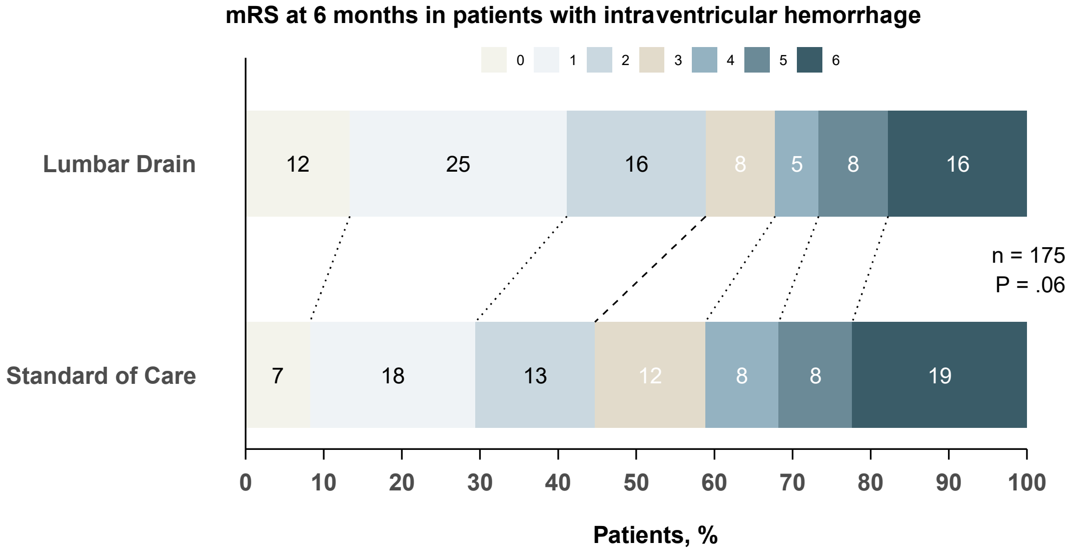

Original Fisher scale grade 4, modified Fisher grades 2 and 4

© 2023 Wolf S et al. *JAMA Neurology*.

eFigure 27. Outcome in Patients With an Aneurysm of the Anterior Circulation

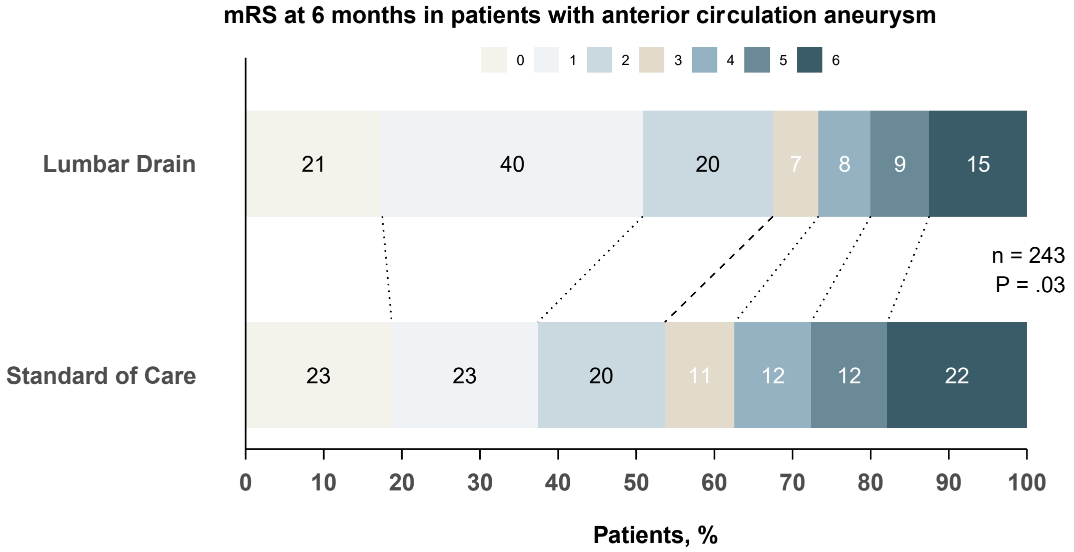

Anterior circulation was defined as all branches of anterior cerebral artery including pericallosal artery and anterior communicating artery, internal carotid artery, middle cerebral artery, and posterior communicating artery

© 2023 Wolf S et al. *JAMA Neurology*.

eFigure 28. Outcome in Patients With Posterior Circulation Aneurysms

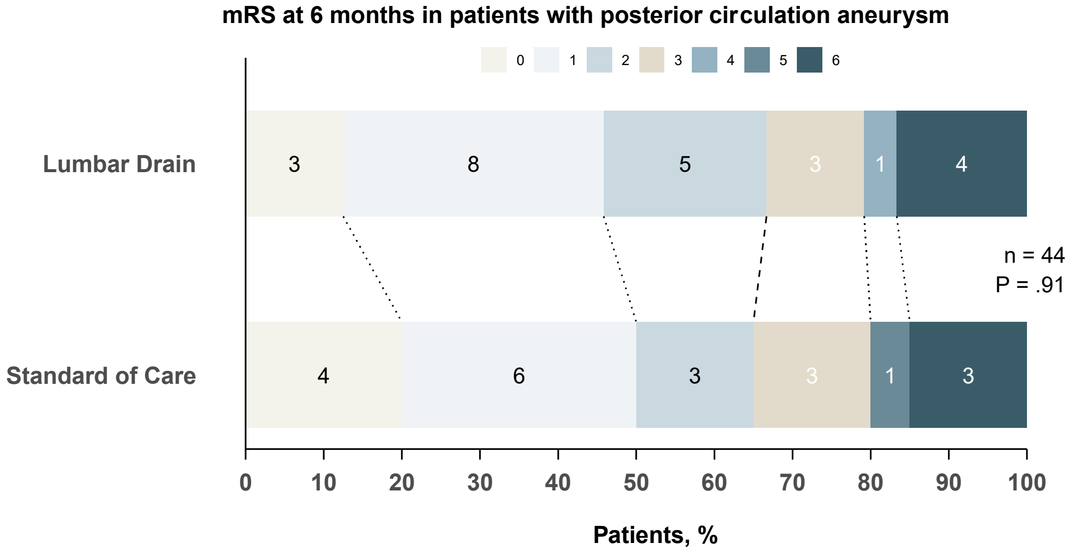

Posterior circulation comprises of vertebral artery, basilar artery, cerebellar vasculature and branches

© 2023 Wolf S et al. *JAMA Neurology*.

eFigure 29. Outcome in Patients Treated With Clipping

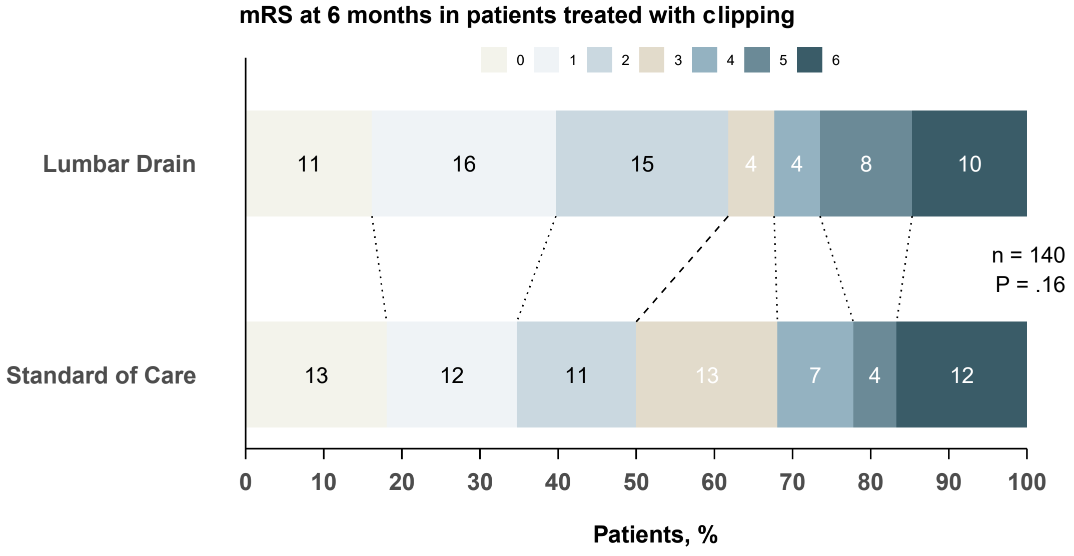

© 2023 Wolf S et al. *JAMA Neurology*.

eFigure 30. Outcome in Patients Treated With Coiling

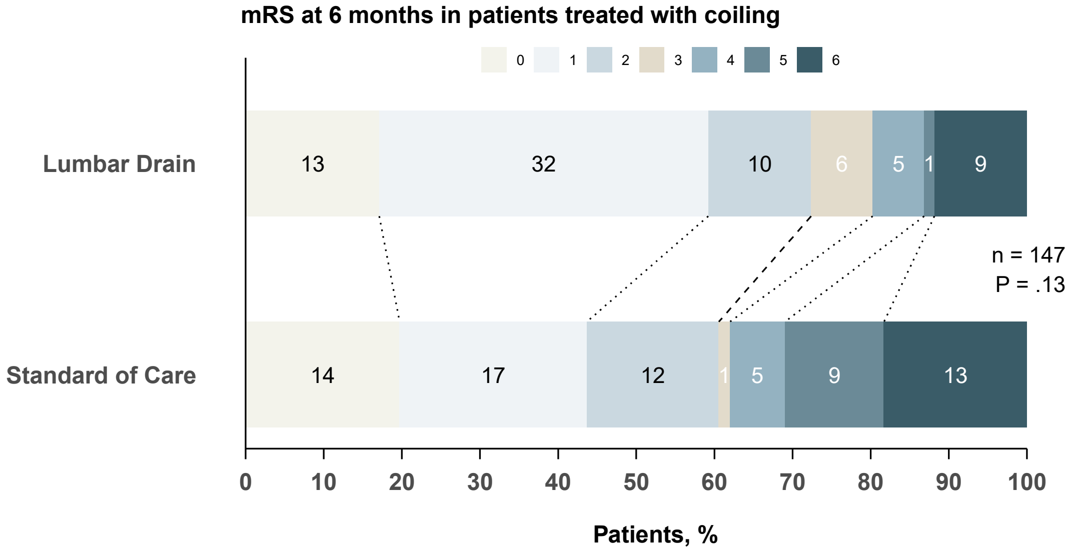

© 2023 Wolf S et al. *JAMA Neurology*.

eFigure 31. Outcome of Patients Treated Without an External Ventricular Drain

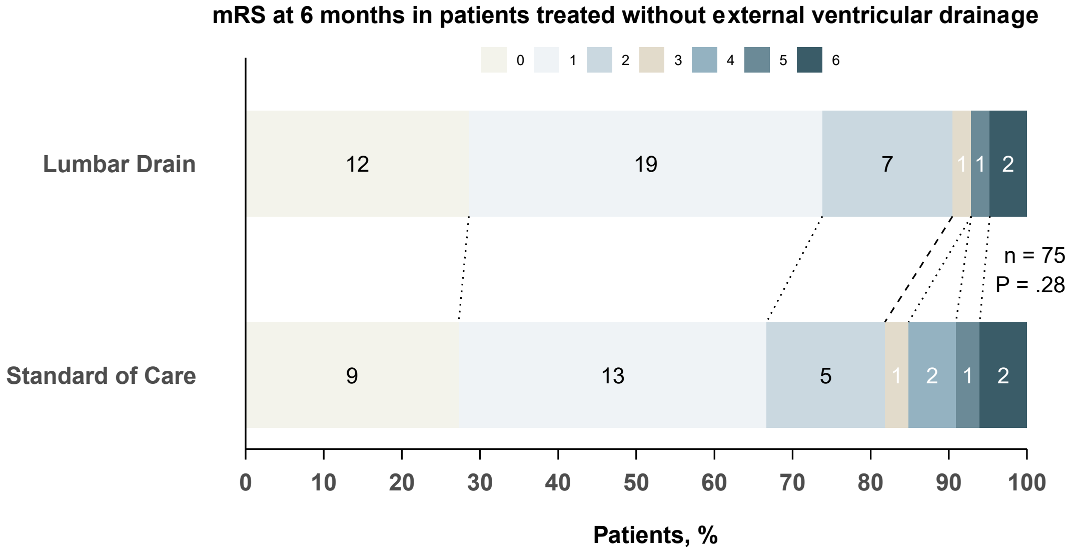

© 2023 Wolf S et al. *JAMA Neurology*.

eFigure 32. Outcome in Patients Where an External Ventricular Drain was Required

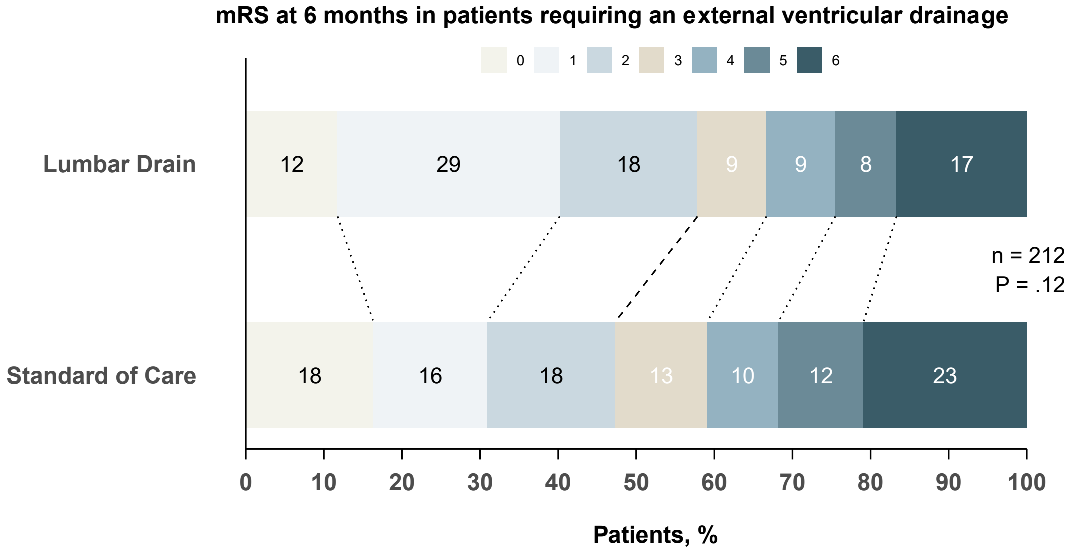

© 2023 Wolf S et al. *JAMA Neurology*.

eFigure 33. Outcome in Patients Treated at the 2 Largest Recruiting Centers

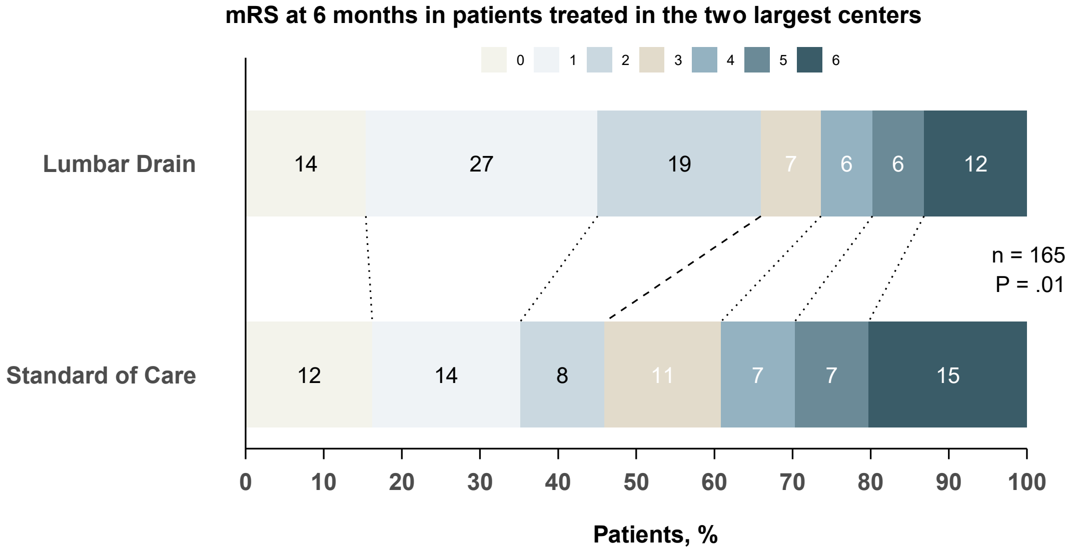

© 2023 Wolf S et al. *JAMA Neurology*.

eFigure 34. Outcome in All Other Than the 2 Largest Recruiting Centers

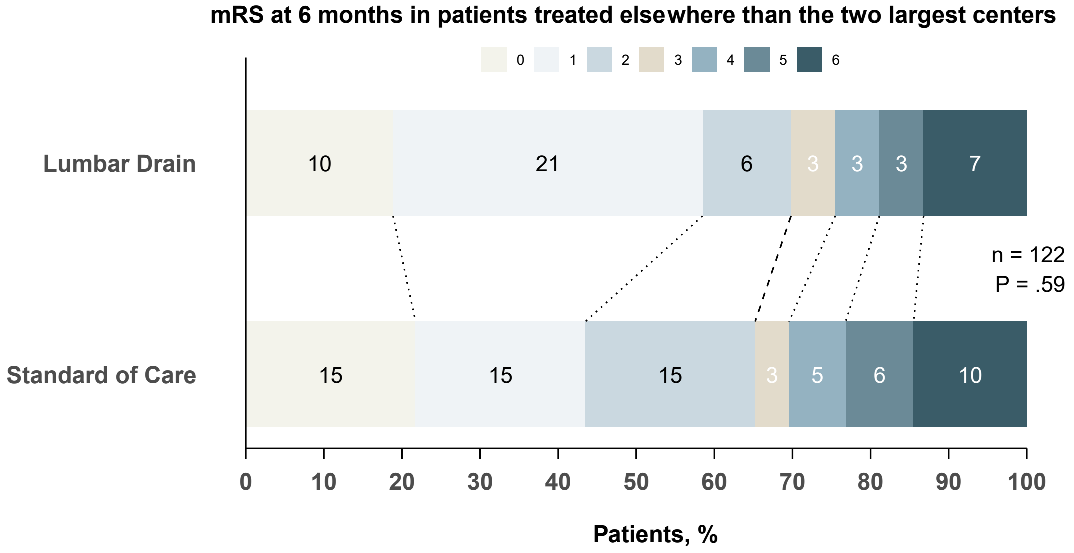

© 2023 Wolf S et al. *JAMA Neurology*.

eAppendix 1. Crossover Patients

Two patients randomized to the Standard of Care group were treated with a lumbar drain directly after aneurysm occlusion. Reasons for cross-over to the Lumbar Drainage group were:

- assignment error (n=1)
- request of neurological consultant (n=1)

These two patients were not considered in the per-protocol analysis. Their data was included in the as-treated analysis in the Lumbar Drain group.

Further seven patients of the Standard of Care group received a lumbar drain for clinical reasons at day 3 or later during the first eight days. Their data was kept in the per-protocol and as-treated analysis as Standard of Care.

35 patients allocated to the use of a lumbar drain after aneurysm occlusion were not treated accordingly. This data was excluded from the per-protocol analysis. It is available in the as-treated analysis in the Standard of Care group.

In 13 of these 35 patients, no attempt to place a lumbar drain was performed. Reasons provided were:

- technically not feasible (n=7)
- assignment error (n=3)
- risk assessment by clinical judgment (n=3)

In 22 patients, a lumbar drain was inserted, but no relevant drainage was performed. Reasons were:

- risk due to ICP difference between EVD and LD (n=1)
- risk assessment by clinical judgment (n=5)
- clotting of drainage (n=8)
- no reason given (n=8)

A comparison of cross-over patients with the regularly treated cohort is given in eTable 10. Only one cross-over patient had a posterior circulation aneurysm. Otherwise, cross-over patients had similar demographic and hemorrhage severity characteristics to patients treated according to protocol. No pattern predisposing to cross-over was noted.

eTable 12. Baseline Characteristics of Crossover Patients Compared With Patients Treated Per Protocol

	Cross-over (n = 37)	Per protocol (n = 250)	P value
Age, years	57 (48 to 64)	54 (48 to 63)	.57
Sex			.06
Female	20 (54.1)	177 (70.8)	
Male	17 (45.9)	73 (29.2)	
Modified Rankin score on admission			.9
0	34 (91.9)	235 (94)	
1	3 (8.1)	15 (6)	
Hunt-Hess classification			.9
1	7 (18.9)	47 (18.8)	
2	10 (27)	59 (23.6)	
3	7 (18.9)	52 (20.8)	
4	7 (18.9)	37 (14.8)	
5	6 (16.2)	55 (22)	
WFNS classification			.49
1	12 (32.4)	83 (33.2)	
2	9 (24.3)	34 (13.6)	
3	2 (5.4)	15 (6)	
4	4 (10.8)	25 (10)	
5	10 (27)	93 (37.2)	
Modified Fisher classification			.47
1	2 (5.4)	8 (3.2)	
2	0 (0)	12 (4.8)	
3	12 (32.4)	89 (35.6)	
4	23 (62.2)	141 (56.4)	
Intracerebral hemorrhage	16 (43.2)	90 (36)	.5
Intraventricular hemorrhage	23 (62.2)	152 (60.8)	1
Aneurysm localization			.19
ACA	4 (10.8)	21 (8.4)	
ACoA	16 (43.2)	75 (30)	
ICA	2 (5.4)	22 (8.8)	
MCA	6 (16.2)	54 (21.6)	
PCoA	8 (21.6)	35 (14)	
BA	1 (2.7)	19 (7.6)	
VA / cerebellar	0 (0)	24 (9.6)	
Number of aneurysms	1 (1 to 1)	1 (1 to 2)	.47
Size of aneurysm, mm [a]	6 (4 to 9)	6 (4 to 8)	.34
Aneurysm circulation			.04
anterior	36 (97.3)	207 (82.8)	
posterior	1 (2.7)	43 (17.2)	

Data are median (IQR) or n (%). Percentages might not total 100 because of rounding. [a] Aneurysm size was not available in eight patients.

© 2023 Wolf S et al. *JAMA Neurology*.

eTable 13. Clinical Treatment Data in Crossover Patients

	Cross-over (n = 37)	Per protocol (n = 250)	P value
Aneurysm treatment, day after SAH	1 (0 to 1)	1 (0 to 1)	.24
Recurrent SAH before treatment	6 (16.2)	11 (4.4)	.01
Aneurysm treatment			1
Clipping	18 (48.6)	122 (48.8)	
Coiling	19 (51.4)	128 (51.2)	
Postprocedural CT, day after aneurysm treatment	1 (0 to 1)	1 (1 to 1)	.31
Infarct after aneurysm treatment	5 (13.5)	19 (11.6)	.95
Hemorrhage after aneurysm treatment	3 (8.1)	22 (8.8)	1
**Vasospasm prophylaxis**			
Vasospasm prophylaxis with nimodipine	36 (97.3)	248 (99.2)	.84
Vasospasm prophylaxis with Mg++	19 (51.4)	137 (54.8)	.83
Vasospasm prophylaxis with statins	7 (18.9)	39 (15.6)	.78
**Cerebrospinal fluid drainage management**			
Start of recorded ICU treatment, day after SAH	2 (1 to 2)	2 (1 to 2)	.74
Patients with EVD	28 (75.7)	184 (73.6)	.95
Daily drainage via EVD, ml	119 (82 to 183)	134 (81 to 193)	.75
Total Drainage via EVD, ICU day 1-8, ml	927 (424 to 1398)	852 (401 to 1418)	.98
Patients with LD	24 (64.9)	116 (46.4)	.05
Start of lumbar drainage, day after SAH	2 (1 to 2)	2 (1 to 2)	.64
Daily drainage via LD, ml	61 (39 to 79)	110 (99 to 122)	< .001
Total Drainage via LD, ICU day 1-8, ml	332 (146 to 404)	812 (673 to 920)	< .001
Total CSF drainage, ICU day 1-8, ml [a]	1164 (547 to 1481)	1181 (814 to 1647)	.12
**Vasospasm assessment**			
Patients clinically suspect for vasospasm	14 (37.8)	75 (30)	.44
Patients with TCD vasospasm [b]	11 (34.4)	56 (25.5)	.39
Angiographic vasospasm assessment, day after SAH [c]	9 (7 to 10)	8 (7 to 10)	.38
Amount of angiographic vasospasm [c]			.78
no vasospasm	15 (46.9)	107 (56.3)	
up to 33%	5 (15.6)	28 (14.7)	
up to 66%	8 (25)	35 (18.4)	
more than 66%	4 (12.5)	20 (10.5)	
Endovascular rescue treatment	5 (13.5)	19 (7.6)	.37
**Hospital stay**			
Days in acute hospital, day after SAH	26 (20 to 37)	24 (19 to 30)	.07
Last imaging before discharge, day after SAH	24 (14 to 30)	17 (11 to 25)	.01
Final imaging modality			.71
Computed tomography	35 (94.6)	1´228 (91.2)	
Magnetic resonance imaging	2 (5.4)	22 (8.8)	
Discharge location			.65
Home	9 (24.3)	69 (27.6)	
Rehabilitation	18 (48.6)	135 (54)	
other hospital	3 (8.1)	16 (6.4)	
died in acute hospital	7 (18.9)	30 (12)	
**Infections and VP shunts**			
Suspected infection of any cause	20 (54.1)	88 (35.2)	.04
VP shunt during acute care	12 (32.4)	56 (22.4)	.26
VP shunt in the first 6 months	13 (35.1)	70 (28)	.48
**Interview of surviving patients on day 180**			
Person who was queried [d]			.71
Patient	19 (65.5)	151 (71.6)	
Relative	7 (24.1)	46 (21.8)	
Healthcare professional	3 (10.3)	14 (6.6)	

Data are median (IQR) or n (%). Percentages might not total 100 because of rounding. [a] Numbers do not add up, as medians are reported, and some patients do not have both drains. [b] No TCD performed in 28 patients. [c] No angiography performed after aneurysm occlusion in 65 patients due to early death or local standard operating procedure. [d] Data on 240 surviving patients (3 missing data) were available. TCD = transcranial doppler.

© 2023 Wolf S et al. *JAMA Neurology*.

eAppendix 2. Per-Protocol Analysis

In the Lumbar Drain group, 109 patients (75.7%) received a lumbar drain after aneurysm treatment and sufficient lumbar drainage as per protocol for the next eight days. In 22 of the Lumbar Drain group patients (15.2%), lumbar cerebrospinal fluid was drained less than specified; in 13 patients (9%) no lumbar drain was placed. Patients with less than specified or no drainage were excluded from the Lumbar Drain group in per-protocol analysis.

134 patients (93.7%) in the Standard of Care group were treated without lumbar drain in the first eight days. Seven patients (4.9%) got a lumbar drain during the first week of treatment but received less than 480 ml of drainage. These patients were kept in the Standard of Care group for per-protocol analysis. Two Standard of Care patients (1.4%) underwent placement of a lumbar drain after aneurysm treatment with consecutive drainage of more than 480 ml and were therefore excluded from per protocol analysis.

In summary, 250 of 287 patients (87.1%) were treated according to protocol, 109 (75.7%) in the Lumbar Drain group and 141 (98.6%) in Standard of Care.

© 2023 Wolf S et al. *JAMA Neurology*.

eFigure 35. CONSORT Diagram for Per-Protocol Analysis

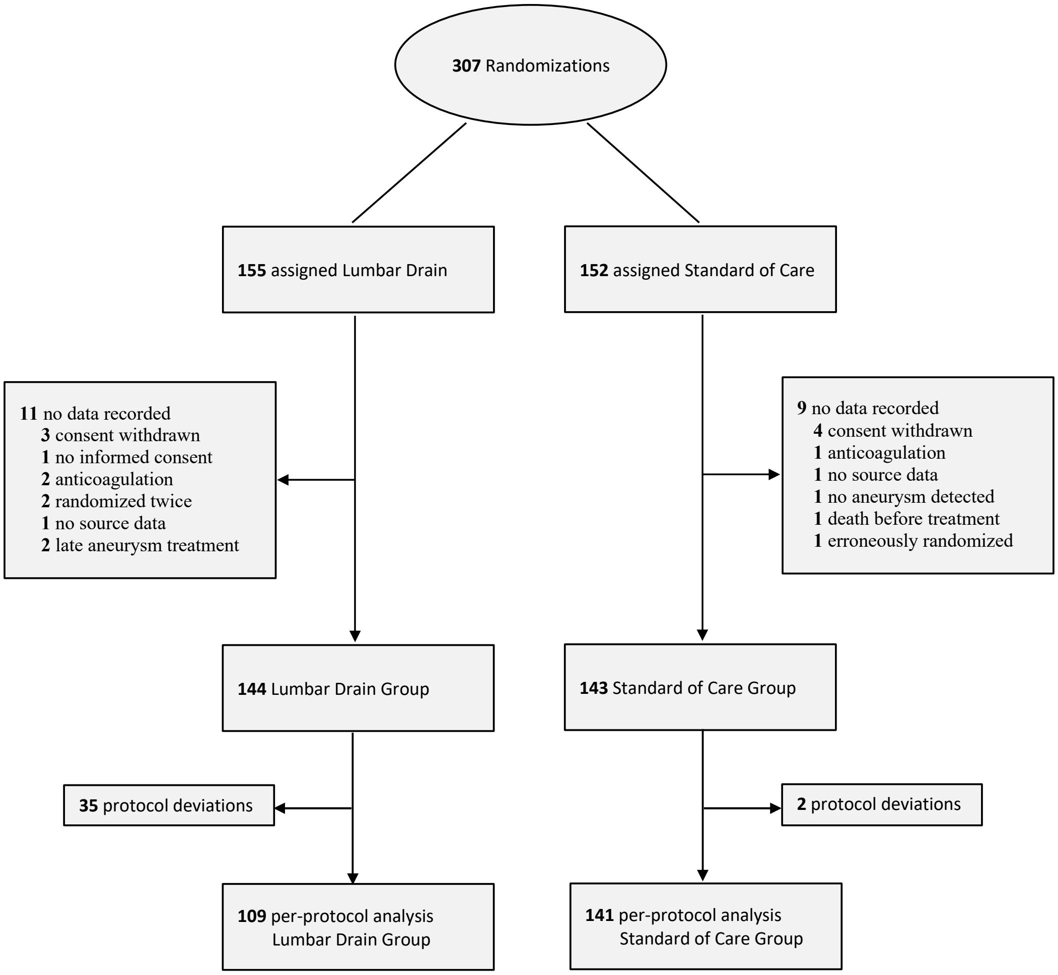

© 2023 Wolf S et al. *JAMA Neurology*.

eTable 14. Demographic Comparison Between Groups in Patients Treated Per Protocol

	Lumbar Drain (n = 109)	Standard of Care (n = 141)	P value
Age, years	54 (49 to 61)	56 (47 to 65)	.52
Sex			.71
Female	79 (72.5)	98 (69.5)	
Male	30 (27.5)	43 (30.5)	
Modified Rankin score on admission			.98
0	103 (94.5)	132 (93.6)	
1	6 (5.5)	9 (6.4)	
Hunt-Hess classification			.19
1	23 (21.1)	24 (17)	
2	32 (29.4)	27 (19.1)	
3	18 (16.5)	34 (24.1)	
4	13 (11.9)	24 (17)	
5	23 (21.1)	32 (22.7)	
WFNS classification			.44
1	43 (39.4)	40 (28.4)	
2	13 (11.9)	21 (14.9)	
3	5 (4.6)	10 (7.1)	
4	10 (9.2)	15 (10.6)	
5	38 (34.9)	55 (39)	
Modified Fisher classification			.66
1	5 (4.6)	3 (2.1)	
2	5 (4.6)	7 (5)	
3	36 (33)	53 (37.6)	
4	63 (57.8)	78 (55.3)	
Intracerebral hemorrhage	40 (36.7)	50 (35.5)	.94
Intraventricular hemorrhage	68 (62.4)	84 (59.6)	.75
Aneurysm localisation			.50
ACA	10 (9.2)	11 (7.8)	
ACoA	34 (31.2)	41 (29.1)	
ICA	7 (6.4)	15 (10.6)	
MCA	19 (17.4)	35 (24.8)	
PCoA	16 (14.7)	19 (13.5)	
BA	9 (8.3)	10 (7.1)	
VA / cerebellar	14 (12.8)	10 (7.1)	
Number of aneurysms	1 (1 to 2)	1 (1 to 1)	.07
Size of aneurysm, mm [a]	5 (4 to 8)	6 (5 to 8)	.06
Aneurysm circulation			.2
Anterior	86 (78.9)	121 (85.8)	
Posterior	23 (21.1)	20 (14.2)	

Data are median (IQR) or n (%). Percentages might not total 100 because of rounding. [a] Aneurysm size was not available in eight patients.

© 2023 Wolf S et al. *JAMA Neurology*.

eTable 15. Clinical Treatment Data in Patients Treated Per Protocol

	Lumbar Drain (n = 109)	Standard of Care (n = 141)	P value
**Aneurysm treatment**			
Aneurysm treatment, day after SAH	1 (0 to 1)	1 (0 to 1)	.24
Recurrent SAH before treatment	3 (2.8)	8 (5.7)	.42
Aneurysm treatment			.86
Clipping	52 (47.7)	70 (49.6)	
Coiling	57 (52.3)	71 (50.5)	
Postprocedural CT, day after aneurysm treatment	1 (0 to 1)	1 (0 to 1)	.72
Infarct after aneurysm treatment	10 (9.2)	19 (13.5)	.39
Hemorrhage after aneurysm treatment	9 (8.3)	13 (9.2)	.97
**Vasospasm prophylaxis**			
Vasospasm prophylaxis with nimodipine	108 (99.1)	140 (99.3)	1
Vasospasm prophylaxis with Mg++	64 (58.7)	73 (51.8)	.33
Vasospasm prophylaxis with statins	17 (15.6)	22 (15.6)	1
**Cerebrospinal fluid drainage management**			
Start of recorded ICU treatment, day after SAH	2 (1 to 2)	2 (1 to 2)	.39
Patients with EVD	75 (68.8)	109 (77.3)	.17
Daily drainage via EVD, ml	92 (50 to 132)	171 (111 to 225)	< .001
Total Drainage via EVD, ICU day 1-8, ml	480 (172 to 793)	1222 (819 to 1662)	< .001
Patients with LD	109 (100)	7 (5)	< .001
Start of lumbar drainage, day after SAH	2 (1 to 2)	6 (2 to 8)	.01
Daily drainage via LD, ml	111 (101 to 124)	80 (51 to 98)	.008
Total Drainage via LD, ICU day 1-8, ml	827 (710 to 931)	100 (51 to 213)	< .001
Total CSF drainage, ICU day 1-8, ml [a]	1179 (825 to 1640)	1202 (776 to 1660)	.87
**Vasospasm assessment**			
Patients clinically suspect for vasospasm	28 (25.7)	47 (33.3)	.24
Patients with TCD vasospasm [b]	26 (26.3)	30 (24.8)	.93
Day of angiographic vasospasm assessment [c]	8 (7 to 10)	8 (7 to 10)	.47
Amount of angiographic vasospasm [c]			.78
no vasospasm	46 (56.1)	61 (56.5)	
up to 33%	10 (12.2)	18 (16.7)	
up to 66%	16 (19.5)	19 (17.6)	
more than 66%	10 (12.2)	10 (9.3)	
Endovascular rescue treatment	6 (5.5)	13 (9.2)	.39
**Hospital stay**			
Days in acute hospital, day after SAH	24 (19 to 30)	24 (18 to 31)	.83
Last imaging before discharge, day after SAH	17 (11 to 23)	17 (12 to 27)	.31
Final imaging modality			1
Computed tomography	99 (90.8)	129 (91.5)	
Magnetic resonance imaging	10 (9.2)	12 (8.5)	
Discharge location			.15
Home	37 (33.9)	32 (22.7)	
Rehabilitation	56 (51.4)	79 (56)	
other hospital	7 (6.4)	9 (6.4)	
died in acute hospital	9 (8.3)	21 (14.9)	
**Infections and VP shunts**			
Suspected infection of any cause	36 (33)	52 (36.9)	.62
VP shunt during acute care	22 (20.2)	34 (24.1)	.56
VP shunt in the first 6 months	28 (25.7)	42 (29.8)	.57
**Interview of surviving patients on day 180**			
Person who was queried [d]			.92
Patient	70 (72.9)	81 (70.4)	
Relative	20 (20.8)	26 (22.6)	
Healthcare professional	6 (6.2)	8 (7)	

Data are median (IQR) or n (%). Percentages might not total 100 because of rounding. [a] Numbers do not add up, as medians are reported, and some patients do not have both drains. [b] No TCD performed in 23 patients. [c] No angiography performed after aneurysm occlusion in 60 patients due to early death or local standard operating procedure. [d] Data on 240 surviving patients (3 missing data) were available. TCD = transcranial doppler

© 2023 Wolf S et al. *JAMA Neurology*.

eTable 16. Univariate Analysis of Factors Considered for Outcome Adjustment in the Per-Protocol Data

	Relative Risk	95% CI	P value	AIC
Age, per year increase	1.04	1.02 to 1.07	< .001	316.5
Female sex	1.01	0.69 to 1.39	.96	334.0
Hunt-Hess grade > 2	2.69	1.95 to 3.46	< .001	306.3
Hunt-Hess grade > 3	2.72	2.12 to 3.26	< .001	295.8
WFNS grade > 2	2.53	1.87 to 3.21	< .001	306.9
WFNS grade > 3	2.88	2.19 to 3.56	< .001	296.4
Modified Fisher grade 4	1.79	1.3 to 2.33	.001	322.8
Intracerebral hemorrhage	1.98	1.5 to 2.45	< .001	316.3
Intraventricular hemorrhage	2.08	1.49 to 2.73	< .001	318.4
Intracerebral or intraventricular hemorrhage	2.64	1.77 to 3.63	< .001	314.5
Posterior circulation aneurysm	0.85	0.5 to 1.28	.49	333.5

AIC = Akaike Information Criterion

Univariate analysis for outcome predictors in per-protocol data

For aneurysmal subarachnoid hemorrhage patients, known risk factors for worse outcome are age, and clinical and radiological severity grades. We analyzed the clinical data available on admission for association with the primary endpoint, modified Rankin Score larger than 2.

Clinical grading according to Hunt and Hess or WFNS scales showed collinearity. The same was found for radiological gradings. Models with lower Akaike Information Criterion values were chosen for multivariate analysis. Accordingly, in multivariate outcome assessment, adjustment for baseline imbalances was performed with the parameters age, Hunt-Hess grade larger than 3 and intracerebral or intraventricular hemorrhages (Table 2, main manuscript).

© 2023 Wolf S et al. *JAMA Neurology*.

eFigure 36. Mortality Analyzed Per Protocol

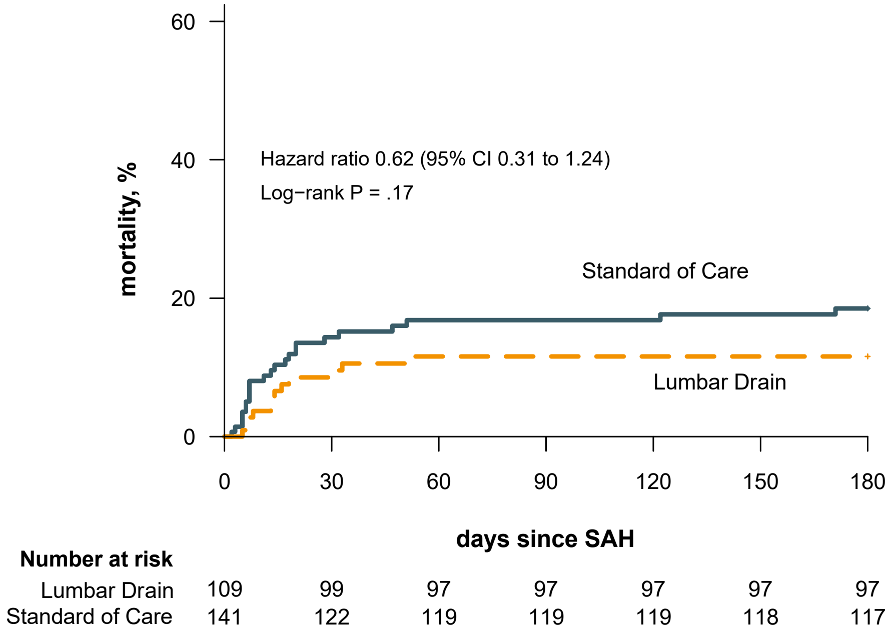

P value and confidence interval derived from Cox proportional hazard analysis, without adjustment for hemorrhage severity.

© 2023 Wolf S et al. *JAMA Neurology*.

eAppendix 3. As-Treated Analysis

For the as-treated analysis, patients were considered according to the treatment they received.

The Lumbar Drain group comprised of 111 patients, 109 initially randomized to receive a lumbar drain plus two additional patients randomized to Standard of Care, all with lumbar drain treatment.

The Standard of Care group in the as-treated analysis consists of the remaining 176 patients, 141 originally randomized to Standard of Care plus 35 patients crossing over after being randomized to Lumbar Drain, but receiving no or less than 480 ml lumbar drainage in the first week after aneurysm occlusion.

© 2023 Wolf S et al. *JAMA Neurology*.

eFigure 37. CONSORT Diagram for As-Treated Analysis

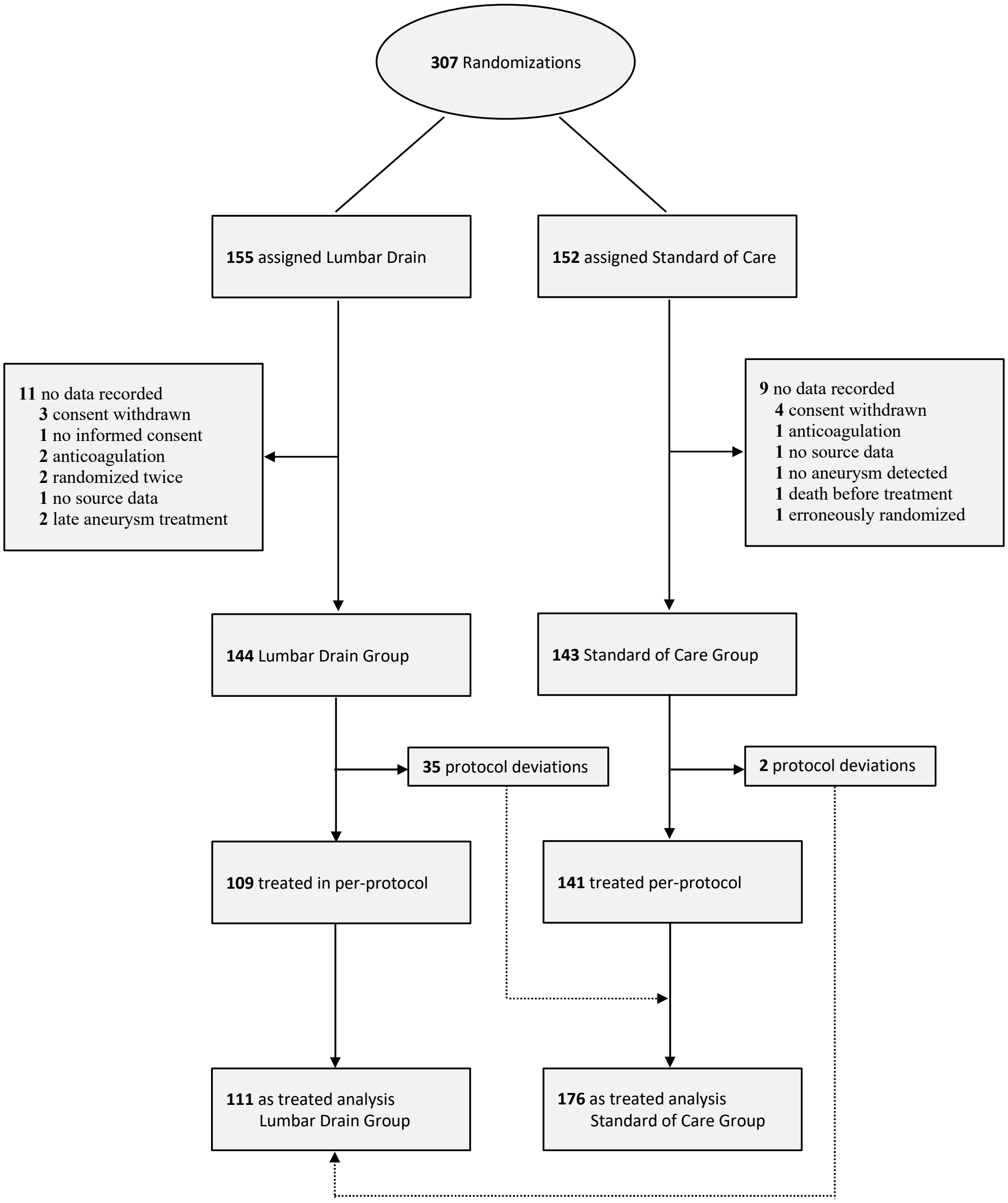

© 2023 Wolf S et al. *JAMA Neurology*.

eTable 17. Demographic Comparison Between Groups As Actually Treated

	Lumbar Drain (n = 111)	Standard of Care (n = 176)	P value
Age, years	54 (50 to 60)	56 (47 to 65)	.4
Sex			.39
Female	80 (72.1)	117 (66.5)	
Male	31 (27.9)	59 (33.5)	
Modified Rankin score on admission			1
0	104 (93.7)	165 (93.8)	
1	7 (6.3)	11 (6.2)	
Hunt-Hess classification			.17
1	24 (21.6)	30 (17)	
2	33 (29.7)	36 (20.5)	
3	18 (16.2)	41 (23.3)	
4	13 (11.7)	31 (17.6)	
5	23 (20.7)	38 (21.6)	
WFNS classification			.26
1	45 (40.5)	50 (28.4)	
2	13 (11.7)	30 (17)	
3	5 (4.5)	12 (6.8)	
4	10 (9)	19 (10.8)	
5	38 (34.2)	65 (36.9)	
Modified Fisher classification			.86
1	5 (4.5)	5 (2.8)	
2	5 (4.5)	7 (4)	
3	37 (33.3)	64 (36.4)	
4	64 (57.7)	100 (56.8)	
Intracerebral hemorrhage	40 (36)	66 (37.5)	.90
Intraventricular hemorrhage	69 (62.2)	106 (60.2)	.84
Aneurysm localisation			.38
ACA	10 (9)	15 (8.5)	
ACoA	35 (31.5)	56 (31.8)	
ICA	7 (6.3)	17 (9.7)	
MCA	19 (17.1)	41 (23.3)	
PCoA	17 (15.3)	26 (14.8)	
BA	9 (8.1)	11 (6.2)	
VA / cerebellar	14 (12.6)	10 (5.7)	
Number of aneurysms	1 (1 to 2)	1 (1 to 1)	.07
Size of aneurysm, mm [a]	5 (4 to 7)	6 (5 to 8)	.03
Aneurysm circulation			.07
Anterior	88 (79.3)	155 (88.1)	
Posterior	23 (20.7)	21 (11.9)	

Data are median (IQR) or n (%). Percentages might not total 100 because of rounding. [a] Aneurysm size was not available in 8 patients.

© 2023 Wolf S et al. *JAMA Neurology*.

eTable 18. Clinical Treatment Data Between Groups As Actually Treated

	Lumbar Drain (n = 111)	Standard of Care (n = 176)	P value
**Aneurysm treatment**			
Aneurysm treatment, day after SAH	1 (0 to 1)	1 (0 to 1)	.28
Recurrent SAH before treatment	3 (2.7)	14 (8)	0.11
Aneurysm treatment			1
Clipping	54 (48.6)	86 (48.9)	
Coiling	57 (51.4)	90 (51.1)	
Postprocedural CT, day after aneurysm treatment	1 (0 to 1)	1 (0 to 1)	.94
Infarct after aneurysm treatment	10 (9)	24 (13.6)	.32
Hemorrhage after aneurysm treatment	9 (8.1)	16 (9.1)	.94
**Vasospasm prophylaxis**			
Vasospasm prophylaxis with nimodipine	110 (99.1)	174 (98.9)	1
Vasospasm prophylaxis with Mg++	66 (59.5)	90 (51.1)	.21
Vasospasm prophylaxis with statins	18 (16.2)	28 (15.9)	1
**Cerebrospinal fluid drainage management**			
Start of recorded ICU treatment, day after SAH	2 (1 to 2)	2 (1 to 2)	.49
Patients with EVD	76 (68.5)	136 (77.3)	.13
Daily drainage via EVD, ml	91 (50 to 132)	162 (107 to 212)	< .001
Total drainage via EVD, ICU day 1-8, ml	472 (164 to 793)	1195 (715 to 1624)	< .001
Patients with LD	111 (100)	29 (16.5)	< .001
Start of lumbar drainage, day after SAH	2 (1 to 2)	2 (1 to 3)	.43
Daily drainage via LD, ml	111 (102 to 125)	58 (42 to 80)	< .001
Total drainage via LD, ICU day 1-8, ml	830 (720 to 933)	266 (100 to 385)	< .001
Total CSF drainage, ICU day 1-8, ml [a]	1179 (840 to 1632)	1177 (640 to 1621)	.43
**Vasospasm assessment**			
Patients clinically suspect for vasospasm	29 (26.1)	60 (34.1)	.2
Patients with TCD vasospasm [b]	27 (26.7)	40 (26.5)	1
Day of angiographic vasospasm assessment [c]	8 (7 to 10)	9 (7 to 10)	.25
Amount of angiographic vasospasm [c]			.93
no vasospasm	46 (55.4)	76 (54.7)	
up to 33%	11 (13.3)	22 (15.8)	
up to 66%	16 (19.3)	27 (19.4)	
more than 66% vasospasm	10 (12)	14 (10.1)	
Endovascular rescue treatment	7 (6.3)	17 (9.7)	.44
**Hospital stay**			
Days in acute hospital, day after SAH	23 (19 to 30)	24 (19 to 32)	.52
Last imaging before discharge, day after SAH	17 (11 to 23)	19 (12 to 28)	.08
Final imaging modality			.92
Computed tomography	101 (91)	162 (92)	
Magnetic resonance imaging	10 (9)	14 (8)	
Discharge location			.13
Home	38 (34.2)	40 (22.7)	
Rehabilitation	56 (50.5)	97 (55.1)	
other hospital	7 (6.3)	12 (6.8)	
died in acute hospital	10 (9)	27 (15.3)	
**Infection and VP shunt**			
Suspected infection of any cause	36 (32.4)	72 (40.9)	.19
VP shunt during acute care	22 (19.8)	46 (26.1)	.28
VP shunt in the first 6 months	28 (25.2)	55 (31.2)	.34
**Interview of surviving patients on day 180**			
Person who was queried [d]			.79
Patient	71 (73.2)	99 (69.2)	
Relative	20 (20.6)	33 (23.1)	
Healthcare professional	6 (6.2)	11 (7.7)	

Data are median (IQR) or n (%). Percentages might not total 100 because of rounding. [a] Numbers do not add up, as medians are reported, and some patients do not have both drains. [b] no TCD performed in 28 patients. [c] no angiography performed after aneurysm occlusion in 65 patients due to early death or local standard operating procedure. [d] Data on 240 surviving patients (3 missing data) were available.
TCD = transcranial doppler

© 2023 Wolf S et al. *JAMA Neurology*.

eTable 19. Univariate Analysis of Factors Considered for Outcome Adjustment in the As-Treated Data

	Relative Risk	95% CI	P value	AIC
Age, per year increase	1.04	1.02 to 1.07	< .001	367.3
Female sex	1.08	0.77 to 1.43	.64	386.8
Hunt-Hess grade > 2	2.58	1.94 to 3.23	< .001	355.2
Hunt-Hess grade > 3	2.85	2.31 to 3.32	< .001	335.6
WFNS grade > 2	2.62	2.01 to 3.22	< .001	351.0
WFNS grade > 3	2.9	2.28 to 3.48	< .001	339.7
Modified Fisher grade 4	1.77	1.33 to 2.24	< .001	374.0
Intracerebral hemorrhage	2.08	1.63 to 2.51	< .001	361.9
Intraventricular hemorrhage	1.99	1.47 to 2.54	< .001	370.0
Intraventricular or intracerebral hemorrhage	2.6	1.81 to 3.49	< .001	364.5
Posterior circulation aneurysm	0.86	0.52 to 1.27	.5	386.5

AIC = Akaike Information Criterion

Univariate analysis for outcome predictors in as-treated data

For aneurysmal subarachnoid hemorrhage patients, known risk factors for worse outcome are age, and clinical and radiological severity grades. We analyzed the clinical data available on admission for association with the primary endpoint, unfortunate outcome measured by a modified Rankin Score larger than 2.

Similar to the per-protocol data, clinical grading according to Hunt and Hess or WFNS scales as well as radiological grading showed collinearity. Models with lower Akaike Information Criterion values were chosen for multivariate analysis. Accordingly, in multivariate outcome assessment, adjustment for baseline imbalances was performed using the parameters age, Hunt-Hess grade larger than 3 and intracerebral or intraventricular hemorrhages (Table 2, main manuscript). These parameters were also used in regression models for assessment of the risk of infection for external ventricular drains and lumbar drains.

© 2023 Wolf S et al. *JAMA Neurology*.

eFigure 38. Mortality According to Actual Treatment

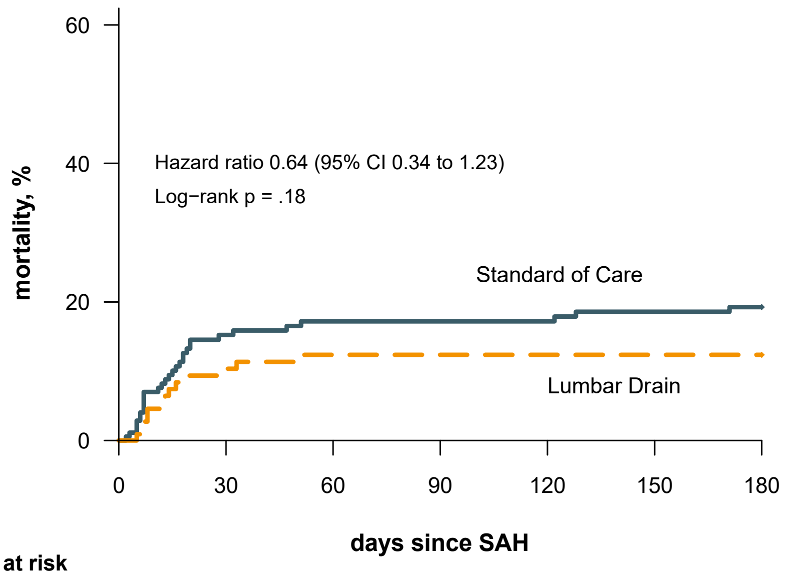

Number at risk							
Lumbar Drain	111	100	98	98	98	98	98
Standard of Care	176	151	148	148	148	146	145

P value and confidence interval from Cox proportional hazard analysis without adjustment for hemorrhage severity.

© 2023 Wolf S et al. *JAMA Neurology*.

eAppendix 4. Adverse Events

Vasospasm data is given in Table 2 in the main manuscript and eTables 3, 13, and 16 in this supplement.

The development of secondary infarctions other than being related to aneurysm occlusion was considered a key secondary endpoint in EARLYDRAIN. This data is available in Table 2 in the main manuscript and eTables 13 and 16. Further analysis on the relations of vasospasm, infarctions and modified Rankin scores at six months are in this appendix, eTables 4 to 9.

The requirement of a permanent cerebrospinal fluid shunt implantation for treatment of hydrocephalus was regarded an adverse event by some investigators and not separately reported by others. Numbers for permanent shunt necessity are given in Table 2 in the main manuscript and eTables 13 and 16 in this appendix.

One patient in each group was diagnosed with vitreous ocular hemorrhage, also known as Terson's syndrome.

One patient in the Lumbar Drain group was noted to have a local skin abscess at the lumbar punction site, requiring surgical excision.

One patient in the Lumbar Drain group had the lumbar catheter torn off when extraction was attempted, requiring surgery for removal of the remnant.

Patients with aneurysmal subarachnoid hemorrhage frequently experience a multitude of medical complications during their clinical course, including fever, pneumonia, electrolyte disturbances, cardiac failure, hypo- and hypertension. Per definition, these were not regarded as adverse events in EARLYDRAIN and investigators were encouraged to omit separate recording.

Data on the frequency infections is presented in Table 2 in the main manuscript and eTables 13 and 16. Analysis of risk factors for infections is given in eTables 20 and 21.

© 2023 Wolf S et al. *JAMA Neurology*.

eTable 20. Univariate Analysis of Potential Factors for the Development of Infection in the Clinical Course

	Relative Risk	95% CI	P value	AIC
Age, per year increase	1.00	0.99 to 1.02	.5	383.7
Female sex	0.99	0.7 to 1.33	.97	384.1
Hunt-Hess grade > 2	1.56	1.16 to 2.01	.006	376.3
Hunt-Hess grade > 3	1.44	1.07 to 1.82	.02	378.4
WFNS grade > 2	1.20	0.88 to 1.55	.23	382.7
WFNS grade > 3	1.31	0.97 to 1.68	.07	380.9
Modified Fisher grade 4	1.18	0.86 to 1.53	.29	383.0
Intracerebral hemorrhage	1.13	0.82 to 1.47	.43	383.5
Intraventricular hemorrhage	1.28	0.93 to 1.67	.13	381.7
Intraventricular or intracerebral hemorrhage	1.35	0.95 to 1.81	.1	381.3
Posterior circulation aneurysm	1.18	0.77 to 1.62	.41	383.4
Use of external ventricular drain	3.12	2.07 to 4.36	< .001	358.9
Use of lumbar drain	0.94	0.68 to 1.24	.68	383.9

Assessment of infection risk

Clinical suspicion of infection included but was not restricted to central nervous system involvement including device-associated meningitis. We analyzed the clinical factors possibly associated with the development of infection.

© 2023 Wolf S et al. *JAMA Neurology*.

eTable 21. Multivariate Analysis of Factors Associated With the Development of Infection

	Relative Risk	95% CI	P value
Age, per year increase	1.00	0.99 to 1.02	.69
Hunt-Hess > 2	1.22	0.73 to 1.97	.44
Intracerebral hemorrhage	0.90	0.55 to 1.44	.67
Intraventricular hemorrhage	0.96	0.57 to 1.54	.85
Use of external ventricular drain	3.20	1.91 to 4.88	< .001
Use of lumbar drain	1.02	0.65 to 1.57	.92

For multivariate assessment of the risk for the development of infections, we selected age, Hunt-Hess grade larger than 2 and intraventricular or parenchymal hemorrhage as predictors, according to the lowest values of the Akaike Information Criterion. Use of an external ventricular drain and use of a lumbar drain were forced into the multivariate statistical model, regardless of AIC in univariate analysis.

© 2023 Wolf S et al. *JAMA Neurology*.
