# Supplementary material for: Effectiveness of Lumbar Cerebrospinal Fluid Drain Among Patients With Aneurysmal Subarachnoid Hemorrhage: A Randomized Clinical Trial
Source: JAMA Neurol. 2023 Jun 18;80(8):833–42. doi: 10.1001/jamaneurol.2023.1792 (PMC10277935; doi:10.1001/jamaneurol.2023.1792)
Supplement: Supplement 3. — Group Information. EARLYDRAIN Study Group [file jamaneurol-e231792-s003.pdf]

Supplemental Online Content: Nonauthor Collaborators
*First name, last name, and suffix (if applicable) are required and will appear in PubMed.

*Group Name(s): EARLYDRAIN Study Group							
***First Name and Middle Initial(s)**	***Last Name**	***Suffix (eg, Jr, III)**	Academic Degrees	Institution	Location (city, state/province, country)	Role or Contribution, eg, chair, principal investigator	Group (if more than 1 Group listed in the byline) and/or Subgroup (eg, Steering Committee)
Norbert	Baro		n/a	Charite University Medicine	Berlin, Germany	data collection, randomizing	
Miriam	Bauer		n/a	Charite University Medicine	Berlin, Germany	data collection, randomizing	
Nora F.	Dengler		MD	Charite University Medicine	Berlin, Germany	data collection, randomizing	
Falk	von Dincklage		MD	Charite University Medicine	Berlin, Germany	data collection, randomizing	
Tobias	Finger		MD	Charite University Medicine	Berlin, Germany	data collection, randomizing	
Roland	Francis		MD	Charite University Medicine	Berlin, Germany	data collection, randomizing	
Benjamin	Hotter		MD	Charite University Medicine	Berlin, Germany	data collection, advice	
Oliver	Hunsicker		MD	Charite University Medicine	Berlin, Germany	data collection, advice	
Daniel	Jussen		MD	Charite University Medicine	Berlin, Germany	data collection, randomizing	
Eric	Jüttler		MD	Charite University Medicine	Berlin, Germany	advice	
Andreas	Schaumann		MD	Charite University Medicine	Berlin, Germany	data collection	
Jens	Witsch		MD	Charite University Medicine	Berlin, Germany	data collection, randomizing	
Christoph	Nagel		MD	St. Gertrauden Hospital	Berlin, Germany	local PI, data collection, randomizing	
Ullrich	Meier		MD	Unfallkrankenhaus	Berlin, Germany	advice	
Dino	Podlesik		MD	Carl-Gustav-Carus University	Dresden, Germany	data collection, randomizing	
Gabriele	Schackert		MD	Carl-Gustav-Carus University	Dresden, Germany	advice	
Hagen	Huttner		MD	Friedrich Alexander University	Erlangen, Germany	data collection	
Sabine	Hagedorn		n/a	University Hospital	Essen, Germany	data collection, randomizing	
Daniela	Müller		MD	University Hospital	Essen, Germany	data collection, randomizing	
Oliver	Müller		MD	University Hospital	Essen, Germany	data collection	
Robert	Sarge		MD	University Hospital	Essen, Germany	data collection	
Wolf-Dirk	Niesen		MD	Albert-Ludwig-University	Freiburg, Germany	data collection	
Katharina	Lange		n/a	Georg-August-Univerity	Göttingen, Germany	data collection	
Dennis	Päsler		MD	University Hospital	Greifswald, Germany	data collection, randomizing	
Stephanie	Reinhardt		MD	University Hospital	Greifswald, Germany	data collection, randomizing	
Jan	Regelsberger		MD	Eppendorf University Hospital	Hamburg, Germany	data collection	
Thomas	Sauvigny		MD	Eppendorf University Hospital	Hamburg, Germany	data collection	
Manfred	Westphal		MD	Eppendorf University Hospital	Hamburg, Germany	advice	
Rudolf	Gremmer		MD	St. Barbara Hospital	Hamm, Germany	local PI, data collection, randomizing	
Christian	Beyer		MD	Johannes Gutenberg University	Mainz, Germany	data collection, randomizing	
Desiree	Beyer		MD	Johannes Gutenberg University	Mainz, Germany	data collection	

Supplemental Online Content: Nonauthor Collaborators
*First name, last name, and suffix (if applicable) are required and will appear in PubMed.

***First Name and Middle Initial(s)**	***Last Name**	***Suffix (eg, Jr, III)**	Academic Degrees	Institution	Location (city, state/province, country)	Role or Contribution, eg, chair, principal investigator	Group (if more than 1 Group listed in the byline) and/or Subgroup (eg, Steering Committee)
Alexandra	Huthmann		n/a	Johannes Gutenberg University	Mainz, Germany	data collection	
Julia	Landscheidt		MD	Academic Hospital Bogenhausen	Munich, Germany	data collection	
David B.	Schul		MD	Academic Hospital Bogenhausen	Munich, Germany	data collection	
Yu-Mi	Ryang		MD	Klinikum Rechts der Isar	Munich, Germany	data collection, randomizing	
Elisabeth	Toeroek		MD	Klinikum Rechts der Isar	Munich, Germany	data collection, randomizing	
Wasim	Arouk		MD	Dietrich Bonhoeffer Klinikum	Neubrandenburg, Germany	data collection, randomizing	
Hosam	Al-Jehani		MD	McGill University	Montreal, Canada	data collection	
David B.	Sinclair		MD	McGill University	Montreal, Canada	data collection	
Christian	Fung		MD	Inselspital	Bern, Switzerland	data collection	
Nicole	Soell		MD	Inselspital	Bern, Switzerland	data collection	
Gerhard	Hildebrandt		MD	Cantonal Hospital	St. Gallen, Switzerland	advice	
Karen	Huscher		MD	Cantonal Hospital	St. Gallen, Switzerland	data collection, randomizing	
Heidrun	Lange		MD	Cantonal Hospital	St. Gallen, Switzerland	data collection, randomizing	
Peter	Hutchinson		MD	Addenbrooke's University Hospital	Cambridge, UK	data monitoring and safety	
Ming-Yuan	Tseng		MD	Addenbrooke's University Hospital	Cambridge, UK	data monitoring and safety	
