# Supplementary material for: Effectiveness of Lumbar Cerebrospinal Fluid Drain Among Patients With Aneurysmal Subarachnoid Hemorrhage: A Randomized Clinical Trial
Source: JAMA Neurol. 2023 Jun 18;80(8):833–42. doi: 10.1001/jamaneurol.2023.1792 (PMC10277935; doi:10.1001/jamaneurol.2023.1792)
Supplement: Supplement 4. — Data Sharing Statement [file jamaneurol-e231792-s004.pdf]

Data Sharing Statement

Wolf. Effectiveness of Lumbar Cerebrospinal Fluid Drain Among Patients With Aneurysmal Subarachnoid Hemorrhage. *JAMA Neurol*. Published June 18, 2023. doi:10.1001/jamaneurol.2023.1792

Data
Data available: Yes
Data types: Deidentified participant data
How to access data: Mendeley repository (doi:10.17632/c4jc7k5ptx.1)
When available: With publication

Supporting Documents
Document types: Other (please specify)
Additional Information: data dictionary with short explanation of variables
How to access documents: Mendeley repository (doi:10.17632/c4jc7k5ptx.1)
When available: With publication

Additional Information
Who can access the data: any researcher who is requesting the data
Types of analyses: Creative Commons Licence 4.0 applies
Mechanisms of data availability: without support, freely available
Any additional restrictions: Creative Commons Licence 4.0 applies
